# SIRT1 deficiency promotes age-related heart failure through enhancing ferroptosis via GATA4-HADHA-GPX4 axis

**DOI:** 10.1038/s41419-026-08634-z

**Published:** 2026-03-23

**Authors:** Yu Duan, Yingchun Luo, Xuejie Han, Hui Yu, Hanwen Liu, Yun Zhou, Yunlong Gao, Qian Xu, Ying Wei, Ruoxin Min, Yong Hong, Xuanrui Ji, Haibo Jia, Yue Li, Yun Zhang

**Affiliations:** 1 Department of Cardiology, the First Affiliated Hospital, Harbin Medical University, Harbin, China; 2 NHC Key Laboratory of Cell Transplantation, The First Affiliated Hospital of Harbin Medical University, Harbin, China; 3 Anhui Provincial Center For Clinical Laboratories, The First Affiliated Hospital of USTC, Hefei, China; 4 Department of Cardiology, 2nd Affiliated Hospital of Harbin Medical University, Harbin, China; 5 Key Laboratory of Cardiac Diseases and Heart Failure, Harbin Medical University, Harbin, China; 6 State Key Laboratory of Frigid Zone Cardiovascular Disease, Harbin Medical University, Harbin, Heilongjiang China

**Keywords:** Senescence, Cell death

## Abstract

Aging is a major contributor to the escalating prevalence of heart failure (HF). Ferroptosis has been implicated in age-related disorders and cardiovascular diseases. The role of ferroptosis in age-related HF remains unclear. Here, we show that aged rats exhibit impaired cardiac function accompanied by hallmark features of ferroptosis, including reduced glutathione peroxidase 4 (GPX4) expression and excessive lipid peroxidation. Consistently, cardiomyocyte-specific GPX4 knockout mice develop exacerbated cardiac ferroptosis and pronounced cardiac dysfunction. Iron overload further aggravates ferroptotic injury and cardiac dysfunction in aged rats, whereas pharmacological inhibition of ferroptosis markedly alleviates these effects. Conversely, cardiomyocyte-specific overexpression of GPX4 via rAAV9 attenuates ferroptosis and preserves cardiac function in D-galactose–induced aging mice. Proteomic analysis identifies hydroxyacyl-CoA dehydrogenase subunit A (HADHA) as a key protein markedly downregulated in aging hearts, particularly under iron overload. Mechanistically, HADHA deficiency induces mitochondrial dysfunction and excessive reactive oxygen species production, leading to glutathione depletion, GPX4 suppression, and subsequent ferroptosis. Accordingly, cardiomyocyte-specific knockdown of HADHA in young mice recapitulates ferroptosis-associated cardiac remodeling, which is reversed by ferrostatin-1 treatment. Furthermore, we identify SIRT1 (sirtuin 1) as an upstream regulator of HADHA during cardiac aging. Reduced SIRT1 expression in aging hearts suppresses HADHA transcription through inhibition of GATA4. Importantly, both cardiomyocyte-specific SIRT1 overexpression via rAAV9 in D-galactose–induced aging mice and pharmacological SIRT1 activation by resveratrol in aging rats restore HADHA expression, suppress ferroptosis, and protect against HF. Collectively, these findings establish ferroptosis as a critical contributor to age-related HF and identify the SIRT1–GATA4–HADHA axis as a potential therapeutic target.

## Introduction

Heart failure (HF) is a leading cause of morbidity and mortality worldwide, affecting an estimated 56 million people globally [1]. Advanced age is a major risk factor for HF. By 2050, the global population aged ≥65 years is projected to reach approximately 2 billion, with those aged ≥80 years increasing to 425 million [2], further amplifying the global burden of HF. Over the past few decades, the prevalence of HF has steadily increased, largely driven by population aging and extended life expectancy [3]. In the general adult population, HF prevalence ranges from 1% to 3%; however, this rate rises sharply to approximately 8% among individuals aged 65–74 years and further increases to 16.1% in those aged >74 years [4, 5]. With the rapid expansion of the aging population, age-related HF has emerged as a major global health challenge. Despite the urgency for effective therapeutic interventions, the precise mechanisms driving age-related HF remain unclear. This presents a critical knowledge gap in our current understanding, highlighting the need for further investigation.

The pathophysiology of age-related HF involves a complex interplay of molecular and cellular processes, many of which remain incompletely understood. Oxidative stress has long been recognized as an important feature of cardiac aging. With advancing age, reactive oxygen species (ROS) production increases, whereas antioxidant capacity declines, leading to disruption of cellular redox homeostasis and contributing to the development of age-related diseases [6, 7]. Excessive ROS accumulation has been shown to promote ferroptosis [8], a regulated form of cell death driven by iron-dependent lipid peroxidation [9].

Ferroptosis has emerged as a critical mechanism in multiple age-related disorders, including Alzheimer’s disease [10], Parkinson’s disease [11], osteoporosis, and osteoarthritis [12]. More recently, increasing evidence has linked ferroptosis to cardiovascular diseases [13], such as diabetic cardiomyopathy [14], doxorubicin-induced cardiomyopathy [15], abdominal aortic aneurysm [16], myocardial ischemia–reperfusion injury [17], and myocardial infarction [18]. Despite these advances, whether ferroptosis contributes to age-related HF and the mechanisms involved remain poorly defined, warranting further investigation.

In the present study, we sought to investigate the contribution of ferroptosis to the development of age-related HF and to identify its upstream regulatory mechanisms in the aging heart. Using multiple aging models, we found that ferroptotic injury is markedly enhanced during cardiac aging and is closely associated with metabolic dysregulation. Our study further suggests that the SIRT1–GATA4–HADHA axis plays a critical role in regulating ferroptosis susceptibility in cardiomyocytes during aging. Together, these findings provide a conceptual framework linking cardiac aging, metabolic remodeling, and ferroptosis in the pathogenesis of age-related HF.

## Materials and methods

### Experimental animals

The animal experiments in this study were performed in accordance with the Guide for the Care and Use of Laboratory Animals and approved by the Institutional Animal Care and Use Committee at Harbin Medical University (Ethical approval number: 2020123). Male Sprague–Dawley (SD) adult rats (200–250 g) were purchased from Beijing Vital River Laboratory Animal Technology Co., Ltd. (Beijing, China) and housed at the Experimental Animal Center of Harbin Medical University (Harbin, China). Cardiomyocyte-specific GPX4 knockout mice (GPX4-cKO) were generated by Cyagen Biosciences, Inc. (Suzhou, China). Briefly, GPX4-floxed mice (GPX4^flox/flox^) were maintained on a C57BL/6 J background. To generate experimental cohorts, GPX4^flox/flox^ mice were crossed with Myh6-Cre mice, and the resulting GPX4^flox/+^, Myh6-Cre progeny were further crossed with GPX4^flox/flox^ mice to produce GPX4^flox/flox^, Myh6-Cre (cardiomyocyte-specific GPX4 cKO) mice and littermate GPX4^flox/flox^, which served as floxed controls. The animals were kept in cages with 12 h light/dark cycles and fed standard chow and water ad libitum under specific pathogen-free (SPF) conditions.

### In vivo randomization and blinding procedures

Randomization and blinding procedures were implemented to minimize bias. Rats or mice were labeled according to the animal facility’s standard identification system and were then randomly assigned to experimental groups. Throughout the study, the investigator responsible for data acquisition was blinded to group allocation and/or genotyping information during the assessment of physiological parameters, including echocardiographic measurements, as well as during tissue collection. In addition, data analysis was performed by a second investigator who was also blinded to group allocation and/or genotyping. No animals were excluded from the analysis unless there was clear evidence of technical failure.

### Treatment with a high-iron diet

To evaluate the effect of iron supplementation on age-related HF, 18-month-old rats were randomly assigned to receive either a standard diet or a high-iron diet for 4 months. The high-iron diet contained 1.5 g/kg of iron, supplied as ferrous sulfate (FeSO₄) (Sichuan Longmang Group Co., Ltd). In parallel, 2-month-old rats were similarly randomized to receive the same high-iron diet for 4 months.

### Treatment with ferrostatin-1

To test the role of ferroptosis in age-related HF, 18-month-old rats were randomly divided into two groups. One group was intraperitoneally injected with the ferroptosis inhibitor ferrostatin-1 (0.8 mg/kg) once weekly for 4 months, whereas the vehicle control group was treated with saline of an equal volume.

### Treatment with acetylcysteine

For the acetylcysteine intervention experiment, 18-month-old rats were randomly divided into two groups. One group received acetylcysteine dissolved in drinking water (600 mg/L), whereas the other group received drinking water alone for 4 months. Furthermore, D-galactose was used to establish an aging rat model via subcutaneous injection at a dose of 150 mg/kg/day. Subsequently, 8-week-old rats were randomly divided into two groups. One group received acetylcysteine dissolved in drinking water (600 mg/L), whereas the other group received drinking water alone for 12 weeks.

### Treatment with resveratrol

To assess the potential of resveratrol in age-related HF, 18-month-old rats were randomly divided into two groups. One group received resveratrol (10 mg/kg/day) by gavage for 4 months, whereas the other group received an equal volume of control solvent.

### Construction and infection of recombinant serotype 9 adeno-associated virus (rAAV9)

For overexpression, recombinant serotype 9 adeno-associated virus (rAAV9) vectors with cardiomyocyte-specific promoters carrying GPX4/SIRT1 (rAAV9-GPX4/SIRT1) or negative control (rAAV9-NC) were constructed by Cyagen Biosciences (Suzhou, China). For in vivo infection, mice were injected with rAAV9-GPX4/SIRT1 or rAAV9-NC (5*10^11^ vg/mouse) via the tail vein. Accompanied with subcutaneous injection of D-galactose for 12 weeks.

To test the effects of HADHA, recombinant serotype 9 adeno-associated viruses (rAAV9) were used for HADHA knockdown in hearts. The rAAV9 coding murine shRNA-HADHA were purchased from Suzhou GenePharma Co., Ltd. 8-week-old mice were chosen to receive a single bolus tail vein injection of rAAV9-shHADHA or rAAV9-shNC at 1*10^12^ vg/mouse. Accompanied with intraperitoneal injection of Fer-1 for 12 weeks.

### Echocardiography

Transthoracic echocardiography was performed using a Vivid 7 ultrasound system (GE Healthcare, Milwaukee, WI, USA) with two-dimensional and M-mode analysis. Rats were anesthetized by intraperitoneal injection of 1% sodium pentobarbital (30 mg/kg), whereas mice were anesthetized with avertin (0.2 g/kg, intraperitoneally). During echocardiographic examination, heart rate was continuously monitored and maintained at approximately 400 ± 50 beats per minute in rats and 500 ± 50 beats per minute in mice to minimize the effects of anesthesia on cardiac function. The following parameters were measured: left ventricular systolic diameter (LVSd), interventricular septal thickness at systole (IVSs), left ventricular internal diameter at diastole (LVIDd) and systole (LVIDs), and left ventricular posterior wall thickness at diastole (LVPWd) and systole (LVPWs), left ventricular ejection fraction (LVEF), and left ventricular fractional shortening (LVFS).

### Histopathology

The ventricular tissue of rats was collected, fixed overnight in 4% paraformaldehyde, embedded in paraffin, and serially sectioned at 4 µm thickness. The tissues were then stained with hematoxylin and eosin (H&E) for routine histological examination. To measure collagen deposition, selected sections were stained with Masson’s trichrome. Fibrotic areas were quantified using ImageJ software, and collagen volume fraction was calculated as collagen area/total area × 100%.

### Analysis of oxylipins

Ventricular tissue samples were collected for targeted oxylipidomic analysis. Oxylipins were quantified using UHPLC–MRM–MS/MS on an EXIONLC System (SCIEX) coupled to a SCIEX 6500 QTRAP+ mass spectrometer equipped with an IonDrive Turbo V electrospray ionization (ESI) source. Chromatographic separation was performed on a 1.7 µm C18 column (150 × 2.1 mm). A targeted panel of 104 oxylipins was analyzed (Supplementary Table 1). During metabolite extraction, a mixture of stable isotope–labeled internal standards was added to each sample at known concentrations. Quantification was performed using an internal standard–based calibration approach, with calibration curves generated by least-squares linear regression. Calibration points with recoveries outside the 80–120% range were excluded. Tissue samples (~50 mg) were accurately weighed prior to homogenization, and final oxylipin concentrations were normalized to tissue mass and reported as ng/g tissue. Data acquisition and processing were conducted using SCIEX Analyst Workstation Software (v1.6.3) and MultiQuant 3.03.

### Proteomic analysis

Proteins were extracted from the samples and subjected to enzymatic digestion. The resulting peptides were labeled using tandem mass tags (TMT) according to the manufacturer’s instructions, allowing multiplexed quantitative analysis of up to 16 samples. Labeled peptides were fractionated by high-performance liquid chromatography (HPLC) and subsequently analyzed by liquid chromatography–tandem mass spectrometry (LC–MS/MS). Raw MS/MS data were processed and searched against the corresponding protein database using MaxQuant software (version 1.6.15.0). False discovery rate (FDR) control was applied during peptide–spectrum matching and protein identification. Differentially expressed proteins were identified based on a *P* value ≤ 0.05 combined with a fold-change threshold of >1.2 for upregulation or <1.2 for downregulation. These criteria were used to generate the volcano plot.

### Culture of primary rat cardiomyocytes and fibroblasts

Primary cardiomyocytes were isolated from the hearts of neonatal Sprague–Dawley rats (1–3 days old). Briefly, the hearts were aseptically dissected and cut into small pieces. The tissue was then digested in 0.25% trypsin with gentle shaking, and the digestive solution was collected in DMEM supplemented with 10% fetal bovine serum, followed by centrifugation at 1200 rpm for 5 min. The cell pellet was then resuspended in DMEM containing 10% FBS and 1% penicillin–streptomycin. The cells were transferred to a culture dish and allowed to adhere for 90 min. Non-adherent cells, which primarily contained cardiomyocytes, were then transferred to new six-well plates and incubated at 37 °C in a 5% CO₂ atmosphere. Upon reaching a confluence of 70–80% and exhibiting good viability, treatments were initiated. Hydrogen peroxide (H₂O₂; 50 μmol/L) was used to establish a senescent cell model.

### MTT assay

MTT assay was performed to assess cell viability utilizing the MTT Cell Proliferation Assay (Beyotime, Shanghai, China), following the manufacturer’s instructions. Briefly, cells were cultured in 96-well plates. After the designated treatments, 10 μL of MTT reagent was added to each well containing phenol red-free culture medium, followed by an incubation period of 4 h at 37 °C. Subsequently, DMSO was added to the cells, and the absorbance was measured using a microplate reader (Thermo, MA, USA).

### JC-1 staining

The mitochondrial membrane potential (MMP) was detected using the JC-1 staining kit (Beyotime) in accordance with the manufacturer’s instructions. Briefly, primary rat cardiomyocytes were incubated with JC-1 staining solution (5 μg/mL) at 37 °C for 20 min. Subsequently, the cells were carefully rinsed with JC-1 staining buffer to remove excess dye. Mitochondrial depolarization was indicated by an increase in the green/red fluorescence intensity ratio.

### Intracellular reactive oxygen species (ROS) detection

Intracellular levels of ROS were determined using dihydroethidium (DHE; S0063, Beyotime). In brief, primary rat cardiomyocytes were incubated with 5 μM dihydroethidium in serum-free medium at 37 °C for 30 min in the dark. After incubation, cells were washed thoroughly to remove unincorporated dye, followed by examination under an immunofluorescence microscope (Zeiss, Jena, Germany).

### Western blot

Total protein samples were extracted from rat tissues or primary cultured cardiomyocytes and cardiac fibroblasts. Briefly, ~30–50 μg of protein was separated by 8–12% SDS-PAGE. Proteins were transferred to PVDF membranes (Millipore, Billerica, MA, USA). The membranes were then incubated with primary antibodies against TF (1:500, Proteintech, cat#17435-1-AP, RRID: AB_2035023), FTH1 (1:500, Bioss, cat#bs-5907R, RRID: AB_11050926), GPX4 (1:500, Abcam, cat#ab125066, RRID: AB_10973901), HADHA (1:2000, Proteintech, cat#10758-1-AP, RRID: AB_2115593), and SIRT1 (IP, 1:500, Proteintech, cat#60303-1-Ig, RRID: AB_2881417; IB, 1:500, Abcam, cat#ab189494, RRID: AB_2864311), GATA4 (1:500, Proteintech, cat#19530-1-AP, RRID: AB_10642003), and GAPDH (1:10000, Abcam, cat#ab128915, RRID: AB_2747414) at 4°C overnight. After washing, the membranes were incubated with anti-IgG horseradish peroxidase-conjugated secondary antibodies (Jackson Immuno Research, West Grove, PA, USA). The membranes were exposed to ECL buffer and detected using the ChemiDoc XRS gel documentation system (Bio-Rad, Hercules, CA, USA). Protein bands were analyzed using Bio-Rad software and normalized to the internal reference.

### Co-immunoprecipitation

Cardiomyocytes were transfected with negative control or si-SIRT1 plasmids. A total of 200 μL 1× IP lysis buffer (containing protease inhibitors) was added to the collected cardiomyocytes, and the cells were lysed on ice for 15 min, followed by centrifugation at 13,500 × *g* for 15 min to obtain the supernatant. Subsequently, the precleared lysates were mixed with primary anti-Sirt1 antibody. The mixture was gently shaken at 4 °C and incubated overnight. Protein A/G beads were washed once with lysis buffer and collected by magnetic separation. Then, 20 μL of protein A/G beads were added to the mixture and gently shaken at 4 °C for 2 h. The samples were washed three times with lysis buffer, and the supernatant was carefully removed by magnetic separation. A total of 24 μL of lysis buffer and 6 μL of 5× SDS sample buffer were added, and the samples were boiled for 10 min. The protein A/G beads were discarded by magnetic separation, and the supernatant was collected. Finally, 15 μL of each sample was separated by SDS-PAGE for Western blot analysis.

### Statistical analysis

All statistical evaluations were conducted using GraphPad Prism (version 9.0; GraphPad Software, La Jolla, CA, USA). Continuous data were presented as the mean ± standard error of the mean (SEM) or as the median with interquartile range, depending on data distribution. Categorical data were summarized as frequencies and corresponding percentages. The sample size (n) for each statistical analysis was provided in the figure legends. To assess normality, the Shapiro–Wilk test was applied. Comparisons between two independent groups were performed using an unpaired Student’s t-test for normally distributed variables or the Wilcoxon rank-sum test (also known as the Mann–Whitney U test) for non-normally distributed data. Variables with more than two groups were analyzed by one-way ANOVA, followed by Tukey’s post hoc test. A *P*-value less than 0.05 was considered statistically significant.

## Results

### GPX4 deficiency accelerates age-related heart failure in mice

**Fig. 1:**
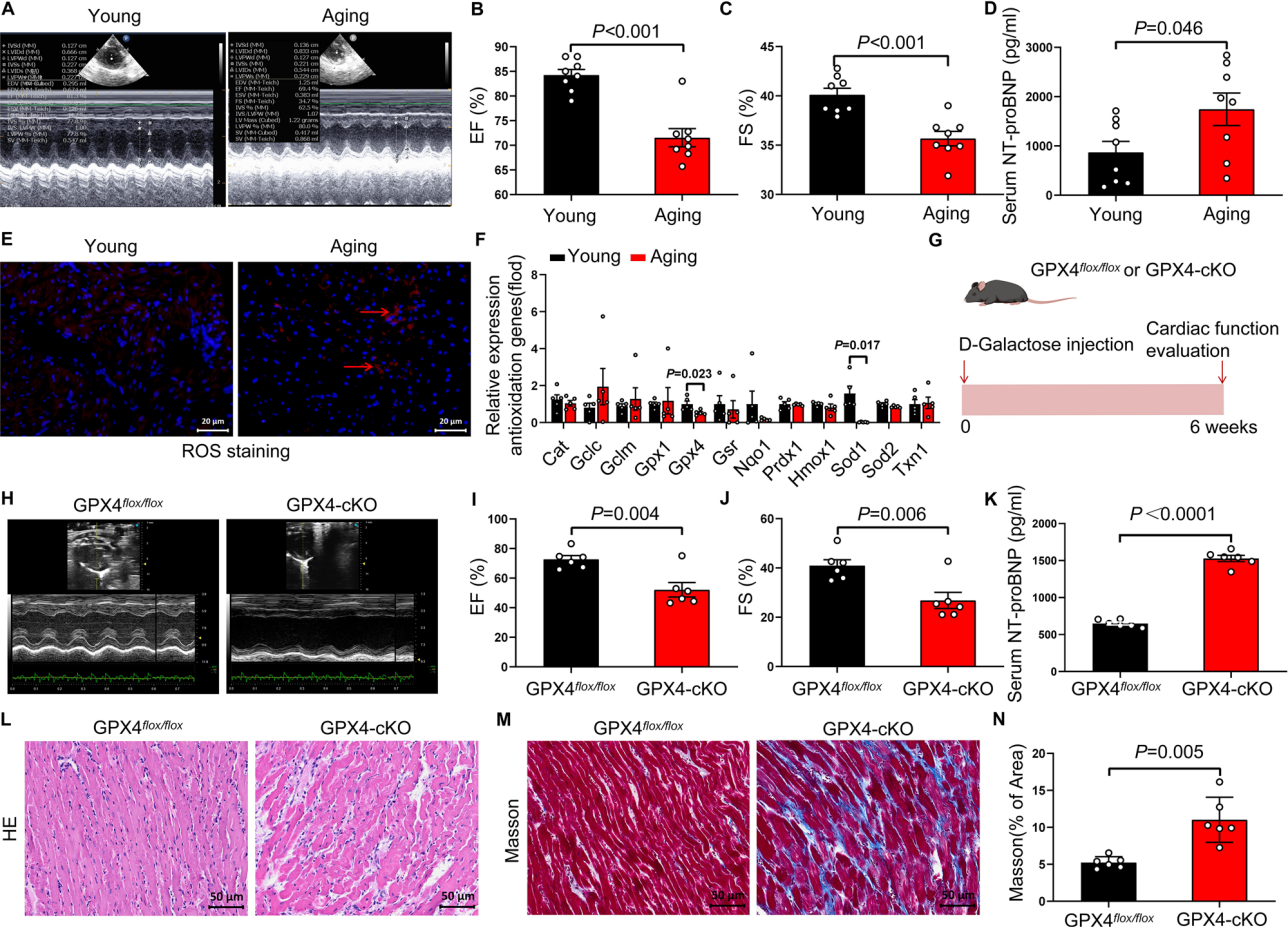
Aging rats and cardiomyocyte-specific Gpx4 knockout mice displayed declined cardiac function. **A** Representative M-mode echocardiographic images of left ventricular wall motion in young and aging rats. **B** The statistical data of cardiac ejection fraction (EF) of young rats and aging rats (*n* = 8 per group). **C** The statistical data of cardiac fractional shortening (FS) of young and aging rats (*n* = 8 per group). **D** Quantitative analysis of serum NT-proBNP levels in young and aging rats (*n* = 8 per group). **E** Representative images of reactive oxygen species (ROS) staining in the left ventricle of young and aging rats. Scale bars, 20 μm. **F** Relative mRNA expression of antioxidant-related genes in left ventricular tissue from young and aging rats, assessed by quantitative RT-PCR (*n* = 5 per group). **G** Schematic illustration of the experimental design. Aging was induced by subcutaneous injection of D-galactose (200 mg/kg/day) for 6 weeks in cardiomyocyte-specific GPX4 knockout (GPX4-cKO) mice and their littermate controls (GPX4^flox/flox^). **H** Representative M-mode echocardiographic images of mice from GPX4^flox/flox^ group and GPX4-cKO group. **I** The statistical data of left ventricular ejection fraction (EF) of mice from GPX4^flox/flox^ group and GPX4-cKO group (*n* = 6 per group). **J** The statistical data of left ventricular fractional shortening (FS) of mice from GPX4^flox/flox^ group and GPX4-cKO group (*n* = 6 per group). **K** The levels of serum NT-ProBNP in mice from GPX4^flox/flox^ group and GPX4-cKO group (*n* = 6 per group). **L** Representative images of HE staining of the left ventricle from GPX4^flox/flox^ group and GPX4-cKO group. Scale bars, 50 μm. **M** Representative images of Masson staining of the left ventricle of mice from GPX4^flox/flox^ group and GPX4-cKO group. Scale bars, 50 μm. **N** The collagen volume fraction of the left ventricle of mice from GPX4^flox/flox^ group and GPX4-cKO group (*n* = 6 per group). The data are given as mean ± SEM and compared by Student’s t test.

Aging is a major risk factor for heart failure. To characterize age-related changes in cardiac function, we performed echocardiographic analyses in 6-month-old (young) and 22-month-old (aging) rats. Compared with young rats, aging rats exhibited a significant reduction in left ventricular ejection fraction (EF) and fractional shortening (FS) (Fig. 1A–C), increased IVSd, IVSs, LVIDd, and LVPWd (Supplementary Table 2), accompanied by a marked increase in serum NT-proBNP levels (Fig. 1D), indicating impaired cardiac function during aging. Given the established association between oxidative stress and age-related diseases [19, 20], we next assessed myocardial oxidative status. Reactive oxygen species (ROS) levels were significantly elevated in the hearts of aging rats compared with young controls (Fig. 1E). To further examine alterations in antioxidant defense systems, we analyzed the expression of genes involved in ROS detoxification. Among these, the expression of glutathione peroxidase 4 (Gpx4) and superoxide dismutase 1 (Sod1) was significantly reduced in aging rat hearts (Fig. 1F).

Since GPX4 plays a crucial role in mitigating lipid peroxidation and protecting against oxidative stress [21, 22], we focused on investigating its potential involvement in age-related cardiac dysfunction. To this end, we generated cardiomyocyte-specific GPX4 knockout (GPX4-cKO) mice and assessed the impact of GPX4 deficiency on cardiac function in a D-galactose–induced aging mouse model (Fig. 1G). As expected, GPX4 conditional knockout significantly downregulated EF, FS, IVSd, IVSs and LVIDd (Fig. 1H–J, Supplementary Table 2), increased serum NT-proBNP levels (Fig. 1K), and aggravated cardiac structural disorganization, as well as and increased cardiac fibrosis levels in aging mice (Fig. 1L–N). To further validate the role of GPX4 in age-related heart failure, cardiomyocyte GPX4 overexpression was induced in D-galactose–induced aging mice via rAAV9-mediated delivery of a cTnT promoter–driven GPX4 construct (Fig. S1A). This intervention significantly improved cardiac function, as evidenced by increased ejection fraction (EF) and fractional shortening (FS) and reduced serum NT-proBNP levels (Fig. S1B–D). Histological analyses further revealed attenuated myocardial structural disruption and reduced interstitial fibrosis following GPX4 overexpression (Fig. S1E–G). Together, these results suggest an important role for GPX4 in the development of age-related heart failure.

### Aging rats displayed ferroptosis-associated changes in the heart

**Fig. 2:**
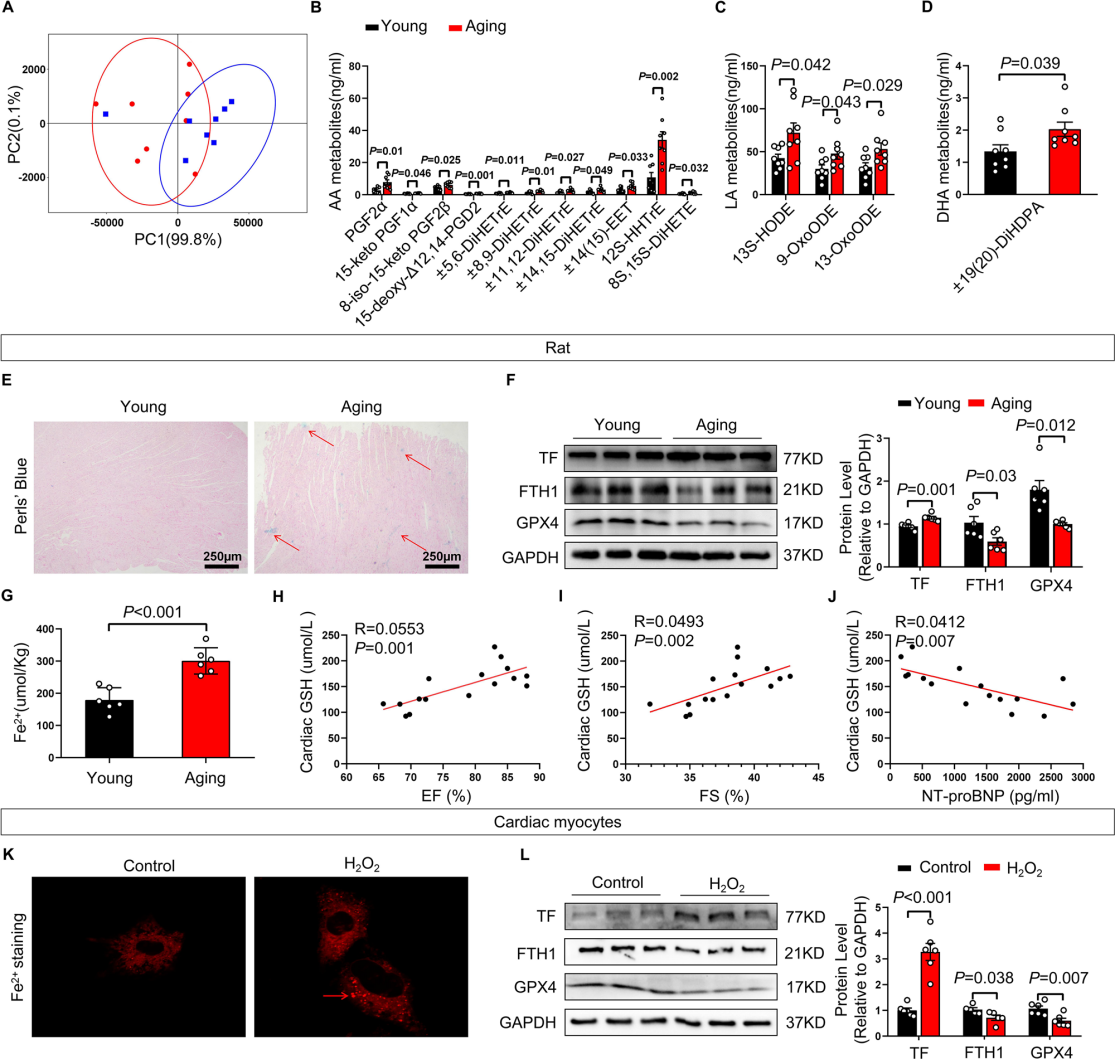
Aging rats displayed increased oxidative stress and aggravated ferroptosis. **A** Principal component analysis (PCA) of oxidized fatty acid metabolites in the left ventricle of rats from Young and aging group (*n* = 8 per group). **B**–**D** Quantitative analysis of arachidonic acid (AA) metabolites, linoleic acid (LA) metabolites and docosahexaenoic acid (DHA) metabolites in young and aging rats (*n* = 8 per group). **E** Representative image of Perls’ Blue staining in the left ventricle of young and aging rats. Scale bars, 250 μm. **F** Representative bands and quantification of expressions of TF, FTH1 and GPX4 in heart of young and aging rats (*n* = 6 per group). **G** The levels of Fe^2+^ in the heart of young and aging rats (*n* = 6 per group). **H** Correlation between cardiac glutathione (GSH) levels and left ventricular ejection fraction (EF) (*n* = 16). **I** Correlation between cardiac glutathione (GSH) levels and left ventricular fractional shortening (FS) (*n* = 16). **J** Correlation between cardiac glutathione (GSH) levels and serum NT-proBNP concentrations (*n* = 16). **K** Representative images of FerroOrange detection in cardiomyocytes of Control group and H_2_O_2_ group. **L** Representative bands and quantification of the expression of TF, FTH1, and GPX4 in cardiomyocytes of Control group and H_2_O_2_ group (*n* = 6 per group). The data are given as mean ± SEM and compared by Student’s t test.

Ferroptosis is characterized by the peroxidation of polyunsaturated fatty acids (PUFAs), which gives rise to bioactive oxidized lipid species. To assess age-related changes in lipid peroxidation, we performed oxidized lipidomics analysis of ventricular tissues. Aging rats exhibited markedly increased levels of multiple oxidized fatty acid metabolites derived from arachidonic acid and linoleic acid compared with young controls (Fig. 2A–D). Consistent with these findings, aging rat hearts showed increased iron deposition and elevated expression of transferrin (TF), accompanied by reduced expression of ferritin heavy chain 1 (FTH1) and glutathione peroxidase 4 (GPX4) (Fig. 2E–G). To examine the relationship between ferroptosis and cardiac function, correlation analyses were performed between myocardial glutathione (GSH) levels and cardiac functional parameters. Myocardial GSH levels were positively correlated with ejection fraction (EF) and fractional shortening (FS), and negatively correlated with serum NT-proBNP levels (Fig. 2H–J).

To further evaluate whether these alterations were recapitulated at the cellular level, we established a senescent cardiomyocyte model using H₂O₂ (Fig. S2A, B). Senescent cardiomyocytes exhibited increased reactive oxygen species (ROS) production and iron accumulation compared with control cells (Figs. S2C and 2K), along with increased TF expression and reduced FTH1 and GPX4 levels (Fig. 2L). Similar changes were observed in a D-galactose–induced cellular senescence model, which showed increased ROS levels, accompanied by the upregulation of TF and the downregulation of FTH1 and GPX4 (Fig. S3A–D). Together, these findings demonstrate age-related alterations in lipid peroxidation, iron handling, and ferroptosis-associated molecular signatures in the heart.

### Modulation of ferroptosis improves cardiac function in aging rats

**Fig. 3:**
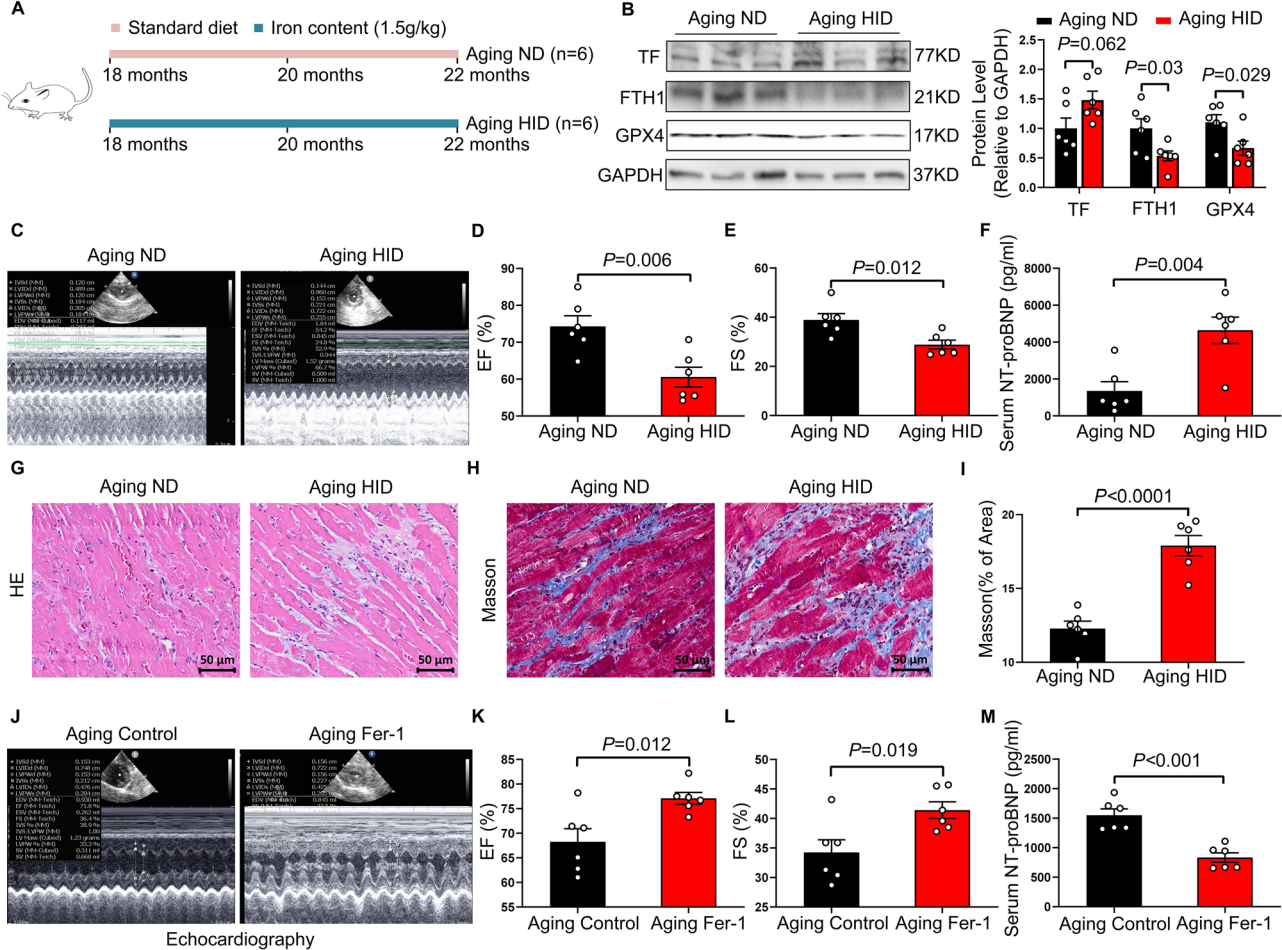
A high-iron diet induces cardiac dysfunction and structural remodeling in aging rats, which is attenuated by ferroptosis inhibition. **A** Schematic illustration of the experimental design. Eighteen-month-old rats were randomly assigned to receive a standard diet (ND) or a high-iron diet (HID) for 4 months. **B** Representative Western blot bands and quantification of transferrin (TF), ferritin heavy chain 1 (FTH1), and GPX4 expression in cardiac tissue from Aging ND group and Aging HID group (*n* = 6 per group). **C** Representative M-mode echocardiographic images of the left ventricle in ND-fed and HID-fed aging rats. Left ventricular ejection fraction (EF) (**D**) and fractional shortening (FS) (**E**) in aging ND and aging HID rats (*n* = 6 per group). **F** Serum NT-proBNP levels in aging ND and aging HID rats (*n* = 6 per group). **G** Representative hematoxylin and eosin (H&E)–stained sections of left ventricular tissue from ND-fed and HID-fed aging rats. Scale bars, 50 μm. **H** Representative Masson trichrome–stained sections of left ventricular tissue. Scale bars, 50 μm. **I** Quantification of left ventricular collagen volume fraction in aging ND and aging HID rats (*n* = 6 per group). **J** Representative M-mode echocardiographic images of the left ventricle in aging control and aging ferrostatin-1 (Fer-1)–treated rats. Left ventricular ejection fraction (EF) (**K**) and fractional shortening (FS) (**L**) in aging control and aging Fer-1–treated rats (*n* = 6 per group). **M** Serum NT-proBNP levels in aging control and aging Fer-1–treated rats (*n* = 6 per group). The data are given as mean ± SEM and compared by Student’s t test.

To investigate the role of ferroptosis in age-related HF, 18-month-old rats were fed a high-iron diet for 4 months (Fig. 3A). Iron supplementation markedly reduced FTH1 and GPX4 levels in the hearts of aging rats (Fig. 3B). Compared with controls, aging rats receiving a high-iron diet exhibited significantly reduced EF and FS, along with elevated serum NT-proBNP levels (Fig. 3C–F). Consistently, the high-iron diet led to aggravated myocardial structural disorganization and increased cardiac fibrosis (Fig. 3G–I).In contrast, administration of a high-iron diet to young rats did not significantly affect cardiac function or ferroptosis-associated markers (Fig. S4A-F). To further examine whether inhibition of ferroptosis could mitigate age-related cardiac dysfunction, 18-month-old rats were treated with the ferroptosis inhibitor ferrostatin-1 (Fer-1) (Fig. S5A). Fer-1 treatment reduced TF expression and restored FTH1 and GPX4 levels in the cardiac tissues of aged rats (Fig. S5B). Moreover, Fer-1–treated aging rats exhibited improved cardiac function, as evidenced by increased EF and FS and reduced serum NT-proBNP levels compared with untreated controls (Fig. 3J–M). These findings suggest that ferroptosis is a critical pathological mechanism underlying age-related HF.

### HADHA is downregulated in aging hearts and modulates ferroptosis-associated pathways

**Fig. 4:**
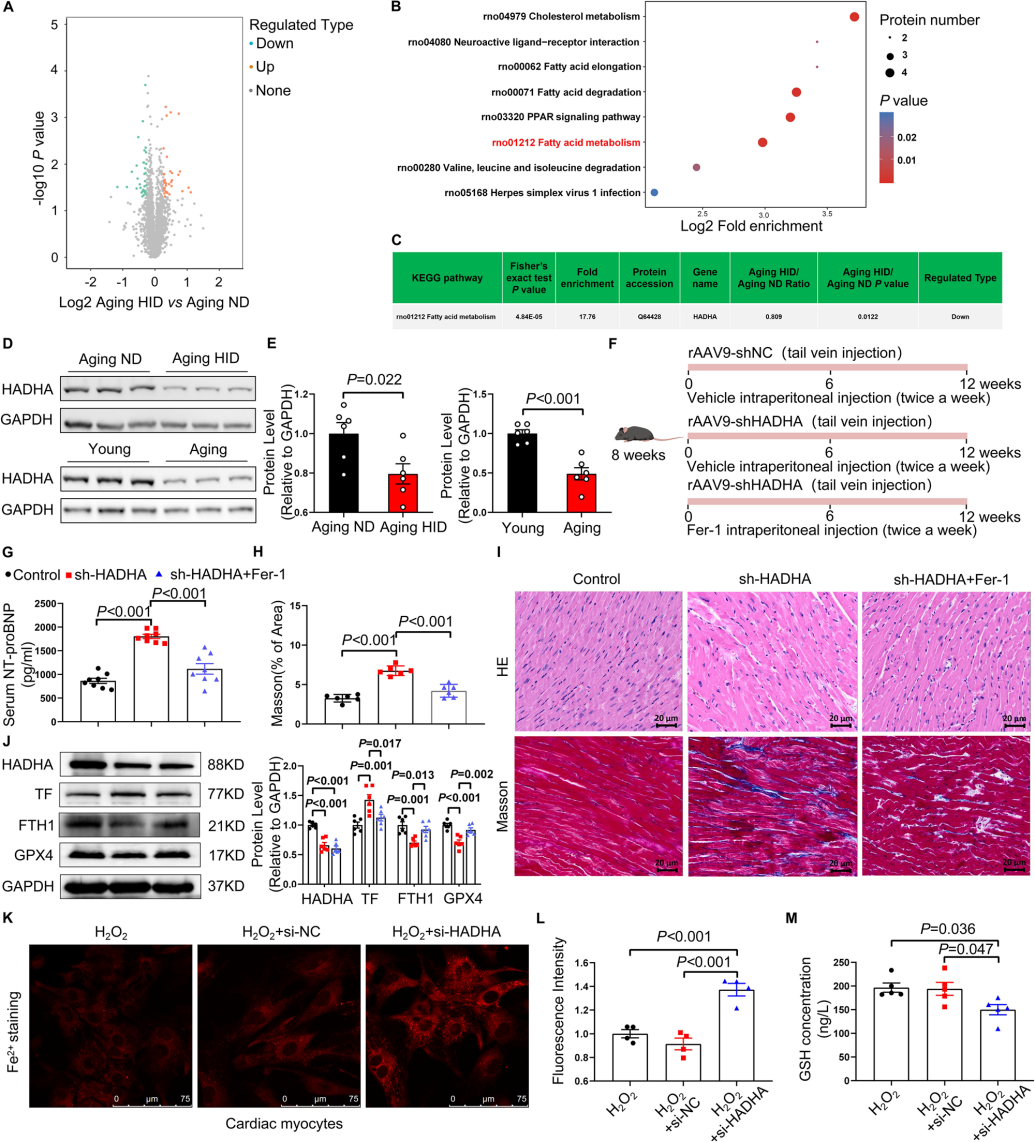
HADHA deficiency in aging rats contributes to ferroptosis. **A** Volcano plot showing differentially expressed proteins in cardiac tissue from aging rats fed a normal diet (ND) or a high-iron diet (HID), as determined by proteomic analysis (*n* = 3 biological replicates per group). **B** KEGG pathway enrichment analysis of differentially expressed proteins, showing the top 8 enriched pathways in the hearts of ND-fed and HID-fed aging rats. **C** Relative abundance of HADHA in cardiac tissue from ND-fed and HID-fed aging rats as determined by proteomics. **D** Representative immunoblot images showing HADHA expression in cardiac tissue from aging ND and aging HID rats, as well as from young and aging rats. **E** Quantification of HADHA protein levels in the hearts of aging ND and aging HID rats (*n* = 6 per group), as well as from young and aging rats (*n* = 6 per group). **F** Schematic illustration of the experimental design. Eight-week-old C57BL/6 mice were randomly assigned to three groups and received a single tail vein injection of AAV9 encoding control shRNA (AAV9-shNC), cardiomyocyte-specific HADHA shRNA (AAV9-shHADHA), or AAV9-shHADHA combined with ferroptosis inhibition. Ferrostatin-1 (Fer-1) was administered by intraperitoneal injection twice weekly for 12 weeks. **G** Serum NT-proBNP levels in mice from each groups (*n* = 8 per group). **H** Quantification of left ventricular collagen volume fraction in mice (*n* = 6 per group). **I** Representative hematoxylin and eosin (H&E)–stained sections and Masson trichrome–stained sections of left ventricular tissue from mice. Scale bars, 20 μm. **J** Representative immunoblot images and quantification of HADHA, TF, FTH1, and GPX4 expression in cardiac tissue from mice (*n* = 6 per group). **K**, **L** Cultured cardiomyocytes were transfected with HADHA-silencing siRNA (si-HADHA) or control siRNA. After 24 h, cells were exposed to H₂O₂ for an additional 24 h before subsequent analyses. **K** Representative images of FerroOrange staining showing intracellular ferrous iron (Fe²⁺) levels in cardiomyocytes. Scale bars, 75 μm. **L** Quantification of FerroOrange fluorescence intensity (*n* = 4 per group). **M** Intracellular glutathione (GSH) levels in cardiomyocytes (*n* = 5 per group). Data are presented as mean ± SEM. Statistical analyses were performed using Student’s t test or one-way ANOVA.

To explore the molecular mechanisms underlying ferroptosis in aging hearts, we performed proteomic profiling of cardiac tissues from aging rats fed either a normal diet or a high-iron diet. Comparative analysis revealed marked differences in protein expression between the two groups (Fig. 4A). Kyoto Encyclopedia of Genes and Genomes (KEGG) pathway analysis identified fatty acid metabolism as the most significantly enriched pathway among the differentially expressed proteins (Fig. 4B). Notably, the expression of hydroxyacyl-CoA dehydrogenase subunit alpha (HADHA) was significantly reduced in the hearts of aging rats receiving a high-iron diet (Fig. 4C). Reduced HADHA expression was further confirmed in the hearts of aging rats under both normal and high-iron dietary conditions (Fig. 4D–E). To further investigate the role of HADHA in age-related cardiac ferroptosis and dysfunction, cardiomyocyte-specific knockdown of HADHA was achieved in young mice via rAAV9-mediated gene delivery (Fig. 4F). Compared with control mice, HADHA-deficient mice exhibited significantly elevated serum NT-proBNP levels, and unchanged EF and FS (Figs. S6A–C and 4G). Histological analyses further revealed pronounced myocardial structural disorganization and exacerbated cardiac fibrosis (Fig. 4H–I). Furthermore, HADHA knockdown led to decreased HADHA expression, increased TF expression and reduced levels of FTH1 and GPX4 (Fig. 4J). Importantly, pharmacological inhibition of ferroptosis with Fer-1 markedly attenuated HADHA knockdown–induced cardiac ferroptosis and pathological remodeling.

HADHA deficiency has been reported to cause mitochondrial dysfunction, which in turn triggers the accumulation of ROS [23]. Interestingly, we discovered that silencing HADHA led to increased ROS levels and decreased mitochondrial membrane potential (MMP) in H_2_O_2_-induced senescent cardiomyocytes (Fig. S7A-C), accompanied by decreased cell viability and ATP levels (Fig. S7D-E). In parallel, HADHA silencing increased intracellular iron accumulation (Fig. 4K, L), upregulated TF expression, and reduced FTH1 and GPX4 levels (Fig. S7F). Myocardial glutathione (GSH) levels were also significantly reduced following HADHA knockdown (Fig. 4M). In contrast, HADHA silencing did not significantly affect ferroptosis-associated markers in senescent cardiac fibroblasts (Fig. S8A, B). Supporting a cardiomyocyte-specific relevance, single-cell RNA sequencing analysis of human cardiac tissues from young and elderly donors revealed that ferroptosis-related pathways were among the most significantly age-enriched pathways in cardiomyocytes, whereas no significant enrichment was observed in fibroblasts, endothelial cells, or immune cells (Fig. S8C). Furthermore, HADHA overexpression reversed ROS accumulation and restored cell viability, GSH content, and ATP levels in H₂O₂-induced senescent cardiomyocytes (Fig. S9A–E).

### HADHA deficiency induces ferroptosis through regulation of GSH-GPX4

**Fig. 5:**
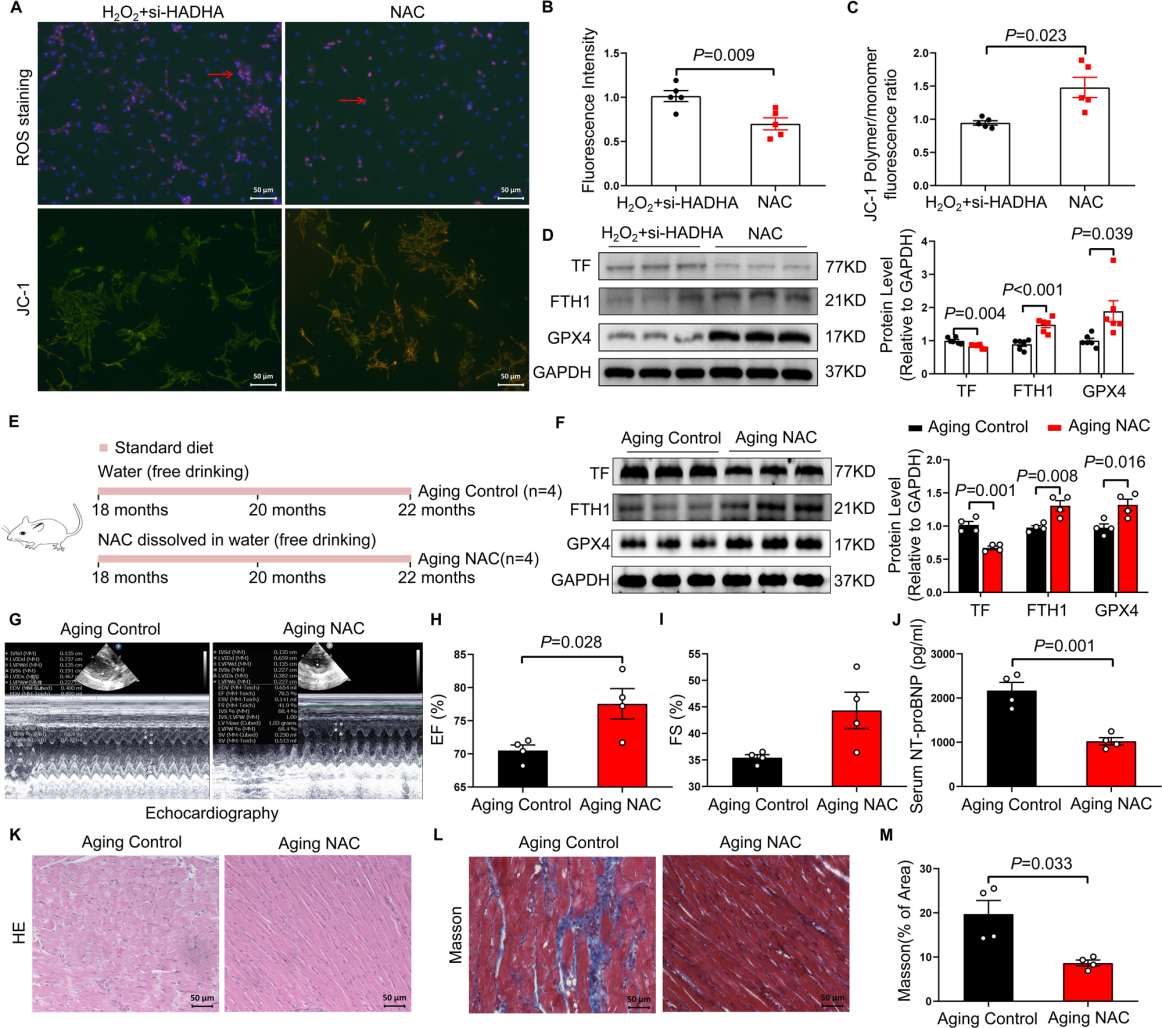
NAC supplementation restrains ferroptosis in cardiomyocytes and protects aging rats from cardiac dysfunction. **A**–**D** Cultured cardiomyocytes were transfected with HADHA-silencing small interfering RNA (si-HADHA) or control siRNA. After 24 h, cells were exposed to H₂O₂ for an additional 24 h. Following replacement of the culture medium, cells were treated with vehicle or N-acetylcysteine (NAC) for 24 h before subsequent analyses. **A** Representative images of reactive oxygen species (ROS) staining and JC-1 staining in cardiomyocytes. Scale bars, 50 μm. **B** Quantification of ROS fluorescence intensity (*n* = 5 per group). **C** JC-1 polymer-to-monomer fluorescence ratio (*n* = 5 per group). **D** Representative immunoblot images and quantification of transferrin (TF), ferritin heavy chain 1 (FTH1), and GPX4 protein levels in cardiomyocytes (*n* = 6 per group). **E** Schematic illustration of the experimental design for NAC intervention in vivo. Eighteen-month-old rats were randomly assigned to receive control drinking water (Aging Control) or NAC-supplemented drinking water (Aging NAC) for 4 months. **F** Representative immunoblot images and quantification of transferrin (TF), ferritin heavy chain 1 (FTH1), and GPX4 protein levels in cardiac tissue from Aging Control and Aging NAC rats (*n* = 4 per group). **G** Representative M-mode echocardiographic images of the left ventricle from Aging Control and Aging NAC rats. Left ventricular ejection fraction (EF) (**H**) and fractional shortening (FS) (**I**) in Aging Control and Aging NAC rats (*n* = 4 per group). **J** Serum NT-proBNP levels in Aging Control and Aging NAC rats (*n* = 4 per group). **K** Representative hematoxylin and eosin (H&E)–stained sections of left ventricular tissue. Scale bars, 50 μm. **L** Representative Masson trichrome–stained sections of left ventricular tissue. Scale bars, 50 μm. **M** Quantification of left ventricular collagen volume fraction (*n* = 4 per group). The data are given as mean ± SEM and compared by Student’s t test.

To further determine whether HADHA deficiency promotes ferroptosis via depletion of intracellular glutathione (GSH), we supplemented HADHA-silenced cardiomyocytes with N-acetylcysteine (NAC), a well-established precursor of GSH. NAC treatment markedly attenuated reactive oxygen species (ROS) accumulation and restored mitochondrial membrane potential (MMP). Concomitantly, NAC reduced TF expression while increasing FTH1 and GPX4 expression (Fig. 5A–D). To assess whether NAC confers protection against ferroptosis in the context of cardiac aging in vivo, 18-month-old rats were treated with NAC for 4 months (Fig. 5E). Consistent with the cellular findings, NAC administration significantly decreased cardiac TF expression and increased FTH1 and GPX4 levels (Fig. 5F). Importantly, NAC treatment improved cardiac function, as evidenced by increased EF and FS, along with reduced serum NT-proBNP levels (Fig. 5G–J). Histological analyses further demonstrated that NAC alleviated age-associated myocardial structural disorganization and interstitial fibrosis (Fig. 5K–M). To further validate the therapeutic relevance of NAC in age-related heart failure, we employed a D-galactose–induced aging rat model, followed by treatment with either vehicle or NAC (Fig. S10A). NAC administration recapitulated the protective effects observed in naturally aged rats, including the downregulation of TF and the upregulation of FTH1 and GPX4 in cardiac tissue (Fig. S10B). Functionally, NAC significantly enhanced EF and FS (Fig. S10C–E), reduced NT-proBNP levels (Fig. S10F), and markedly ameliorated myocardial structural abnormalities and fibrosis (Fig. S10G–I). Collectively, these findings suggest that HADHA deficiency promotes cardiac ferroptosis through impairment of the GSH–GPX4 antioxidant system.

### SIRT1 deficiency downregulates HADHA expression through suppression of GATA4

**Fig. 6:**
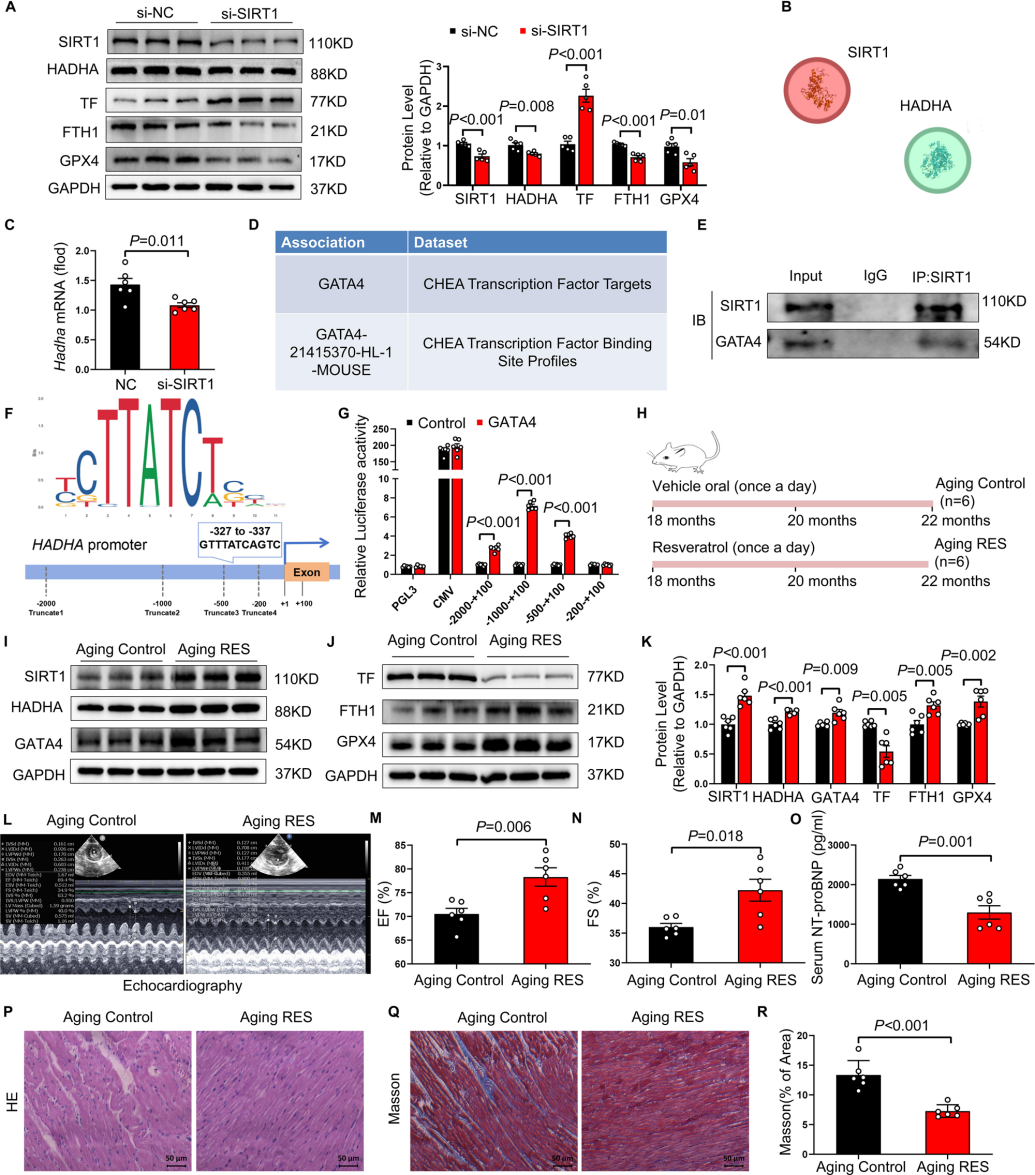
SIRT1 deficiency suppresses HADHA expression through inhibition of GATA4, whereas resveratrol supplementation preserves cardiac function in aging rats. **A** Neonatal rat cardiomyocytes were transfected with negative control siRNA (si-NC) or siRNA targeting SIRT1 (si-SIRT1). Representative immunoblots and quantitative analysis of SIRT1, HADHA, transferrin (TF), ferritin heavy chain 1 (FTH1), and glutathione peroxidase 4 (GPX4) protein levels are shown (*n* = 5 per group). **B** In silico molecular docking analysis illustrating the predicted interaction between SIRT1 and HADHA. **C** Relative mRNA expression of *Hadha* in cardiomyocytes following SIRT1 silencing, as determined by quantitative real-time PCR (*n* = 6 per group). **D** Bioinformatic enrichment analysis identifying GATA4 as a putative transcription factor associated with HADHA regulation, based on the ChEA Transcription Factor Targets and Binding Site Profiles datasets. **E** Co-immunoprecipitation analysis demonstrating the interaction between SIRT1 and GATA4 in cardiomyocytes. Cell lysates were immunoprecipitated with anti-SIRT1 antibody, followed by immunoblotting with antibodies against SIRT1 and GATA4. Representative immunoblots are shown. **F** Schematic illustration of the HADHA promoter region showing the predicted GATA4-binding motif (−327 to −337 bp) and the serial 5′-truncated promoter constructs generated for luciferase reporter assays. (**G**) Dual-luciferase reporter assays assessing the transcriptional activity of individual serially truncated HADHA promoter constructs following GATA4 overexpression or control treatment (*n* = 6 per group). **H** Experimental design for resveratrol intervention in aging rats. Eighteen-month-old rats were randomly assigned to receive vehicle (Aging Control) or resveratrol (Aging RES) by daily gavage for 4 months and were analyzed at 22 months of age. **I** Representative immunoblots of SIRT1, HADHA, and GATA4 protein expression in cardiac tissues from Aging Control and Aging RES rats. **J** Representative immunoblots of TF, FTH1, and GPX4 protein expression in cardiac tissues from Aging Control and Aging RES rats. **K** Quantitative analysis of SIRT1, HADHA, GATA4, TF, FTH1, and GPX4 protein levels in cardiac tissues from Aging Control and Aging RES rats (*n* = 6 per group). **L** Representative M-mode echocardiographic images from Aging Control and Aging RES rats. **M** Left ventricular ejection fraction (EF) in Aging Control and Aging RES rats (*n* = 6 per group). **N** Left ventricular fractional shortening (FS) in Aging Control and Aging RES rats (*n* = 6 per group). **O** Serum NT-proBNP levels in Aging Control and Aging RES rats (*n* = 6 per group). **P** Representative hematoxylin and eosin (H&E)–stained sections of left ventricular tissue. Scale bars, 50 μm. **Q** Representative Masson trichrome–stained sections of left ventricular tissue. Scale bars, 50 μm. **R** Quantification of left ventricular collagen volume fraction (*n* = 6 per group). The data are given as mean ± SEM and compared by Student’s t test.

Sirtuins are widely recognized as key regulators of anti-aging processes. Among them, SIRT1 has attracted particular interest as a potential therapeutic target for age-related diseases [24, 25]. Our previous study revealed a correlation between reduced SIRT1 and age-related atrial fibrillation in rats. However, whether SIRT1 is involved in age-related HF remains unclear. Intriguingly, silencing SIRT1 in cardiomyocytes resulted in a marked reduction in both SIRT1 and HADHA protein expression, accompanied by increased TF levels and decreased expression of GPX4 and FTH1 (Fig. 6A). Protein interaction prediction analysis revealed no direct binding between SIRT1 and HADHA. However, qPCR data showed a significant decrease in HADHA mRNA levels following SIRT1 silencing, suggesting that SIRT1 regulates HADHA expression at the transcriptional level. Database analysis identified GATA4 as a potential transcription factor regulating HADHA expression (Fig. 6B-D). Co-immunoprecipitation assays confirmed a direct interaction between SIRT1 and GATA4 in cardiomyocytes (Fig. 6E). Dual-luciferase reporter assays demonstrated that GATA4 significantly enhanced HADHA promoter activity, whereas truncation of the predicted GATA-binding region markedly attenuated this effect (Fig. 6F-G). ChIP–qPCR analyses further revealed significant enrichment of GATA4 at the HADHA promoter region containing the predicted GATA motifs compared with IgG controls (Fig. S11A). Importantly, GATA4 overexpression reversed the ferroptosis-related protein alterations induced by SIRT1 silencing, as evidenced by reduced TF expression and restoration of GPX4 and FTH1 levels (Fig. S11B). These findings suggest that SIRT1 deficiency downregulates GATA4, thereby inhibiting HADHA expression and promoting the occurrence of ferroptosis.

### Activation or overexpression of SIRT1 mitigates ferroptosis and protects aging rats from heart failure

To investigate the potential therapeutic effects of SIRT1 activation in age-related HF, we administered resveratrol, a known SIRT1 activator, to 18-month-old rats for 4 months (Fig. 6H). Compared with the control group, resveratrol treatment significantly increased the expression of SIRT1, HADHA, and GATA4 in cardiac tissues (Fig. 6I). Concurrently, resveratrol reduced TF expression and increased levels of FTH1 and GPX4 in the heart (Fig. 6J, K). Additionally, resveratrol treatment improved cardiac function **(**Fig. 6L–O) and reduced cardiac structural abnormalities and fibrosis (Fig. 6P–R). To determine whether restoration of SIRT1 is sufficient to improve age-related cardiac dysfunction, SIRT1 was selectively overexpressed in cardiomyocytes of D-galactose–induced aging mice via rAAV9-mediated gene delivery (Fig. S12A). SIRT1 overexpression significantly improved cardiac function, as indicated by increases in EF and FS, along with decreased serum NT-proBNP levels (Fig. S12B–D). Histological analyses further demonstrated preserved myocardial architecture and a substantial reduction in cardiac fibrosis (Fig. S12E–G). Furthermore, overexpression of SIRT1 led to increased expression of SIRT1, GATA4, and HADHA, reduced TF expression, and increased FTH1 and GPX4 (Fig. S12H–J). These findings suggest that SIRT1 may serve as a potential therapeutic target for aging-related HF.

## Discussion

This study provides new pathophysiological insights into the relationship between ferroptosis and age-related heart failure (HF). We demonstrate that ferroptosis represents an important mechanism contributing to age-related HF. In the hearts of aging rats, HADHA expression was markedly reduced, leading to mitochondrial dysfunction and excessive accumulation of reactive oxygen species, accompanied by depletion of glutathione (GSH) and downregulation of GPX4, thereby promoting ferroptosis. Furthermore, we found that SIRT1 deficiency contributes to the downregulation of HADHA in aging hearts through regulation of GATA4. Pharmacological activation or cardiomyocyte-specific overexpression of SIRT1 attenuated ferroptosis and protected aging hearts from HF. Collectively, these findings suggest that targeting HADHA or SIRT1 may represent a potential therapeutic strategy for age-related HF.

HF is a major public health burden, characterized by high morbidity and mortality. Its prevalence increases markedly with advancing age, and the pathophysiology of heart failure in the elderly remains complex and incompletely understood. In the present study, we observed a pronounced increase in ferroptosis in the cardiac tissues of aging rats. Ferroptosis is an iron-dependent form of regulated cell death characterized by lipid peroxidation. Accumulating evidence has linked ferroptosis with various age-related disorders [26, 27]. More recently, an increasing body of research has demonstrated the involvement of ferroptosis in the development and progression of various cardiovascular diseases, such as myocardial ischemia-reperfusion injury and cardiomyopathy [28, 29]. Accordingly, targeting ferroptosis has emerged as a potential therapeutic strategy for multiple disease contexts, including age-related disorders [10], cancer [30, 31], and cardiovascular diseases [32, 33]. In line with these observations, our findings revealed that a high-iron diet exacerbated ferroptosis in the cardiac tissues of aging rats, leading to significant impairment of cardiac function. Conversely, cardiomyocyte-specific GPX4 overexpression or pharmacological inhibition of ferroptosis effectively prevented cardiac dysfunction. Together, these results further support ferroptosis as a critical mechanism contributing to age-related HF.

HADHA is a catalytic subunit of the hydroxyacyl-CoA dehydrogenase trifunctional multienzyme complex, which is responsible for catalyzing the last three reactions of mitochondrial fatty acid β-oxidation [34]. HADHA plays an essential role in mitochondrial fatty acid oxidation and functions as an acyltransferase in cardiolipin remodeling, thereby contributing to cardiac homeostasis [35]. Impaired lipid oxidation can cause the accumulation of lipid peroxides, which represents an important mechanism underlying ferroptosis. We found that HADHA expression was significantly reduced in the hearts of aging rats and was further decreased in aging rats fed a high-iron diet. Deficiency of HADHA resulted in mitochondrial dysfunction and excessive generation of reactive oxygen species in cardiomyocytes. This accumulation of reactive oxygen species led to GSH depletion and suppression of GPX4, ultimately triggering ferroptosis. Moreover, cardiomyocyte-specific knockdown of HADHA exacerbated cardiac dysfunction in mice, whereas treatment with Fer-1, an inhibitor of ferroptosis, effectively rescued cardiac function, supporting the conclusion that HADHA deficiency promotes ferroptosis predominantly through reactive oxygen species accumulation and glutathione depletion.

SIRT1 is a highly conserved NAD^+^-dependent class III histone/protein deacetylase belonging to the sirtuin family, has been identified as an important regulator of aging [36]. Targeting SIRT1 has emerged as a novel therapeutic strategy for age-related disorders [37, 38]. Our previous study demonstrated that SIRT1 deficiency contributes to age-related atrial fibrillation through the regulation of necroptosis. Consistently, Berger reported the downregulation of SIRT1 in cellular senescence and aging [39]. In our present study, silencing of SIRT1 resulted in reduced expression of HADHA through direct binding to and downregulation of GATA4, a key transcription factor regulating HADHA expression. Furthermore, SIRT1 deficiency exacerbated ferroptosis in cardiomyocytes, whereas both pharmacological activation of SIRT1 with resveratrol and cardiomyocyte-specific overexpression of SIRT1 attenuated ferroptosis and conferred protection against cardiac dysfunction in aging rats. Notably, it has been reported that melatonin MT1 receptors prevent α-syn–induced ferroptosis in Parkinson’s disease through activation of the SIRT1/Nrf2/HO-1/GPX4 pathway [40], which is consistent with our findings and further supports the potential of SIRT1 activation as a strategy to suppress ferroptosis.

**Fig. 7:**
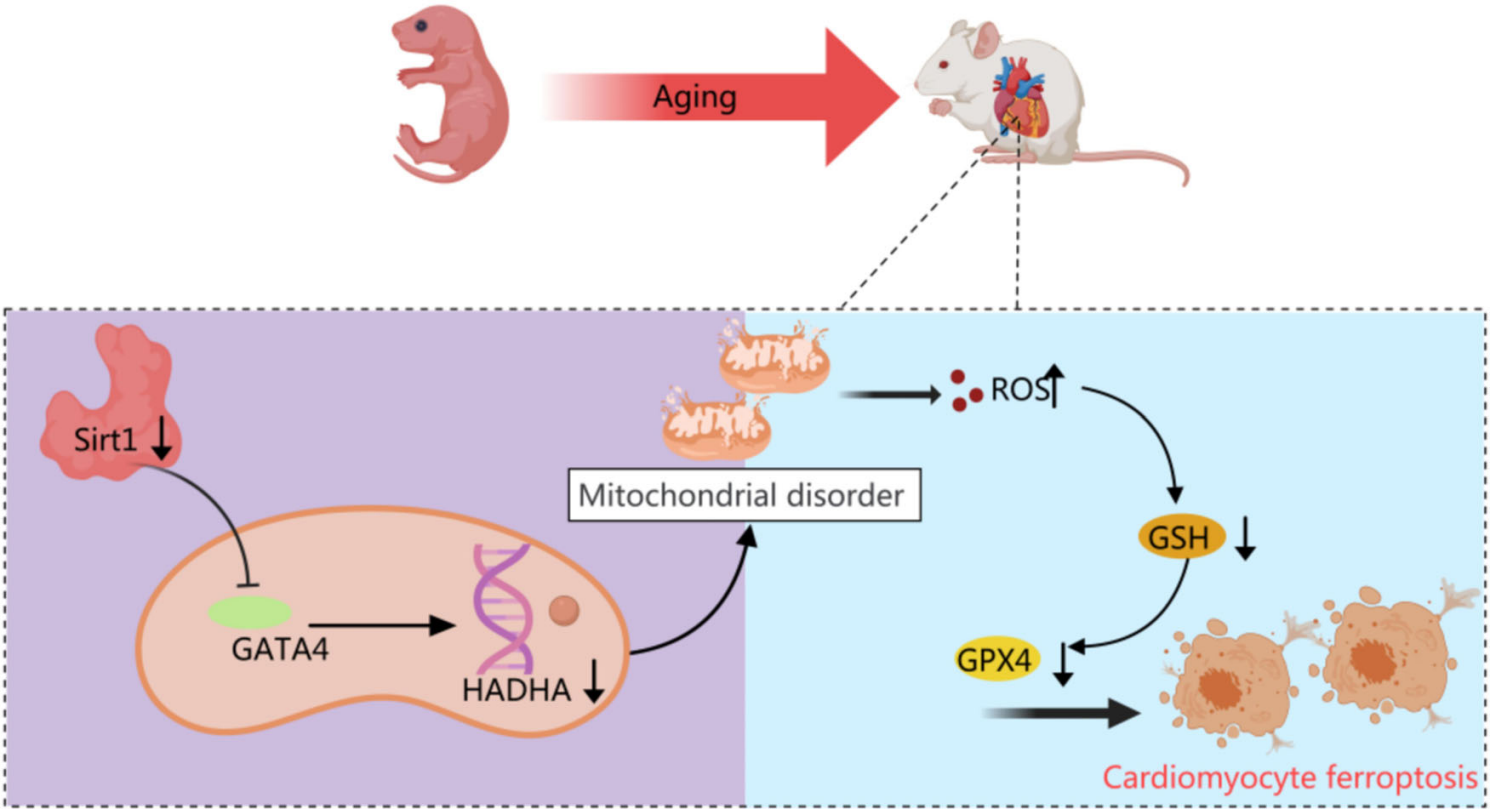
SIRT1 deficiency promotes age-related heart failure through enhancing ferroptosis via GATA4-HADHA-GPX4 axis. Aging is associated with reduced cardiac SIRT1 expression. SIRT1 deficiency attenuates GATA4 binding and transcriptional activation of the HADHA promoter, resulting in reduced HADHA expression. Downregulation of HADHA impairs mitochondrial function and increases reactive oxygen species (ROS) accumulation. Excessive ROS results in glutathione depletion and suppression of GPX4, thereby promoting lipid peroxidation and ferroptotic cell death in cardiomyocytes. Ferroptosis-driven myocardial injury ultimately contributes to the development of age-related heart failure.

In summary, our study provides novel mechanistic insights into the crucial role of ferroptosis in the progression of age-related HF. We demonstrated that aging rats exhibited increased accumulation of lipid peroxides and enhanced ferroptosis in the heart, while administration of a ferroptosis inhibitor effectively attenuated the decline of cardiac function in aging rats. Mechanistically, we found that age-related decline in SIRT1 led to downregulation of HADHA by binding to and suppressing its transcription factor GATA4. HADHA deficiency resulted in mitochondrial dysfunction and excessive accumulation of ROS, which in turn caused exhaustion of GSH and reduction of GPX4, thereby promoting ferroptosis and contributing to age-related HF. Collectively, these findings suggest that modulation of HADHA or SIRT1 represents a potential therapeutic strategy for the prevention and treatment of age-related HF (Fig. 7).

### Limitations

Several limitations of this study should be acknowledged. Firstly, due to the inherent challenges associated with obtaining human cardiac tissue, our findings regarding HADHA expression and the underlying mechanisms lack direct validation in human samples. Secondly, our conclusions regarding the protective effects of resveratrol against age-related HF are derived exclusively from animal models. Additional investigations, particularly well-designed clinical studies in human subjects, are warranted to determine the therapeutic potential of resveratrol in the context of age-related HF. Thirdly, although reductive aging models were employed in this study, the physiological relevance of our findings would be further strengthened by combining natural aging models with targeted genetic manipulation, such as cardiomyocyte-specific HADHA rescue in aged animals. Fourthly, our study primarily focused on HADHA deficiency–induced ferroptosis and did not systematically evaluate the contributions of other regulatory proteins or alternative forms of cell death that may also participate in the progression of age-related HF. Finally, the relatively small sample size inherent to natural aging models may limit statistical power and increase the risk of false-positive findings.

## Supplementary information

supplement figure

supplement table1-Targeted Analyte Information-UHPLC-QQQ-MS

supplement table2-Echocardiographic analysis of rats or mice

uncropped blots

## Data Availability

Data will be made available on request.
