# Supplementary material for: SIRT1 deficiency promotes age-related heart failure through enhancing ferroptosis via GATA4-HADHA-GPX4 axis
Source: Cell Death Dis. 2026 Mar 23;17(1):343. doi: 10.1038/s41419-026-08634-z (PMC13039550; doi:10.1038/s41419-026-08634-z)
Supplement: Supplementary file 1 — supplement figure [file 41419_2026_8634_MOESM1_ESM.docx]

**SIRT1 deficiency promotes age-related heart failure through enhancing ferroptosis via GATA4-HADHA-GPX4 axis**

Yu Duan, PhD^a,g^; Yingchun Luo, PhD^a,g^; Xuejie Han, PhD^a,g^; Hui Yu, PhD^b^; Hanwen Liu, MD^a^; Yun Zhou, PhD^a^; Yunlong Gao, PhD^b^; Qian Xu, PhD^c^; Ying Wei , PhD^b^; Ruoxin Min, MD^b^; Yong Hong, MD^b^; Xuanrui Ji, PhD^b^; Haibo Jia, PhD^d^; Yue Li, PhD^a,e,f*^; Yun Zhang, PhD^a*^

^a^Department of Cardiology, the First Affiliated Hospital, Harbin Medical University, Harbin 150001, China.

^b^NHC Key Laboratory of Cell Transplantation, The First Affiliated hospital of Harbin Medical University, Harbin, Heilongjiang Province, 150001, China.

^c^Anhui Provincial Center For Clinical Laboratories, The First Affiliated Hospital of USTC.

^d^Department of Cardiology, 2nd Affiliated Hospital of Harbin Medical University, Harbin, 150001, PR China

^e^Key Laboratory of Cardiac Diseases and Heart Failure, Harbin Medical University, Harbin 150001, China.

^f^State Key Laboratory of Frigid Zone Cardiovascular Disease, Harbin Medical University, Harbin, Heilongjiang 150086, China.

^g^These authors contributed equally.

*Corresponding auth：[zhangyun263727@163.com](mailto:ly99ly@vip.163.com) (Yun Zhang), [ly99ly@vip.163.com](mailto:ly99ly@vip.163.com) (Yue Li).


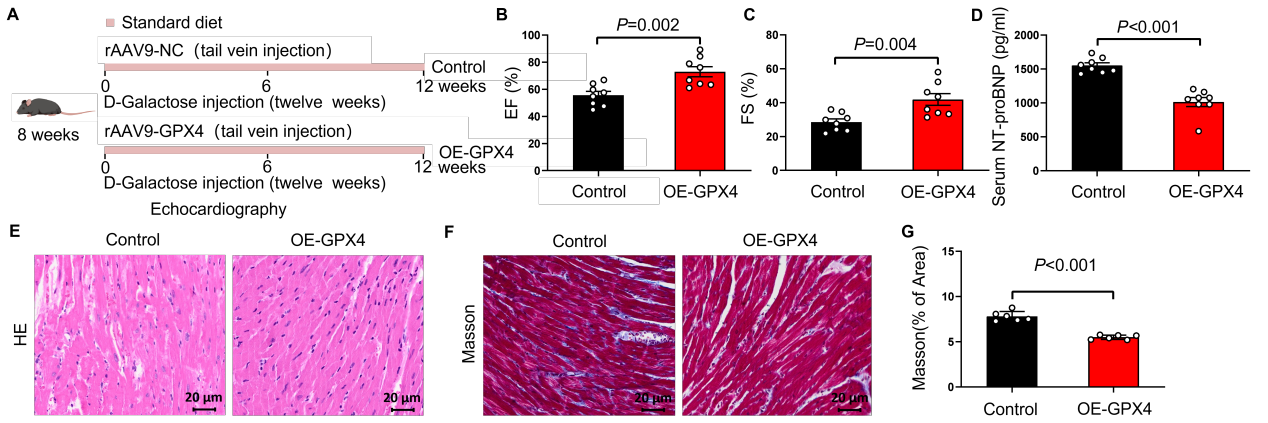


**Figure S1. Cardiomyocyte-specific overexpression of GPX4 improves cardiac function in D-galactose–induced aging mice.**

**(A)** Experimental design. Eight-week-old mice received tail vein injection of rAAV9 encoding cardiomyocyte-specific GPX4 (OE-GPX4) or control vector (rAAV9-NC). Both groups were subjected to D-galactose administration by daily subcutaneous injection for 12 weeks to induce an aging phenotype. Cardiac function was assessed by echocardiography at the end of the treatment period.

**(B–C)** Left ventricular ejection fraction (EF) (**B**) and fractional shortening (FS) (**C**) measured by echocardiography (n = 8 per group).

**(D)** Serum N-terminal pro–B-type natriuretic peptide (NT-proBNP) levels in mice from Control group and OE-GPX4 group (n = 8 per group).

**(E)** Representative hematoxylin and eosin (H&E)–stained sections of left ventricular myocardium of mice from Control group and OE-GPX4 group. Scale bars, 20 μm.

**(F)** Representative Masson trichrome–stained sections showing myocardial fibrosis of mice from Control group and OE-GPX4 group. Scale bars, 20 μm.

**(G)** Quantification of left ventricular collagen volume fraction based on Masson staining in mice (n = 6 per group).

The data are given as mean ± SEM and compared by Student’s t test.


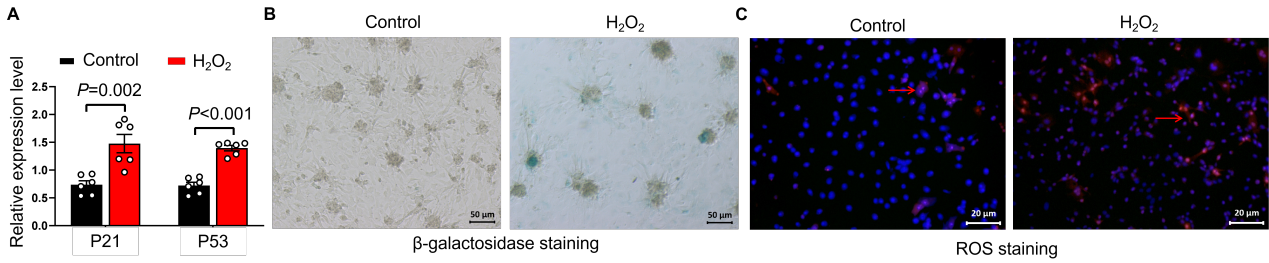


**Figure S2. Hydrogen peroxide induces senescence and oxidative stress in cardiomyocytes.**

Cardiomyocytes were treated with H_2_O_2_ for 24 hours to establish senescent cardiomyocytes model.

1. Relative mRNA expression levels of the senescence markers p21 and p53 in cardiomyocytes of Control group and H_2_O_2_ group (n=6 per group).
2. Representative images of β-galactosidase staining in cardiomyocytes of Control group and H_2_O_2_ group. Scale bars, 50 μm.
3. Representative images of ROS staining in cardiomyocytes of Control group and H_2_O_2_ group. Scale bars, 20 μm.

The data are given as mean ± SEM and compared by Student’s t test.


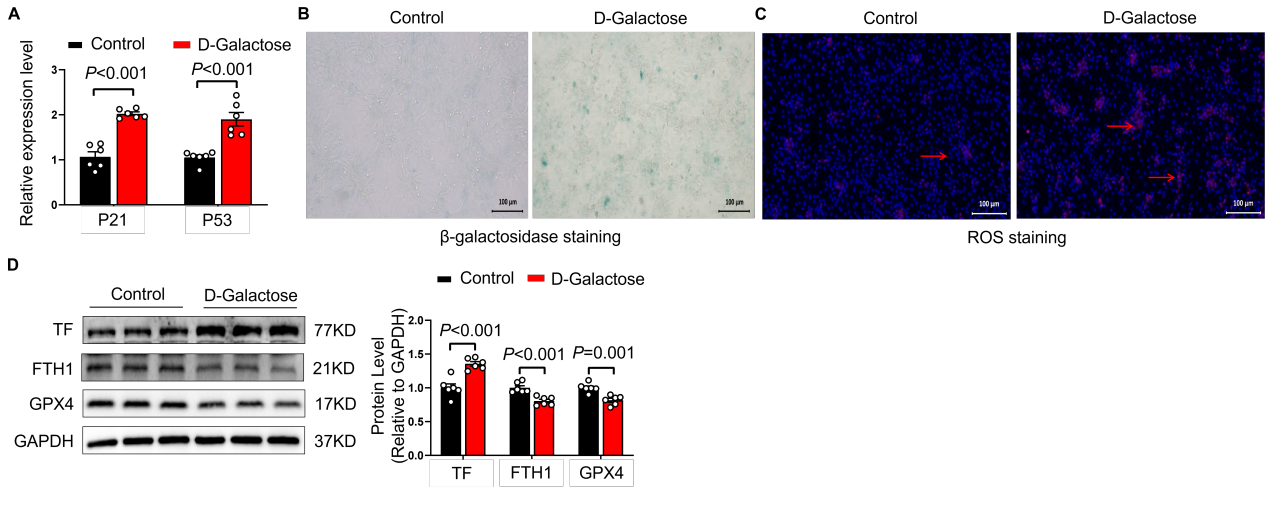


**Figure S3. D-Galactose induces cellular senescence and oxidative stress in cardiomyocytes.**

(**A)** Relative mRNA expression levels of the senescence markers p21 and p53 in cardiomyocytes from Control and D-galactose–treated groups (n = 6 per group).

(**B)** Representative images of senescence-associated β-galactosidase (SA–β-gal) staining in cardiomyocytes from Control and D-galactose–treated groups. Scale bars, 100 μm.

(**C)** Representative images of reactive oxygen species (ROS) staining in cardiomyocytes from Control and D-galactose–treated groups. Arrows indicate ROS-positive cells. Scale bars, 100 μm.

(**D)** Representative immunoblots and quantitative analysis of transferrin (TF), ferritin heavy chain 1 (FTH1), and glutathione peroxidase 4 (GPX4) protein expression in cardiomyocytes. Protein levels were normalized to GAPDH (n = 6 per group).

The data are given as mean ± SEM and compared by Student’s t test.





**Figure S4. HID diet did not exert influence on ferroptosis and cardiac function of young rats.**

**(A)** Schematic illustration of the experimental design for ND or HID diet in young rats. 2 months old rats were randomly divided into two groups, receiving a standard diet(ND) or a high-iron diet (HID) for 4 months.

**(B–C)** Left ventricular ejection fraction (EF) (**B**) and fractional shortening (FS) (**C**) measured by echocardiography in rats (n = 6 per group).

**(D)** Serum NT-proBNP levels in rats from Young ND and Young HID group (n = 6 per group).

**(E)** Representative image of Perls’ Blue staining in the left ventricle of rats from Young ND and Young HID group. Scale bars, 250 μm.

**(F)** Representative immunoblots and quantitative analysis of transferrin (TF), ferritin heavy chain 1 (FTH1), and glutathione peroxidase 4 (GPX4) protein expression in rats from Young ND group and Young HID group (n=6 per group).

The data are given as mean ± SEM and compared by Student’s t test.

**
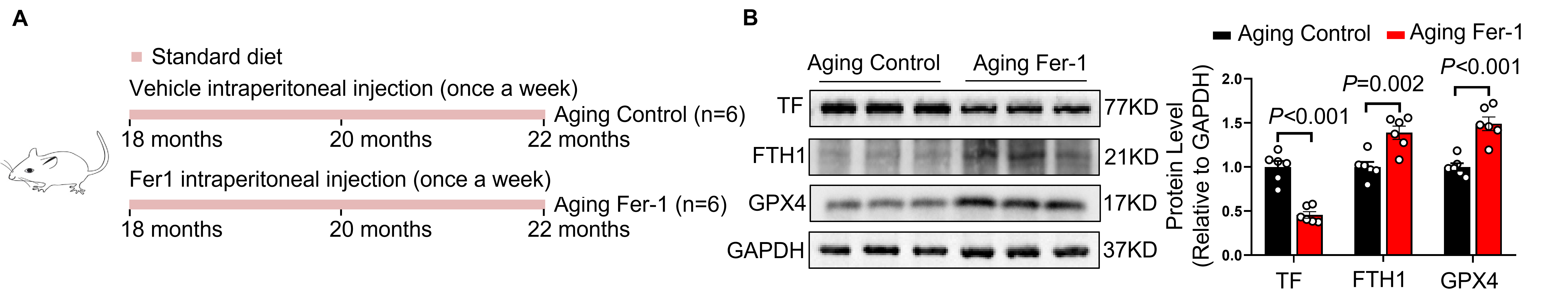
**

**Figure-S5. Ferroptosis inhibitor protected aging rats from ferroptosis in ventricle.**

**(A)** Schematic illustration of the experimental design for ferrostatin-1intervention. 18 months old rats were randomly divided into two groups, receiving control solvent or ferrostatin-1 through intraperitoneal injection, once a week, for 4 months.

**(B)** Representative immunoblots and quantitative analysis of transferrin (TF), ferritin heavy chain 1 (FTH1), and glutathione peroxidase 4 (GPX4) protein expression in rats from Aging Control group and Aging Fer-1 group (n=6 per group).

The data are given as mean ± SEM and compared by Student’s t test.


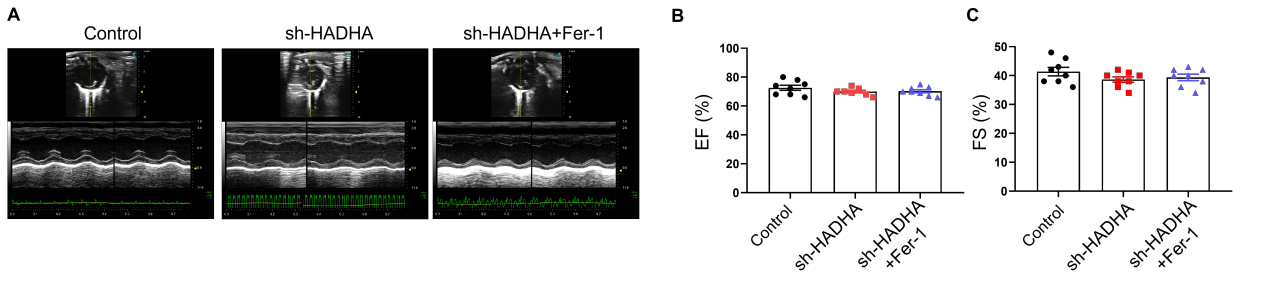


**Figure S6. Effects of cardiomyocyte-specific HADHA knockdown on cardiac function in mice**

**(A)** Representative M-mode echocardiographic images of the left ventricle in mice.

**(B-C)** Left ventricular ejection fraction (EF) (**B**) and fractional shortening (FS) (**C**) in mice from each group (n=8 per group).


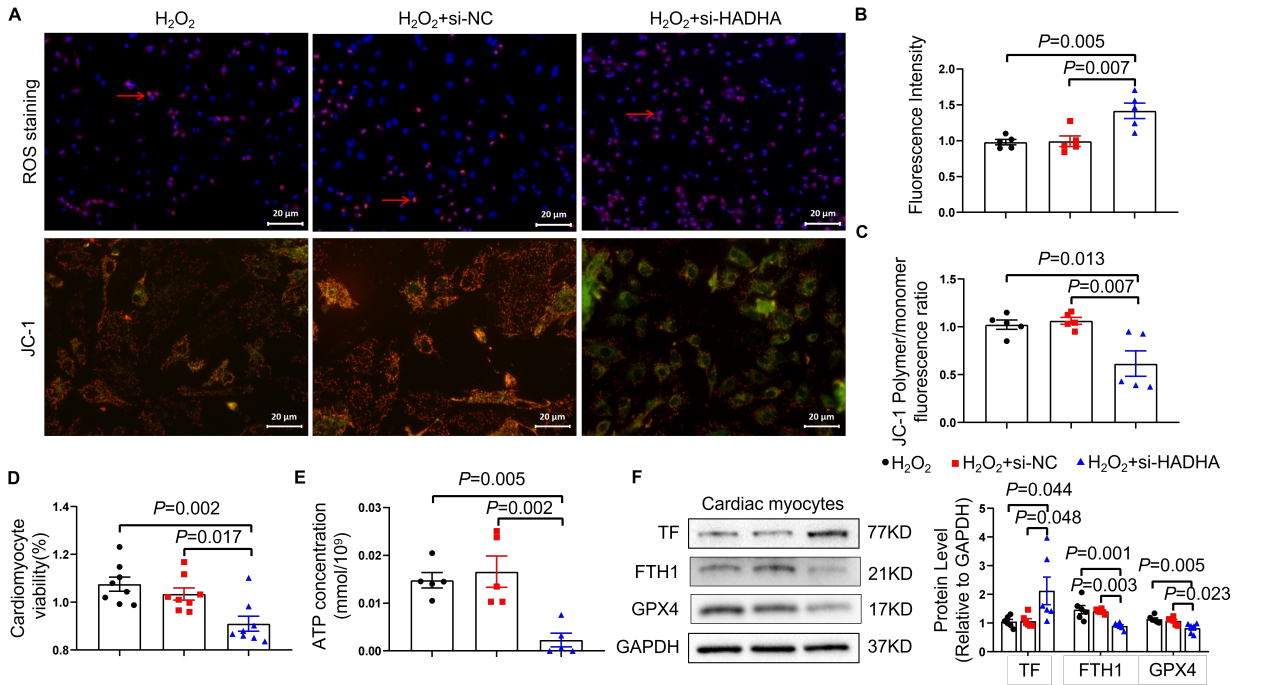


**Figure S7. Silencing of HADHA increases reactive oxygen species and induces mitochondrial dysfunction in cardiomyocytes.**

Cardiomyocytes were transfected with siRNA targeting HADHA or a negative control. After 24 hours, the culture medium was replaced, and cells were treated with H₂O₂ for an additional 24 hours prior to downstream analyses.

**(A)** Representative images of reactive oxygen species (ROS) staining and JC-1 staining in cardiomyocytes. Scale bars, 20 μm.

**(B)** Quantification of ROS fluorescence intensity in cardiomyocytes (n = 5 per group).

**(C)** JC-1 polymer-to-monomer fluorescence ratio in cardiomyocytes (n = 5 per group).

**(D)** Cardiomyocyte viability (n = 8 per group).

**(E)** Intracellular ATP levels in cardiomyocytes (n = 5 per group).

**(F)** Representative immunoblots and quantitative analysis of transferrin (TF), ferritin heavy chain 1 (FTH1), and glutathione peroxidase 4 (GPX4) protein expression in cardiomyocytes (n = 6 per group).

Data are presented as mean ± SEM and were analyzed by one-way ANOVA.


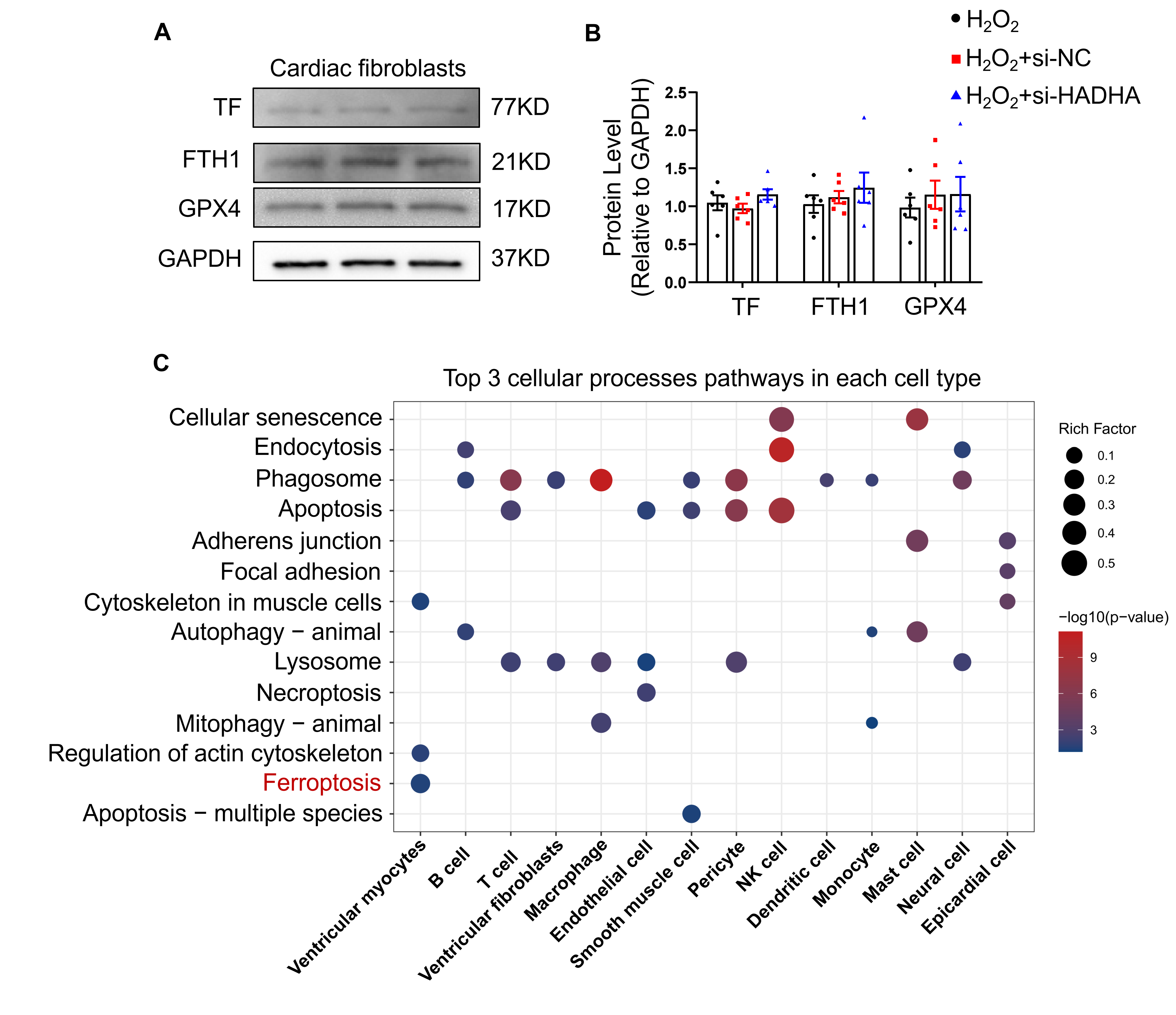


**Figure S8. Silencing of HADHA does not affect ferroptosis-related pathways in cardiac fibroblasts.**

Cardiac fibroblasts were transfected with siRNA to silence HADHA. After 24 hours, following a change of culture media, the cells were treated with H_2_O_2_ for 24 hours before conducting assays for relevant markers.

**(A)** Representative immunoblots showing the protein expression of transferrin (TF), ferritin heavy chain 1 (FTH1), and glutathione peroxidase 4 (GPX4) in cardiac fibroblasts.

**(B)** Quantitative analysis of TF, FTH1, and GPX4 protein expression in cardiac fibroblasts, normalized to GAPDH (n = 6 per group).

**(C)** Dot plot showing the top enriched cellular process pathways across major cardiac cell types identified by single-cell RNA sequencing analysis of human heart tissues from young and aged individuals. Ferroptosis-related pathways are highlighted.

The data are given as mean ± SEM and compared by one way ANOVA.


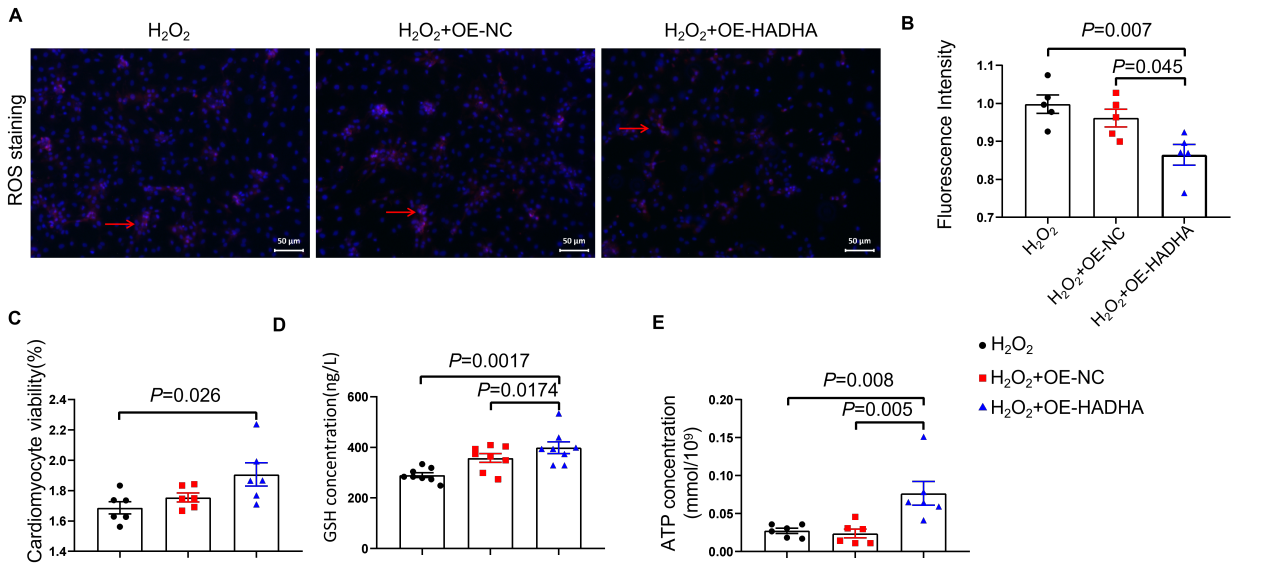


**Figure S9. Overexpression of HADHA leads to an decrease in reactive oxygen species and alleviates mitochondrial damage in** **cardiomyocytes.**

Cardiomyocytes were first transfected with plasmids for overexpression of HADHA. After 24 hours, following a change of culture media, the cells were treated with H_2_O_2_ for 24 hours before conducting assays for relevant markers.

**(A)** Representative images of ROS staining in cardiomyocytes.Scale bars, 50 μm.

**(B)** The fluorescence intensity of ROS staining of cardiomyocytes (n=5 per group).

**(C)** The viability of cardiomyocytes (n=6 per group).

**(D)** The levels of GSH in cardiomyocytes (n=8 per group).

**(E)** The levels of ATP in cardiomyocytes (n=6 per group).

The data are given as mean ± SEM and compared by one way ANOVA.


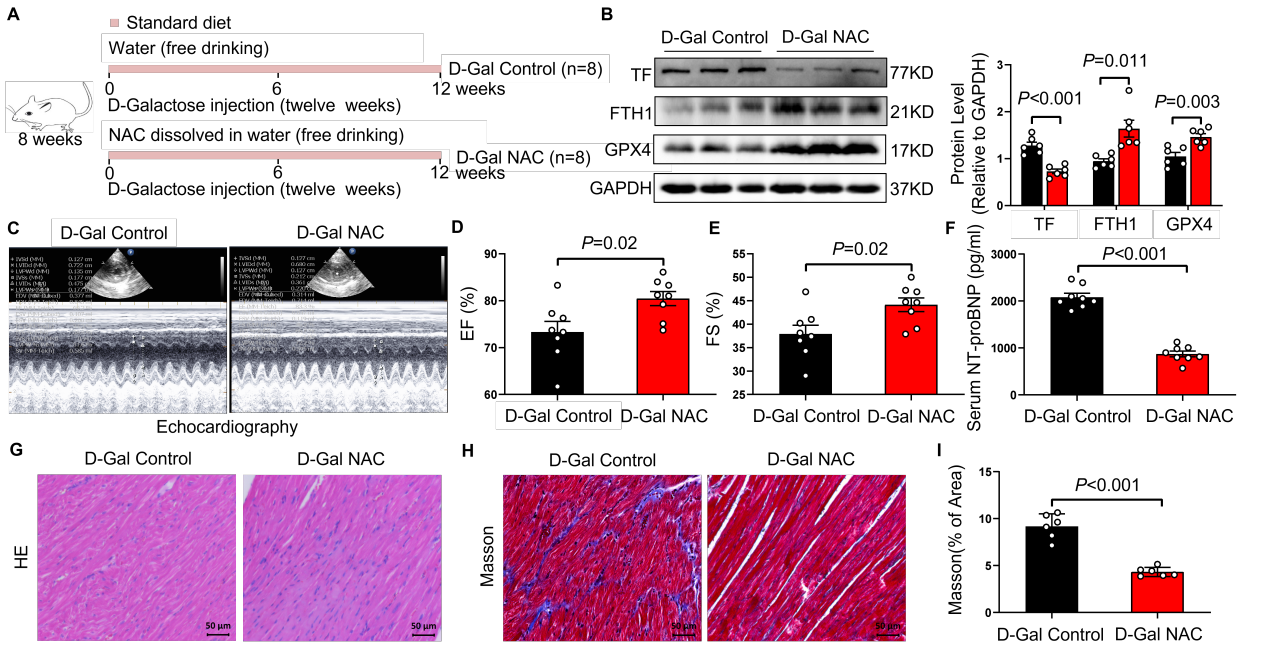


**Figure S10. Supplementation of NAC protects D-galactose-induced aging rats from heart failure.**

**(A)** Schematic illustration of the experimental design. We established a aging rat model with D-galactose via subcutaneous injection. Then rats were randomly divided into two groups, receiving control solvent (D-Gal Control group) or NAC (D-Gal NAC group) through drinking for 12 weeks.

**(B)** Representative immunoblot bands and quantification of the expression of TF, FTH1 and GPX4 in the heart of rats from D-Gal Control group and D-Gal NAC group (n=6 per group).

**(C)** Representative M-mode images of heart in from D-Gal Control group and D-Gal NAC group.

**(D)** The statistical data of left ventricular ejection fraction (EF) of rats from D-Gal Control group and D-Gal NAC group (n=8 per group).

**(E)** The statistical data of left ventricular fraction shortening (FS) of rats from D-Gal Control group and D-Gal NAC group (n=8 per group).

**(F)** The levels of plasma NT-ProBNP in rats from D-Gal Control group and D-Gal NAC group (n=8 per group).

**(G)** Representative images of HE staining of the left ventricle of rats from D-Gal Control group and D-Gal NAC group.Scale bars, 50 μm.

**(H)** Representative images of Masson staining of the left ventricle of rats from D-Gal Control group and D-Gal NAC group. Scale bars, 50 μm.

**(I)** The collagen volume fraction of the left ventricle of rats from D-Gal Control group and D-Gal NAC group (n=6 per group).

The data are given as mean ± SEM and compared by Student’s t test.


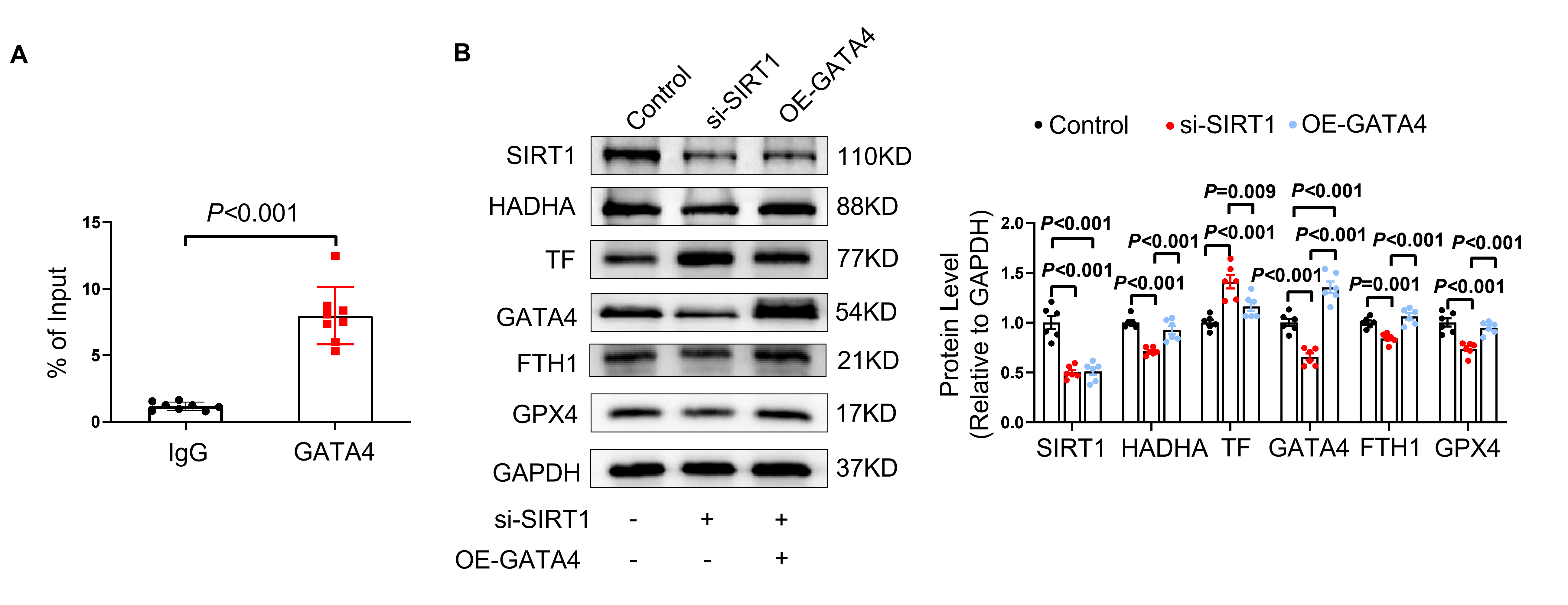


**Figure S11. GATA4 directly regulates HADHA transcription and rescues SIRT1 deficiency–induced ferroptosis in neonatal rat cardiomyocytes.**

**(A)** ChIP–qPCR analysis showing the enrichment of GATA4 on the HADHA promoter (n = 8 per group). IgG served as a negative control.

**(B)** Representative immunoblots and quantitative analysis of SIRT1, HADHA, GATA4, TF, FTH1, and GPX4 in primary neonatal rat cardiomyocytes from control group, si-SIRT1 group, or OE-GATA4 group (combined SIRT1 knockdown with GATA4 overexpression).

The data are given as mean ± SEM and compared by one way ANOVA.


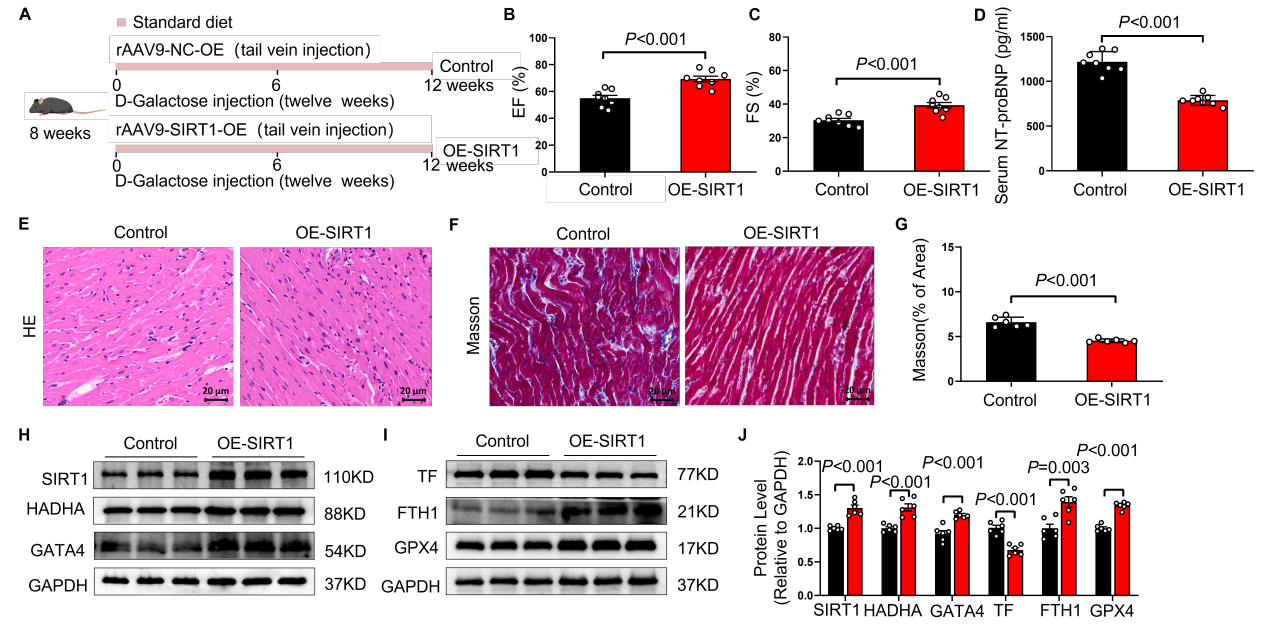


**Figure S12. Cardiomyocyte-specific overexpression of SIRT1 improves cardiac function in D-galactose–induced aging mice.**

**(A)** Schematic illustration of the experimental design. Eight-week-old mice were injected via the tail vein with rAAV9 carrying a cardiomyocyte-specific SIRT1 overexpression construct (OE-SIRT1) or a control vector (rAAV9-NC). Both groups subsequently received daily subcutaneous injections of D-galactose for 12 weeks to induce an aging phenotype. Cardiac structure and function were evaluated by echocardiography at the end of the treatment period.

(**B–C**) Echocardiographic assessment of left ventricular ejection fraction (EF) and fractional shortening (FS) in Control and OE-SIRT1 mice (n = 8 per group).

**(D)** Serum NT-proBNP levels in Control and OE-SIRT1 mice (n = 8 per group).

**(E)** Representative hematoxylin and eosin (H&E)–stained sections of left ventricular myocardium from Control and OE-SIRT1 mice. Scale bars, 20 μm.

**(F)** Representative Masson trichrome–stained sections showing myocardial fibrosis in Control and OE-SIRT1 mice. Scale bars, 20 μm.

**(G)** Quantification of myocardial fibrosis, expressed as collagen volume fraction based on Masson staining (n = 6 per group).

**(H)** Representative immunoblot bands for SIRT1, HADHA, and GATA4 in left ventricular tissue from Control and OE-SIRT1 mice.

**(I)** Representative immunoblot bands for TF, FTH1, and GPX4 in left ventricular tissue from Control and OE-SIRT1 mice.

**(J)** Quantitative analysis of SIRT1, HADHA, GATA4, TF, FTH1, and GPX4 protein expression in left ventricular tissue from Control and OE-SIRT1 mice (n = 6 per group).

The data are given as mean ± SEM and compared by Student’s t test.
