# Supplementary material for: SIRT1 deficiency promotes age-related heart failure through enhancing ferroptosis via GATA4-HADHA-GPX4 axis
Source: Cell Death Dis. 2026 Mar 23;17(1):343. doi: 10.1038/s41419-026-08634-z (PMC13039550; doi:10.1038/s41419-026-08634-z)
Supplement: Supplementary file 2 — supplement table1-Targeted Analyte Information-UHPLC-QQQ-MS [file 41419_2026_8634_MOESM2_ESM.docx]

**Supplemental Table 1-Targeted Analyte Information-UHPLC-QQQ-MS**

| **Compound Name** | **Cpd Name** | **Abbr.** | **CAS** | **Formula** | **M.W.** |
| --- | --- | --- | --- | --- | --- |
| ±11(12)-EET | (±)11,(12)-epoxy-5Z,8Z,14Z-eicosatrienoic acid | ±11(12)-EET | 123931-40-8 | C20H32O3 | 320.5 |
| ±11,12-DiHETrE | (±)11,12-dihydroxy-5Z,8Z,14Z-eicosatrienoic acid | ±11,12-DiHETrE | / | C20H34O4 | 338.5 |
| ±11-HDoHE | (±)11-hydroxy-4Z,7Z,9E,13Z,16Z,19Z-docosahexaenoic acid | ±11-HDoHE | 87018-59-5 | C22H32O3 | 344.5 |
| ±11-HEPE | (±)-11-hydroxy-5Z,8Z,12E,14Z,17Z-eicosapentaenoic acid | ±11-HEPE | 99217-78-4 | C20H30O3 | 318.5 |
| ±12(13)-DiHOME | (±)12,13-dihydroxy-9Z-octadecenoic acid | ±12(13)-DiHOME | 263399-35-5 | C18H34O4 | 314.5 |
| ±12(13)-EpOME | (±)12(13)epoxy-9Z-octadecenoic acid | ±12(13)-EpOME | / | C18H32O3 | 296.5 |
| ±13-HDoHE | (±)13-hydroxy-4Z,7Z,10Z,14E,16Z,19Z-docosahexaenoic acid | ±13-HDoHE | 90780-53-3 | C22H32O3 | 344.5 |
| ±14(15)-EET | (±)14(15)-epoxy-5Z,8Z,11Z-eicosatrienoic acid | ±14(15)-EET | 81276-03-1 | C20H32O3 | 320.5 |
| ±14(15)-EpETE | (±)14,15-epoxy-5Z,8Z,11Z,17Z-eicosatetraenoic acid | ±14(15)-EpETE | 131339-24-7 | C20H30O3 | 318.5 |
| ±14,15-DiHETrE | (±)14,15-dihydroxy-5Z,8Z,11Z-eicosatrienoic acid | ±14,15-DiHETrE | / | C20H34O4 | 338.5 |
| ±16,17-EpDPE | (±)16,17-epoxy-4Z,7Z,10Z,13Z,19Z-docosapentaenoic acid | ±16,17-EpDPE | 155073-46-4 | C22H32O3 | 344.5 |
| ±17(18)-EpETE | (±)17,18-epoxy-5Z,8Z,11Z,14Z-eicosatetraenoic acid | ±17(18)-EpETE | / | C20H30O3 | 318.5 |
| ±18-HETE | (±)18-hydroxy-5Z,8Z,11Z,14Z-eicosatetraenoic acid | ±18-HETE | 133268-58-3 | C20H32O3 | 320.5 |
| ±19(20)-DiHDPA | (±)19,20-dihydroxy-4Z,7Z,10Z,13Z,16Z-docosapentaenoic acid | ±19(20)-DiHDPA | / | C22H34O4 | 362.5 |
| ±19,20-EpDPE | (±)19,20-epoxy-4Z,7Z,10Z,13Z,16Z-docosapentaenoic acid | ±19,20-EpDPE | / | C22H32O3 | 344.5 |
| ±20-HDoHE | (±)20-hydroxy-4Z,7Z,10Z,13Z,16Z,18E-docosahexaenoic acid | ±20-HDoHE | 90906-41-5 | C22H32O3 | 344.5 |
| ±5,6-DiHETrE | (±)5,6-dihydroxy-8Z,11Z,14Z-eicosatrienoic acid | ±5,6-DiHETrE | 213382-49-1 | C20H34O4 | 338.5 |
| ±5-iso PGF2α-VI | (8β)-5,9α,11α-trihydroxy-prosta-6E,14Z-dien-1-oic acid | ±5-iso PGF2α-VI | 179094-11-2 | C20H34O5 | 354.5 |
| ±8(9)-EET | (±)8,9-epoxy-5Z,11Z,14Z-eicosatrienoic acid | ±8(9)-EET | / | C20H32O3 | 320.5 |
| ±8,9-DiHETrE | (±)8,9-dihydroxy-5Z,11Z,14Z-eicosatrienoic acid | ±8,9-DiHETrE | 192461-96-4 | C20H34O4 | 338.5 |
| ±8-HDoHE | (±)8-hydroxy-4Z,6E,10Z,13Z,16Z,19Z-docosahexaenoic acid | ±8-HDoHE | 90780-54-4 | C22H32O3 | 344.5 |
| ±9(10)-DiHOME | (±)9,10-dihydroxy-12Z-octadecenoic acid | ±9(10)-DiHOME | 263399-34-4 | C18H34O4 | 314.5 |
| ±9(10)-EpOME | (±)9,10-epoxy-12Z-octadecenoic acid | ±9(10)-EpOME | 65167-83-1 | C18H32O3 | 296.5 |
| 10-Nitrooleic Acid | 10-nitro-9E-octadecenoic acid | 10-Nitrooleic Acid | 875685-46-4 | C18H33NO4 | 327.5 |
| 11-dehydro TXB2 | 9α,15S-dihydroxy-11-oxothromba-5Z,13E-dien-1-oic acid | 11-dehydro TXB2 | 67910-12-7 | C20H32O6 | 368.5 |
| 11S-HETE | 11S-hydroxy-5Z,8Z,12E,14Z-eicosatetraenoic acid | 11S-HETE | 54886-50-9 | C20H32O3 | 320.5 |
| 11β-13,14-dihydro-15-keto PGF2α | 9α,11β-dihydroxy-15-oxo-prost-5Z-en-1-oic acid | 11β-13,14-dihydro-15-keto PGF2α | 107615-77-0 | C20H34O5 | 354.5 |
| 11β-PGE2 | 9-oxo-11β,15S-dihydroxy-prosta-5Z,13E-dien-1-oic acid | 11β-PGE2 | 38310-90-6 | C20H32O5 | 352.5 |
| 11β-PGF2α | 9α,11β,15S-trihydroxy-prosta-5Z,13E-dien-1-oic acid | 11β-PGF2α | 38432-87-0 | C20H34O5 | 354.5 |
| 12-oxo LTB4 | 5S-hydroxy-12-oxo-6Z,8E,10E,14Z-eicosatetraenoic acid | 12-oxo LTB4 | 136696-10-1 | C20H30O4 | 334.4 |
| 12-OxoETE | 12-oxo-5Z,8Z,10E,14Z-eicosatetraenoic acid | 12-OxoETE | 108437-64-5 | C20H30O3 | 318.5 |
| 12S-HEPE | 12S-hydroxy-5Z,8Z,10E,14Z,17Z-eicosapentaenoic acid | 12S-HEPE | 116180-17-7 | C20H30O3 | 318.5 |
| 12S-HETE | 12S-hydroxy-5Z,8Z,10E,14Z-eicosatetraenoic acid | 12S-HETE | 54397-83-0 | C20H32O3 | 320.5 |
| 12S-HHTrE | 12S-hydroxy-5Z,8E,10E-heptadecatrienoic acid | 12S-HHTrE | 54397-84-1 | C17H28O3 | 280.4 |
| 13,14-dihydro PGF2α | 9α,11α,15S-trihydroxy-prost-5Z-en-1-oic acid | 13,14-dihydro PGF2α | 27376-74-5 | C20H36O5 | 356.5 |
| 13,14-dihydro-15-keto PGD2 | 9α-hydroxy-11,15-dioxo-prost-5Z-en-1-oic acid | 13,14-dihydro-15-keto PGD2 | 59894-07-4 | C20H32O5 | 352.5 |
| 13,14-dihydro-15-keto PGF2α | 9α,11α-dihydroxy-15-oxo-prost-5Z-en-1-oic acid | 13,14-dihydro-15-keto PGF2α | 27376-76-7 | C20H34O5 | 354.5 |
| 13-OxoODE | 13-oxo-9Z,11E-octadecadienoic acid | 13-OxoODE | 54739-30-9 | C18H30O3 | 294.4 |
| 13S-HODE | 13S-hydroxy-9Z,11E-octadecadienoic acid | 13S-HODE | 29623-28-7 | C18H32O3 | 296.5 |
| 13S-HOTrE | 13S-hydroxy-9Z,11E,15Z-octadecatrienoic acid | 13S-HOTrE | 87984-82-5 | C18H30O3 | 294.4 |
| 13S-HOTrE(γ) | 13S-hydroxy-6Z,9Z,11E-octadecatrienoic acid | 13S(γ)-HOTrE | 74784-20-6 | C18H30O3 | 294.4 |
| 14,15-LTC4 | 15S-hydroxy-14R-(S-glutathionyl)-5Z,8Z,10E,12E-eicosatetraenoic acid | 14,15-LTC4 | 75290-60-7 | C30H47N3O9S | 625.8 |
| 14,15-LTD4 | S-[(1R,2E,4E,6Z,9Z)-13-carboxy-1-[(1S)-1-hydroxyhexyl]-2,4,6,9-tridecatetraen-1-yl]-L-cysteinyl-glycine | 14,15-LTD4 | 75290-64-1 | C25H40N2O6S | 496.7 |
| 14,15-LTE4 | 15S-hydroxy-14R-(S-cysteinyl)-5Z,8Z,10E,12E-eicosatetraenoic acid | 14,15-LTE4 | 1000852-57-2 | C23H37NO5S | 439.6 |
| 15-deoxy-Δ12,14-PGD2 | 9α-hydroxy-11-oxo-prosta-5Z,12E,14E-trien-1-oic acid | 15-deoxy-Δ12,14-PGD2 | 85235-11-6 | C20H30O4 | 334.4 |
| 15-deoxy-Δ12,14-PGJ2 | 11-oxo-prosta-5Z,9,12E,14E-tetraen-1-oic acid | 15-deoxy-Δ12,14-PGJ2 | 87893-55-8 | C20H28O3 | 316.4 |
| 15-keto PGE2 | 9,15-dioxo-11α-hydroxy-prosta-5Z,13E-dien-1-oic acid | 15-keto PGE2 | 26441-05-4 | C20H30O5 | 350.5 |
| 15-keto PGF1α | 9α,11α-dihydroxy-15-oxo-prost-13E-en-1-oic acid | 15-keto PGF1α | 21562-58-3 | C20H34O5 | 354.5 |
| 15-OxoEDE | 15-oxo-11Z,13E-eicosadienoic acid | 15-OxoEDE | 105835-44-7 | C20H34O3 | 322.5 |
| 15-OxoETE | 15-oxo-5Z,8Z,11Z,13E-eicosatetraenoic acid | 15-OxoETE | 81416-72-0 | C20H30O3 | 318.5 |
| 15S-HEPE | 15S-hydroxy-5Z,8Z,11Z,13E,17Z-eicosapentaenoic acid | 15S-HEPE | 86282-92-0 | C20H30O3 | 318.5 |
| 15S-HETrE | 15S-hydroxy-8Z,11Z,13E-eicosatrienoic acid | 15S-HETrE | 92693-02-2 | C20H34O3 | 322.5 |
| 16S-HETE | 16S-hydroxy-5Z,8Z,11Z,14Z-eicosatetraenoic acid | 16S-HETE | 183509-23-1 | C20H32O3 | 320.5 |
| 17S-HETE | 17S-hydroxy-5Z,8Z,11Z,14Z-eicosatetraenoic acid | 17S-HETE | 183509-25-3 | C20H32O3 | 320.5 |
| 19R-hydroxy PGE2 | 9-oxo-11α,15S,19R-trihydroxy-prosta-5Z,13E-dien-1-oic acid | 19R-hydroxy PGE2 | 64625-54-3 | C20H32O6 | 368.5 |
| 19R-hydroxy PGF2α | 9α,11α,15S,19R-tetrahydroxy-prosta-5Z,13E-dien-1-oic acid | 19R-hydroxy PGF2α | 64625-53-2 | C20H34O6 | 370.5 |
| 19S-HETE | 19S-hydroxy-5Z,8Z,11Z,14Z-eicosatetraenoic acid | 19S-HETE | 115461-40-0 | C20H32O3 | 320.5 |
| 1a,1b-dihomo PGE2 | 9-oxo-11α,15S-dihydroxy-1a,1b-dihomo-prosta-5Z,13E-dien-1-oic acid | 1a,1b-dihomo PGE2 | 26198-80-1 | C22H36O5 | 380.5 |
| 1a,1b-dihomo PGF2α | 9α,11α,15S-trihydroxy-1a,1b-dihomo-prosta-5Z,13E-dien-1-oic acid | 1a,1b-dihomo PGF2α | 57944-39-5 | C22H38O5 | 382.5 |
| 2,3-dinor TXB2 | 9α,11,15S-trihydroxy-2,3-dinor-thromba-5Z,13E-dien-1-oic acid | 2,3-dinor TXB2 | 63250-09-9 | C18H30O6 | 342.4 |
| 2,3-dinor-11β-PGF2α | 9α,11β,15S-trihydroxy-2,3-dinor-prosta-5Z,13E-dien-1-oic acid | 2,3-dinor-11β-PGF2α | 240405-20-3 | C18H30O5 | 326.4 |
| 2,3-dinor-8-iso-PGF2α | 9α,11α,15S-trihydroxy-2,3-dinor-(8β)-prosta-5Z,13E-dien-1-oic acid | 2,3-dinor-8-iso-PGF2α | 221664-05-7 | C18H30O5 | 326.4 |
| 20-carboxy LTB4 | 5S,12R-dihydroxy-6Z,8E,10E,14Z-eicosatetraene-1,20-dioic acid | 20-carboxy LTB4 | 80434-82-8 | C20H30O6 | 366.5 |
| 20-COOH-AA | 5Z,8Z,11Z,14Z-eicosatetraenedioic acid | 20-COOH-AA | 79551-84-1 | C20H30O4 | 334.4 |
| 20-HETE | 20-hydroxy-5Z,8Z,11Z,14Z-eicosatetraenoic acid | 20-HETE | 79551-86-3 | C20H32O3 | 320.5 |
| 20-hydroxy LTB4 | 5S,12R,20-trihydroxy-6Z,8E,10E,14Z-eicosatetraenoic acid | 20-hydroxy LTB4 | 79516-82-8 | C20H32O5 | 352.5 |
| 5-OxoETE | 5-oxo-6E,8Z,11Z,14Z-eicosatetraenoic acid | 5-OxoETE | 106154-18-1 | C20H30O3 | 318.5 |
| 5S,6R-DiHETE | 5S,6R-dihydroxy-7E,9E,11Z,14Z-eicosatetraenoic acid | 5S,6R-DiHETE | 82948-88-7 | C20H32O4 | 336.5 |
| 5S-HEPE | 5S-hydroxy-6E,8Z,11Z,14Z,17Z-eicosapentaenoic acid | 5S-HEPE | 92008-51-0 | C20H30O3 | 318.5 |
| 5S-HETrE | 5S-hydroxy-6E,8Z,11Z-eicosatrienoic acid | 5S-HETrE | 195061-94-0 | C20H34O3 | 322.5 |
| 6-keto PGF1α | 6-oxo-9α,11α,15S-trihydroxy-prost-13E-en-1-oic acid | 6-keto PGF1α | 58962-34-8 | C20H34O6 | 370.5 |
| 6S-LXA4 | 5S,6S,15S-trihydroxy-7E,9E,11Z,13E-eicosatetraenoic acid | 6S-LXA4 | 94292-80-5 | C20H32O5 | 352.5 |
| 6-trans LTB4 | 5S,12R-dihydroxy-6E,8E,10E,14Z-eicosatetraenoic acid | 6-trans LTB4 | 71652-82-9 | C20H32O4 | 336.5 |
| 8-iso PGF2α | 9α,11α,15S-trihydroxy-(8β)-prosta-5Z,13E-dien-1-oic acid | 8-iso PGF2α | 27415-26-5 | C20H34O5 | 354.5 |
| 8-iso-15-keto PGF2β | 9β,11α-dihydroxy-15-oxo-(8β)-prosta-5Z,13E-dien-1-oic acid | 8-iso-15-keto PGF2β | 1621482-36-7 | C20H32O5 | 352.5 |
| 8S,15S-DiHETE | 8S,15S-dihydroxy-5Z,9E,11Z,13E-eicosatetraenoic acid | 8S,15S-DiHETE | 80234-65-7 | C20H32O4 | 336.5 |
| 8S-HETrE | 8S-hydroxy-9E,11Z,14Z-eicosatrienoic acid | 8S-HETrE | 889573-69-7 | C20H34O3 | 322.5 |
| 9-Nitrooleic Acid | 9-nitro-9E-octadecenoic acid | 9-Nitrooleic Acid | 875685-44-2 | C18H33NO4 | 327.5 |
| 9-OxoODE | 9-oxo-10E,12Z-octadecadienoic acid | 9-OxoODE | 54232-59-6 | C18H30O3 | 294.4 |
| 9R-HETE | 9R-hydroxy-5Z,7E,11Z,14Z-eicosatetraenoic acid | 9R-HETE | 107656-14-4 | C20H32O3 | 320.5 |
| 9S-HODE | 9S-hydroxy-10E,12Z-octadecadienoic acid | 9S-HODE | 73543-67-6 | C18H32O3 | 296.5 |
| 9S-HOTrE | 9S-hydroxy-10E,12Z,15Z-octadecatrienoic acid | 9S-HOTrE | 89886-42-0 | C18H30O3 | 294.4 |
| ARA | 5Z,8Z,11Z,14Z-eicosatetraenoic acid | ARA | 506-32-1 | C20H32O2 | 304.5 |
| DHA | 4Z,7Z,10Z,13Z,16Z,19Z-docosahexaenoic acid | DHA | 6217-54-5 | C22H32O2 | 328.5 |
| DTA | 7Z,10Z,13Z,16Z-docosatetraenoic acid | DTA | 28874-58-0 | C22H36O2 | 332.5 |
| EPA | 5Z,8Z,11Z,14Z,17Z-eicosapentaenoic acid | EPA | 10417-94-4 | C20H30O2 | 302.5 |
| LTE4 | 5S-hydroxy-6R-(S-cysteinyl)-7E,9E,11Z,14Z-eicosatetraenoic acid | LTE4 | 75715-89-8 | C23H37NO5S | 439.6 |
| LXA5 | 5S,6R,15S-trihydroxy-7E,9E,11Z,13E,17Z-eicosapentaenoic acid | LXA5 | 110657-98-2 | C20H30O5 | 350.5 |
| PGA2 | 9-oxo-15S-hydroxy-prosta-5Z,10,13E-trien-1-oic acid | PGA2 | 13345-50-1 | C20H30O4 | 334.4 |
| PGB2 | 9-oxo-15S-hydroxy-prosta-5Z,8(12),13E-trien-1-oic acid | PGB2 | 13367-85-6 | C20H30O4 | 334.4 |
| PGD1 | 9α,15S-dihydroxy-11-oxo-prost-13E-en-1-oic acid | PGD1 | 17968-82-0 | C20H34O5 | 354.5 |
| PGD2 | 9α,15S-dihydroxy-11-oxo-prosta-5Z,13E-dien-1-oic acid | PGD2 | 41598-07-6 | C20H32O5 | 352.5 |
| PGD3 | 9α,15S-dihydroxy-11-oxo-prosta-5Z,13E,17Z-trien-1-oic acid | PGD3 | 71902-47-1 | C20H30O5 | 350.5 |
| PGE1 | 9-oxo-11α,15S-dihydroxy-prost-13E-en-1-oic acid | PGE1 | 745-65-3 | C20H34O5 | 354.5 |
| PGE3 | 9-oxo-11α,15S-dihydroxy-prosta-5Z,13E,17Z-trien-1-oic acid | PGE3 | 802-31-3 | C20H30O5 | 350.5 |
| PGF1α | 9α,11α,15S-trihydroxy-prost-13E-en-1-oic acid | PGF1α | 745-62-0 | C20H36O5 | 356.5 |
| PGF2α | 9α,11α,15S-trihydroxy-prosta-5Z,13E-dien-1-oic acid | PGF2α | 551-11-1 | C20H34O5 | 354.5 |
| PGF3α | 9α,11α,15S-trihydroxy-prosta-5Z,13E,17Z-trien-1-oic acid | PGF3α | 745-64-2 | C20H32O5 | 352.5 |
| PGJ2 | 11-oxo-15S-hydroxy-prosta-5Z,9,13E-trien-1-oic acid | PGJ2 | 60203-57-8 | C20H30O4 | 334.4 |
| tetranor-12S-HETE | 8S-hydroxy-4Z,6E,10Z-hexadecatrienoic acid | tetranor-12S-HETE | 121842-79-3 | C16H26O3 | 266.4 |
| TXB1 | 9,11,15-trihydroxy-thrombox-13-en-1-oic acid | TXB1 | 64626-32-0 | C20H36O6 | 372.5 |
| TXB2 | 9α,11,15S-trihydroxythromba-5Z,13E-dien-1-oic acid | TXB2 | 54397-85-2 | C20H34O6 | 370.5 |
| TXB3 | 9α,11,15S-trihydroxy-thromba-5Z,13E,17Z-trien-1-oic acid | TXB3 | 71953-80-5 | C20H32O6 | 368.5 |
| Δ17-6-keto PGF1α | 6-oxo-9α,11α,15S-trihydroxy-prosta-13E,17Z-dien-1-oic acid | Δ17-6-keto PGF1α | 68324-95-8 | C20H32O6 | 368.5 |
