# Supplementary material for: SIRT1 deficiency promotes age-related heart failure through enhancing ferroptosis via GATA4-HADHA-GPX4 axis
Source: Cell Death Dis. 2026 Mar 23;17(1):343. doi: 10.1038/s41419-026-08634-z (PMC13039550; doi:10.1038/s41419-026-08634-z)
Supplement: Supplementary file 3 — supplement table2-Echocardiographic analysis of rats or mice [file 41419_2026_8634_MOESM3_ESM.docx]

**Supplemental Table 2. Echocardiographic analysis of rats or mice**

|  | IVSd(cm) | IVSs(cm) | LVIDd(cm) | LVIDs(cm) | LVPWd(cm) | LVPWs(cm) |
| --- | --- | --- | --- | --- | --- | --- |
| Young vs Aging | 0.128±0.003 vs  0.143±0.005*** | 0.199±0.016 vs  0.230±0.017* | 0.673±0.061 vs  0.822±0.077* | 0.397±0.052 vs  0.474±0.073 | 0.128±0.003 vs  0.139±0.008* | 0.216±0.024 vs  0.237±0.013 |
| GPX4^flox/flox^ vs GPX4-cKO | 0.051±0.001 vs  0.060±0.002*** | 0.095±0.004 vs  0.104±0.003* | 0.336±0.018 vs  0.393±0.022* | 0.206±0.005 vs  0.227±0.018 | 0.083±0.003 vs  0.088±0.004 | 0.107±0.003 vs  0.112±0.007 |
| Control vs OE-GPX4 | 0.053±0.002 vs  0.051±0.003 | 0.085±0.004 vs  0.082±0.005 | 0.359±0.017 vs  0.343±0.019 | 0.215±0.008 vs  0.207±0.011 | 0.091±0.002 vs  0.087±0.003 | 0.113±0.005 vs  0.102±0.008 |
| Aging ND vs Aging HID | 0.144±0.004 vs  0.142±0.003 | 0.228±0.016 vs  0.223±0.008 | 0.806±0.032 vs  0.850±0.093 | 0.497±0.052 vs  0.562±0.090 | 0.138±0.007 vs  0.146±0.004 | 0.229±0.006 vs  0.224±0.019 |
| Aging Control vs Aging Fer-1 | 0.141±0.004 vs  0.139±0.005 | 0.225±0.013 vs  0.224±0.011 | 0.826±0.065 vs  0.819±0.063 | 0.492±0.068 vs  0.512±0.081 | 0.141±0.006 vs  0.140±0.007 | 0.231±0.014 vs  0.228±0.017 |
| Young ND vs Young HID | 0.126±0.004 vs  0.128±0.003 | 0.208±0.018 vs  0.215±0.021 | 0.603±0.049 vs  0.622±0.067 | 0.388±0.061 vs  0.392±0.068 | 0.125±0.005 vs  0.128±0.006 | 0.218±0.018 vs  0.220±0.016 |
| Control vs Sh-HADHA vs Sh-HADHA+Fer1 | 0.049±0.002 vs  0.053±0.004 vs  0.051±0.004 | 0.079±0.005 vs  0.080±0.003 vs  0.081±0.004 | 0.349±0.015 vs  0.351±0.012 vs  0.352±0.016 | 0.207±0.005 vs  0.216±0.008 vs  0.217±0.009 | 0.082±0.003 vs  0.089±0.004 vs  0.088±0.004 | 0.108±0.007 vs  0.111±0.003 vs  0.110±0.005 |
| Aging Control vs Aging NAC | 0.139±0.007 vs  0.140±0.005 | 0.227±0.015 vs  0.228±0.013 | 0.818±0.071 vs  0.820±0.064 | 0.502±0.063 vs  0.515±0.079 | 0.140±0.005 vs  0.141±0.006 | 0.224±0.016 vs  0.225±0.009 |
| D-Gal Control vs D-Gal NAC | 0.131±0.006 vs  0.129±0.004 | 0.211±0.012 vs  0.206±0.016 | 0.651±0.056 vs  0.635±0.053 | 0.403±0.057vs  0.392±0.062 | 0.132±0.005 vs  0.127±0.006 | 0.221±0.013 vs  0.217±0.015 |
| Aging Control vs Aging RES | 0.142±0.005 vs  0.140±0.006 | 0.230±0.019 vs  0.226±0.014 | 0.823±0.070 vs  0.819±0.059 | 0.514±0.072 vs  0.502±0.083 | 0.142±0.005 vs  0.139±0.007 | 0.235±0.019 vs  0.231±0.008 |
| Control vs OE-SIRT1 | 0.052±0.003 vs  0.050±0.002 | 0.086±0.003 vs  0.084±0.002 | 0.357±0.015 vs  0.353±0.013 | 0.212±0.012 vs  0.210±0.009 | 0.092±0.003 vs  0.091±0.002 | 0.114±0.007 vs  0.106±0.007 |

**P* < 0.05, and ****P* < 0.001 by unpaired Student’s t-test.

IVSd, interventricular septum thickness in diastole; IVSs, interventricular septum thickness in systole; LVIDd, left ventricle internal diameter in diastole; LVIDs, left ventricle internal diameter in systole; LVPWd, left ventricular posterior wall thickness in diastole; LVPWs, left ventricular posterior wall thickness in systole.
