# Supplementary material for: SIRT1 deficiency promotes age-related heart failure through enhancing ferroptosis via GATA4-HADHA-GPX4 axis
Source: Cell Death Dis. 2026 Mar 23;17(1):343. doi: 10.1038/s41419-026-08634-z (PMC13039550; doi:10.1038/s41419-026-08634-z)

Figure 2F

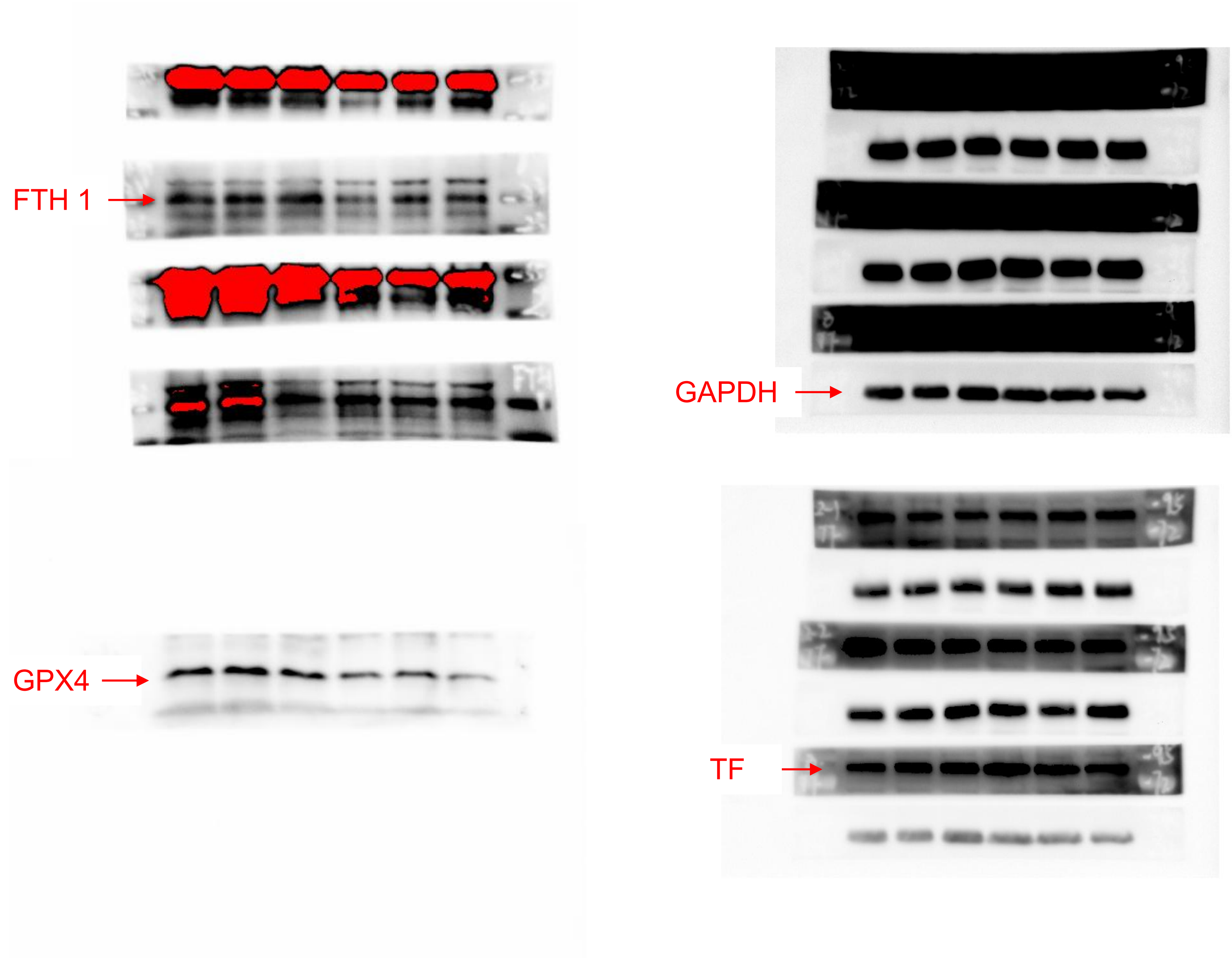

Figure 2L

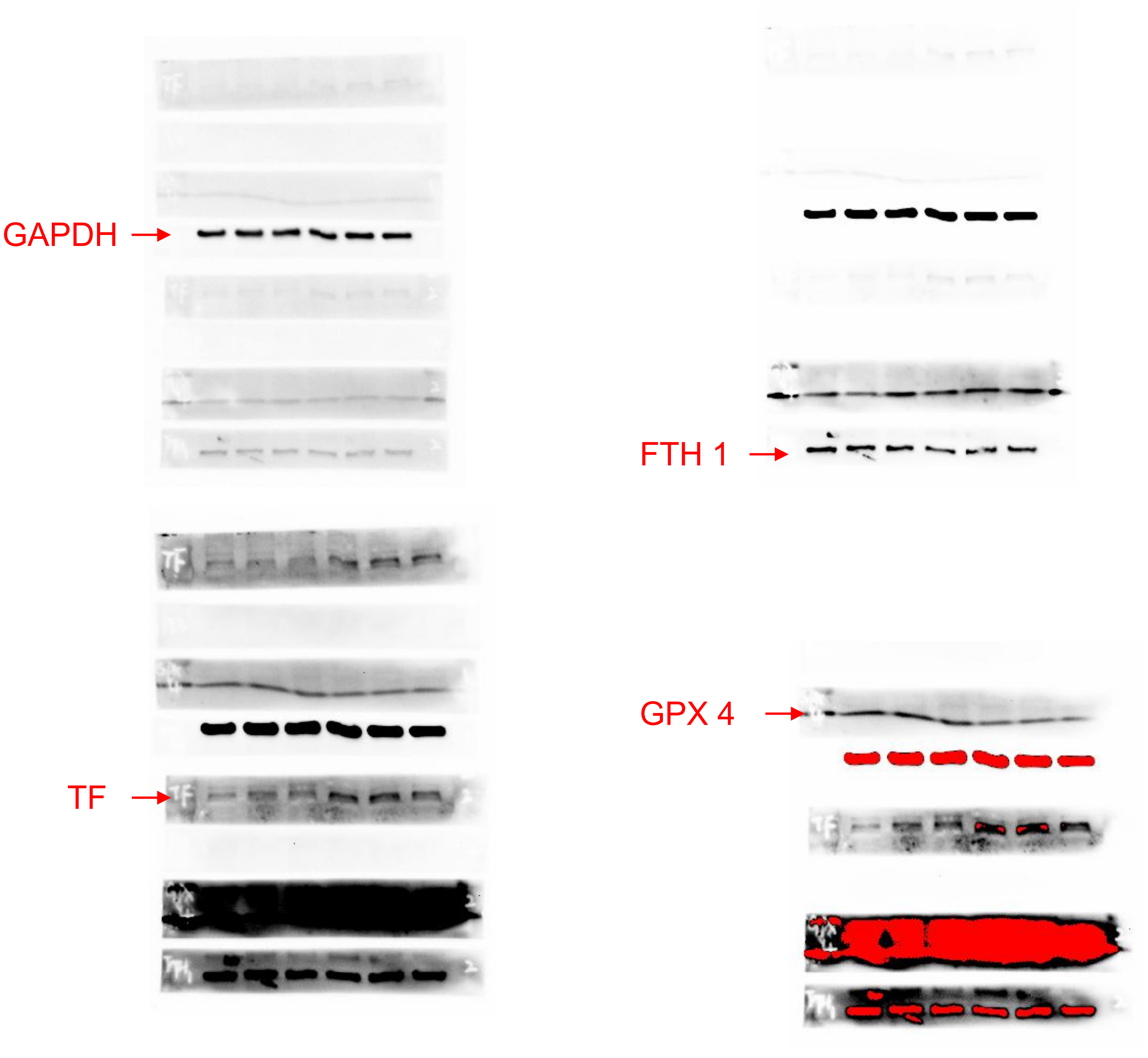

Supplement Figure 3D

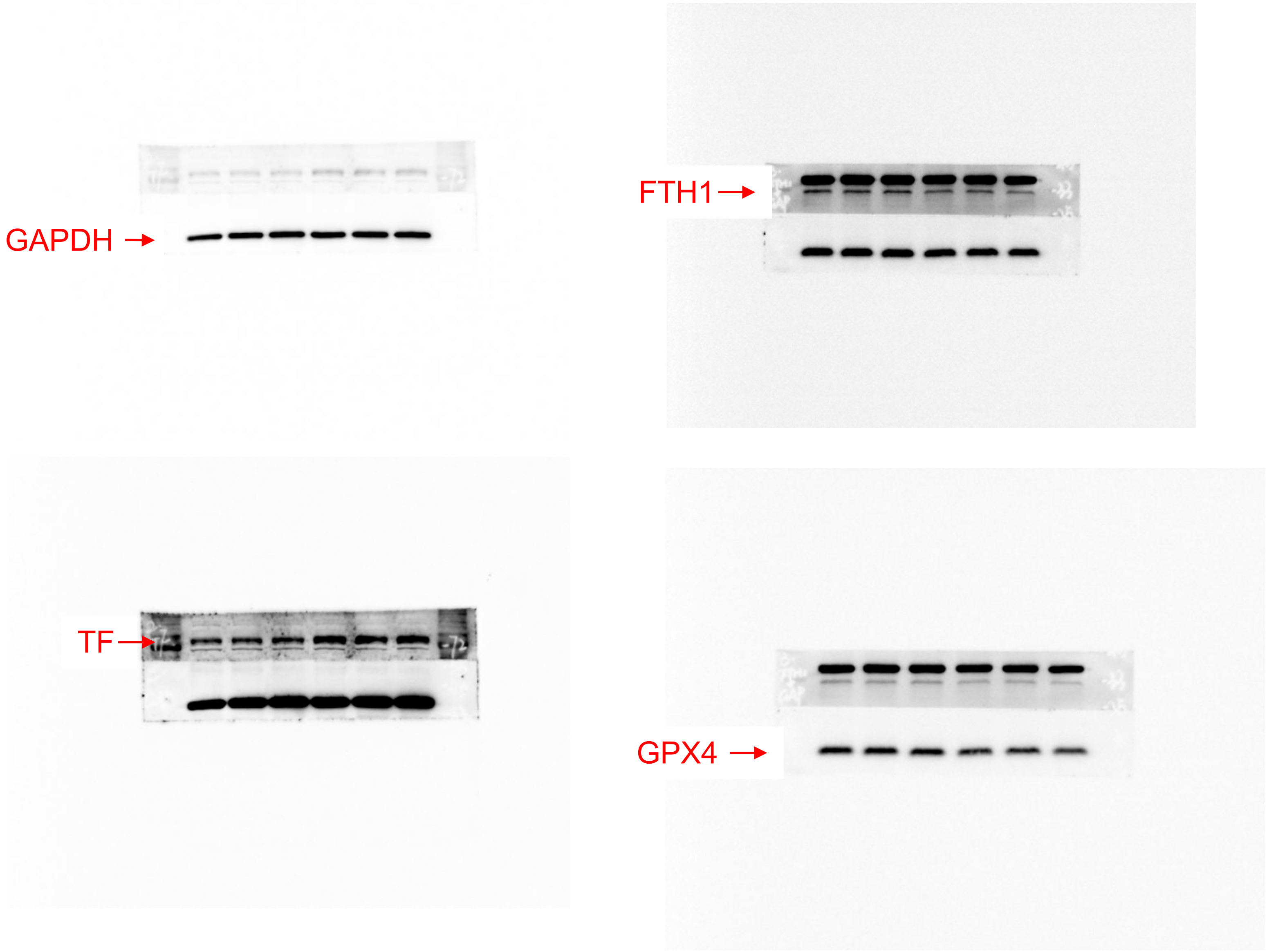

Figure 3B

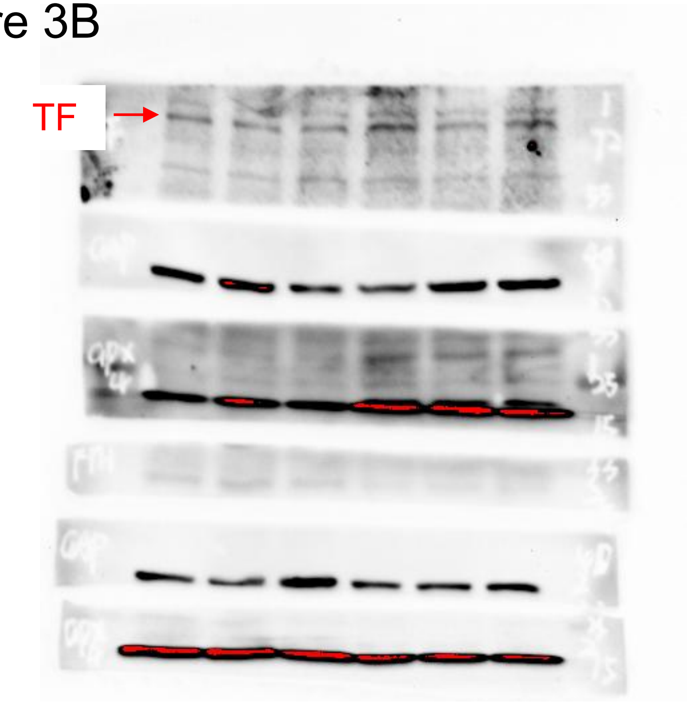

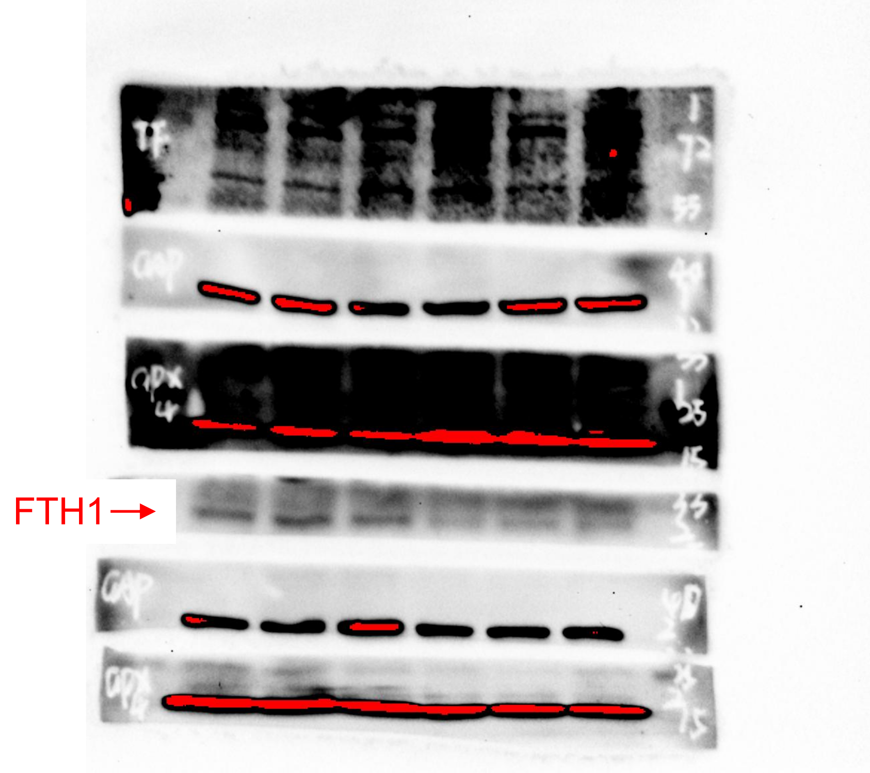

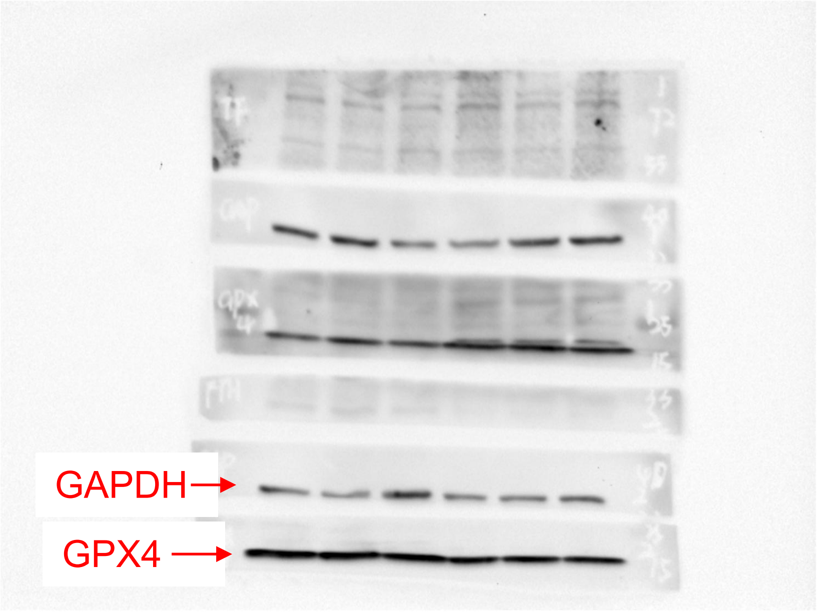

Supplement Figure 4F

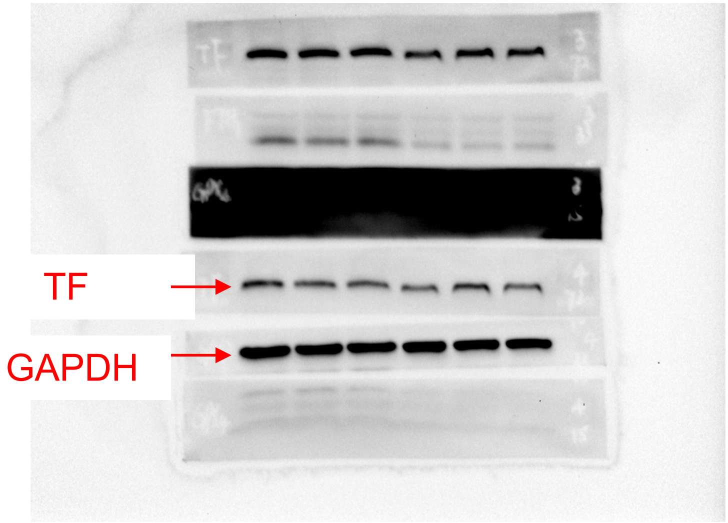

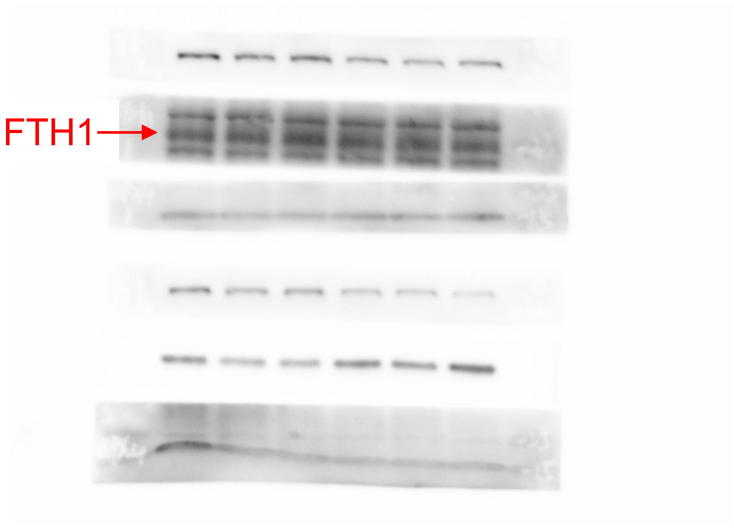

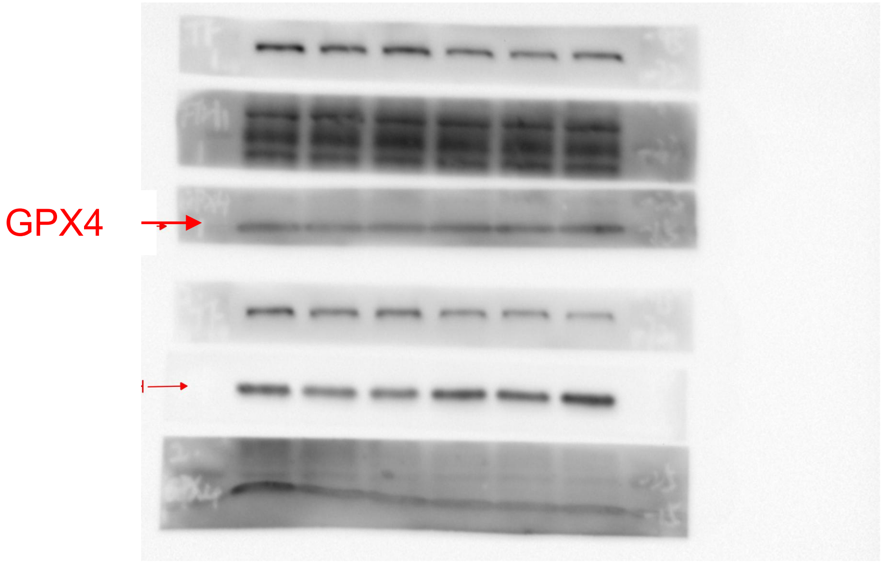

Supplement Figure 5B

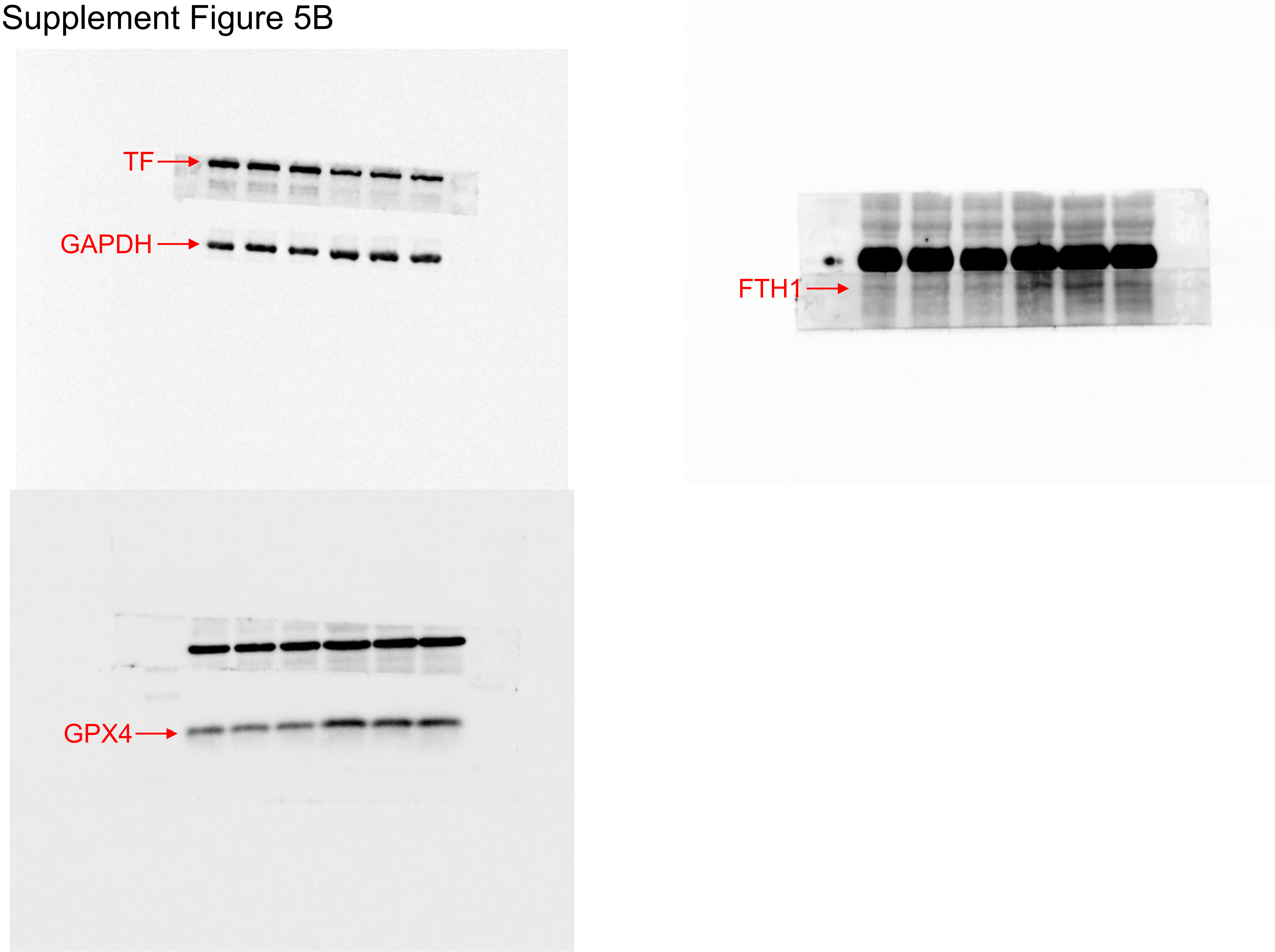

Figure 4D

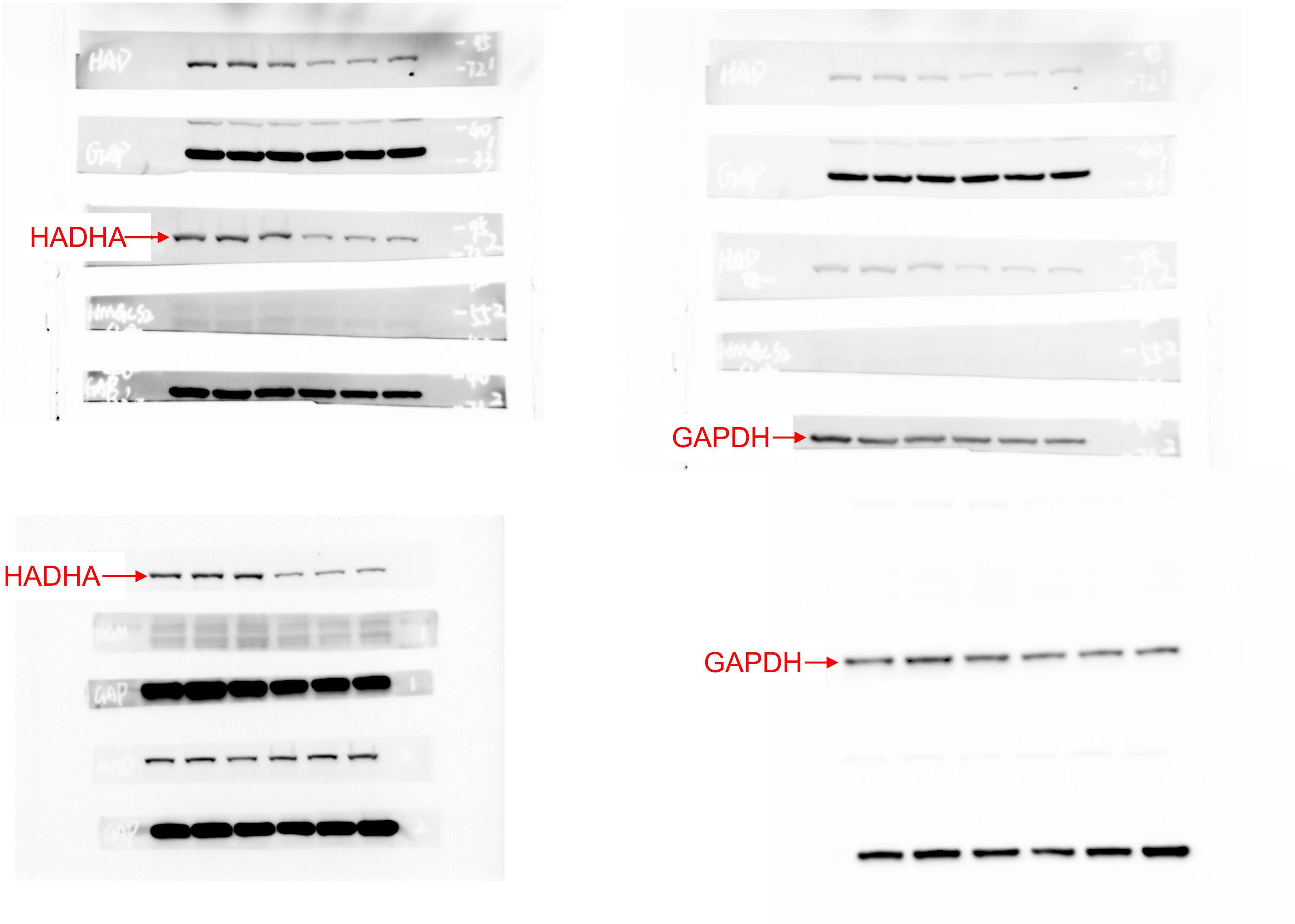

Figure 4J

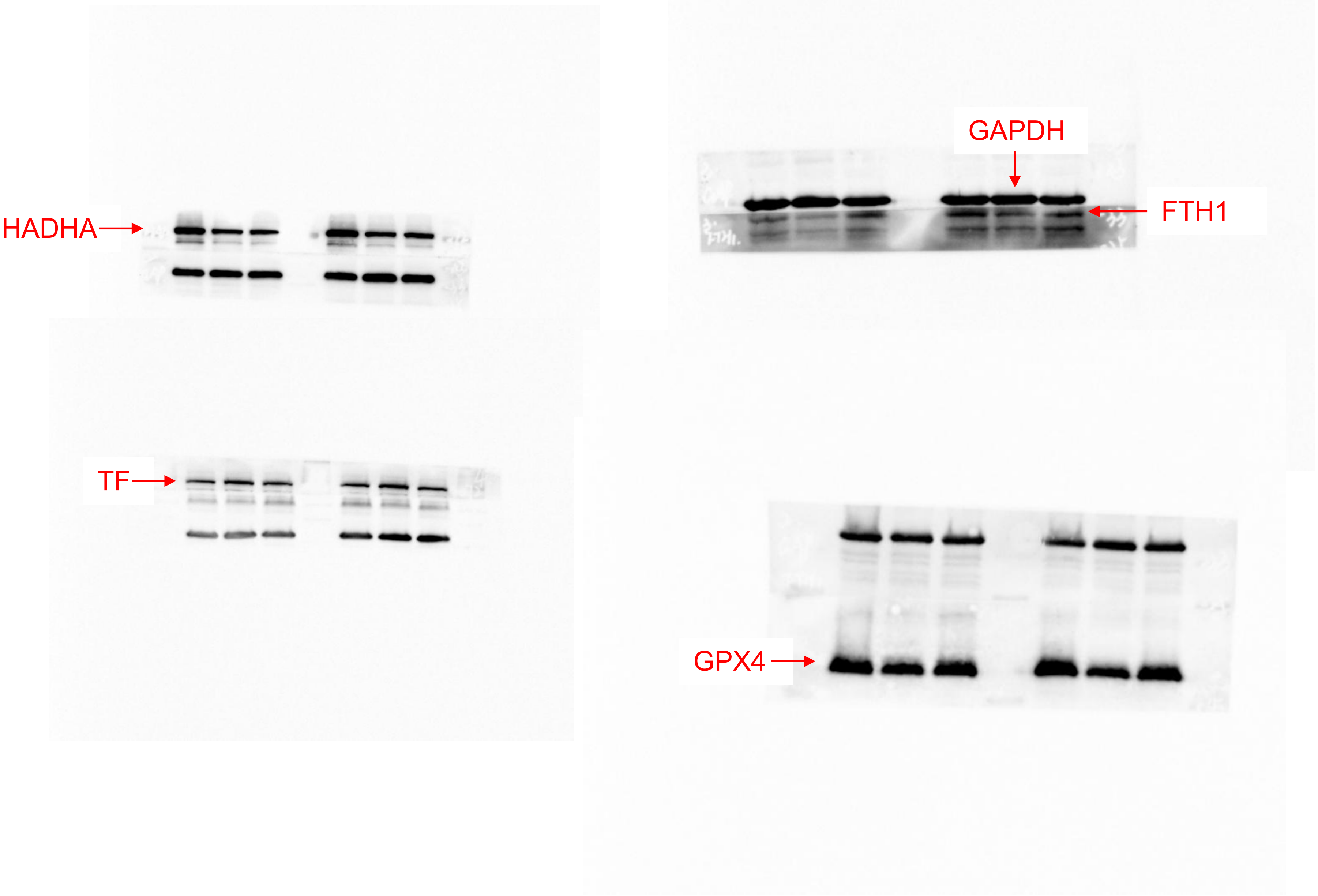

Supplement Figure 7F

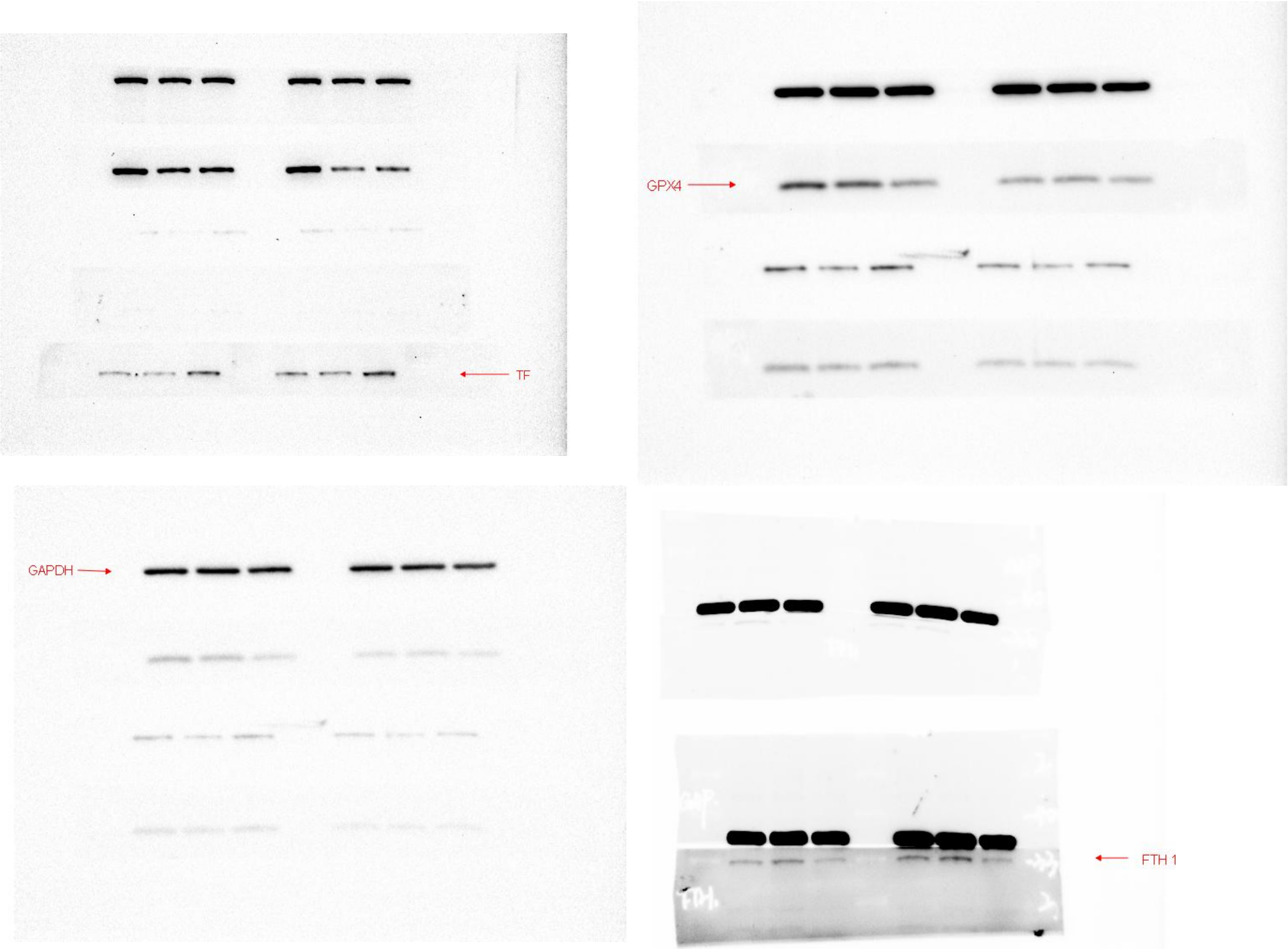

Supplement Figure 8A

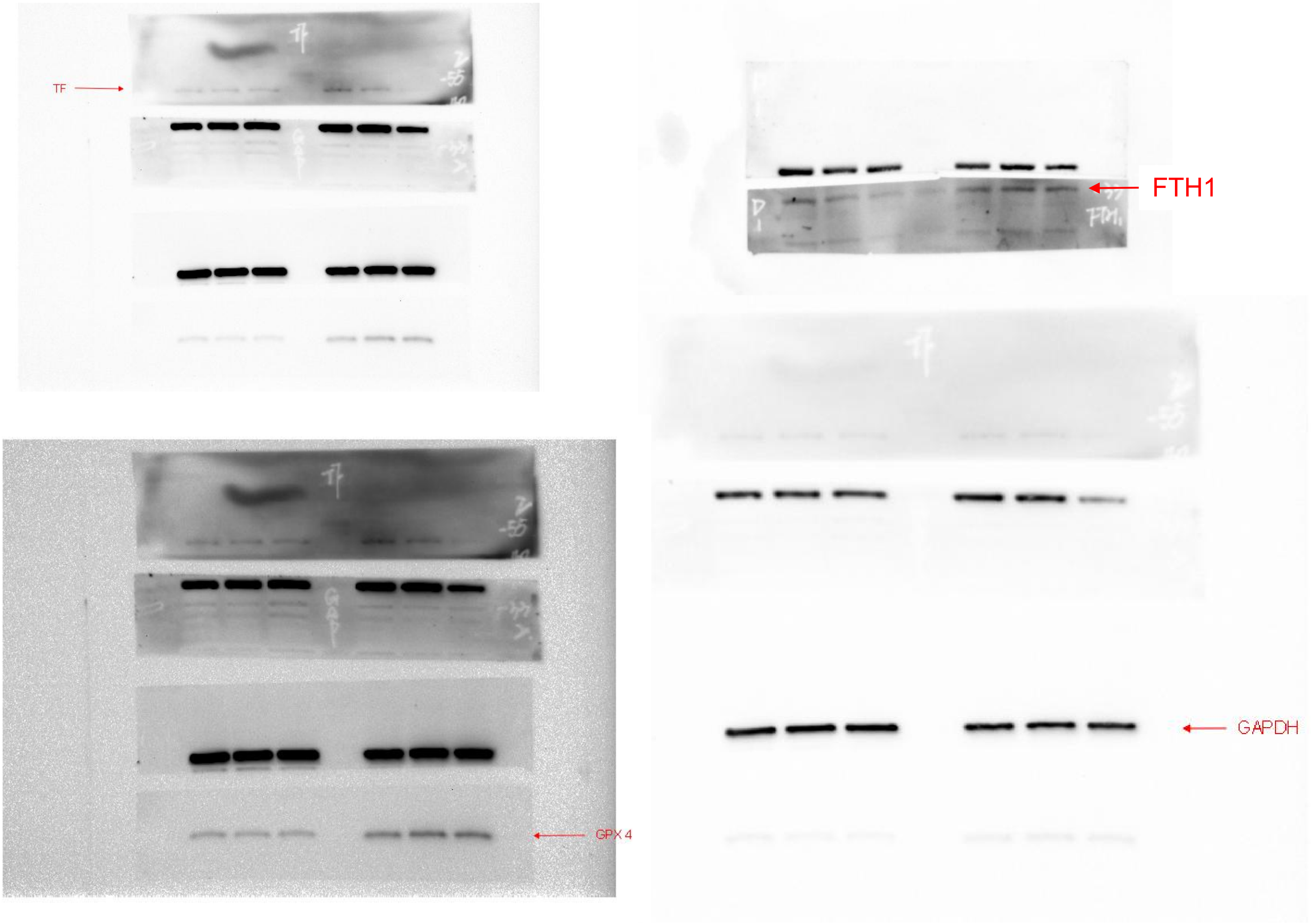

Figure 5D

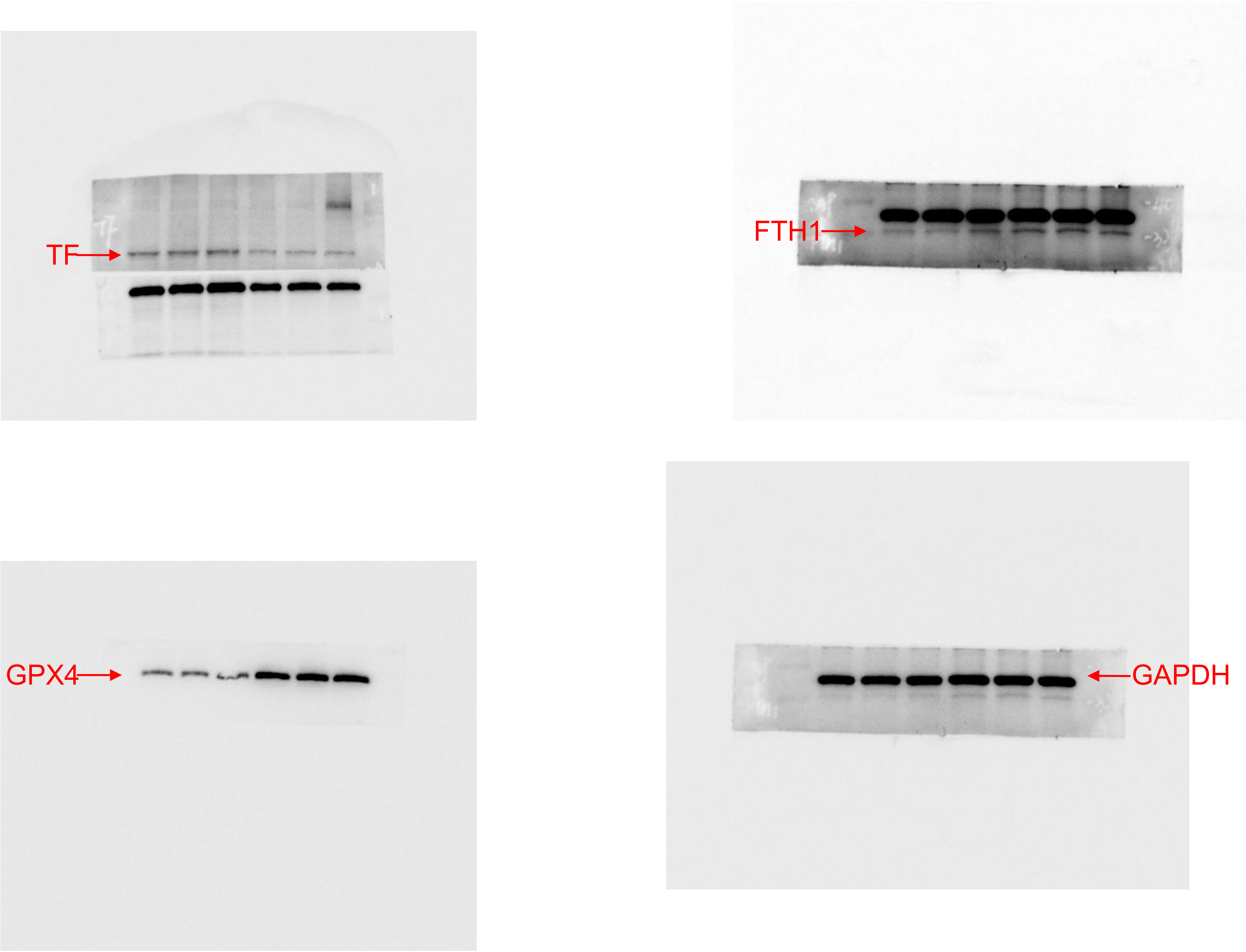

Figure 5F

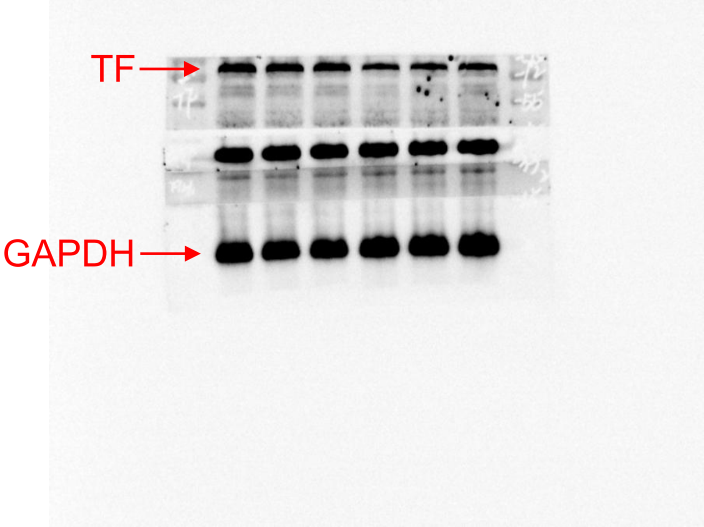

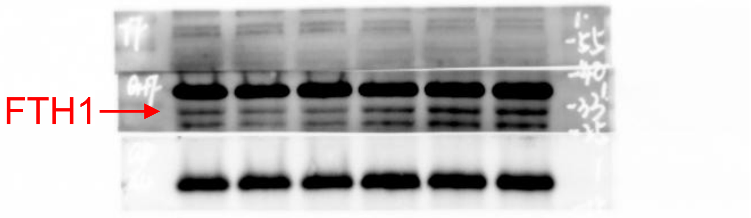

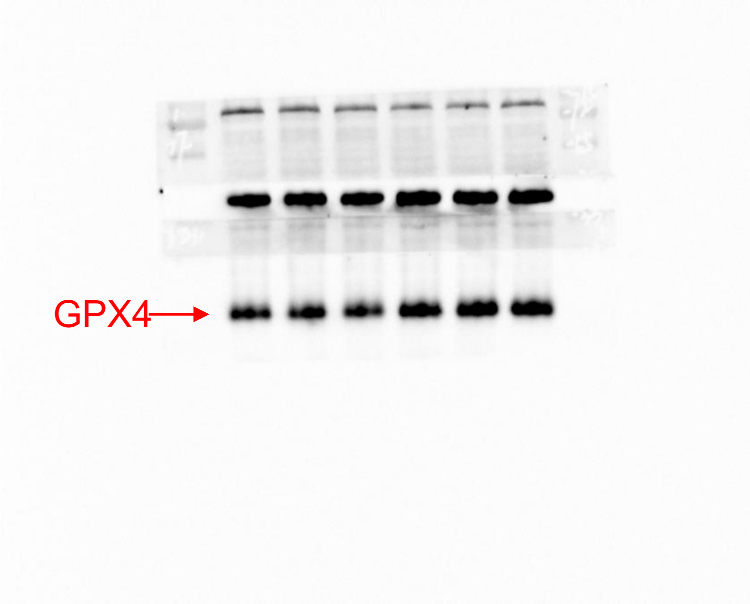

Supplement Figure 10B

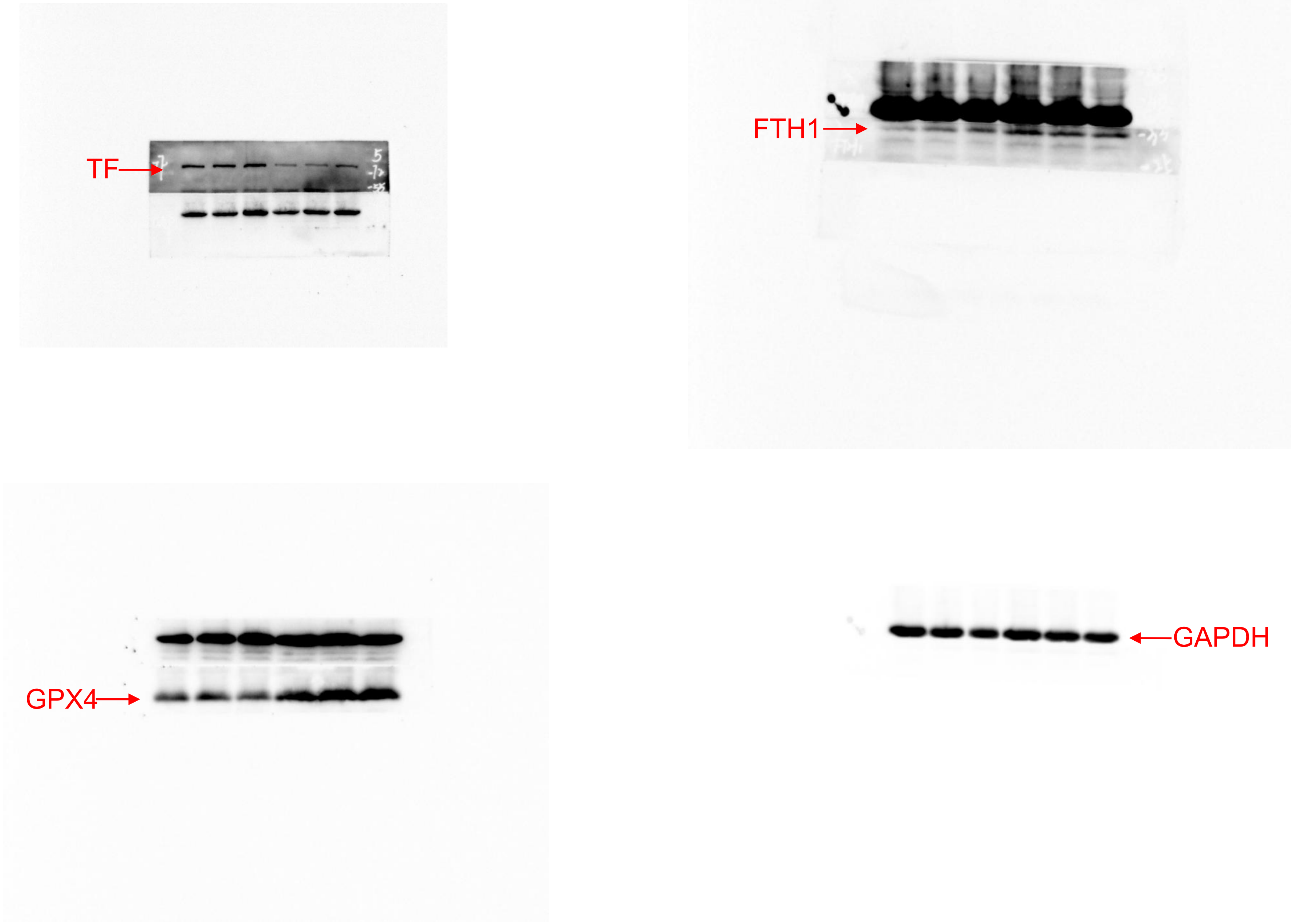

Figure 6A

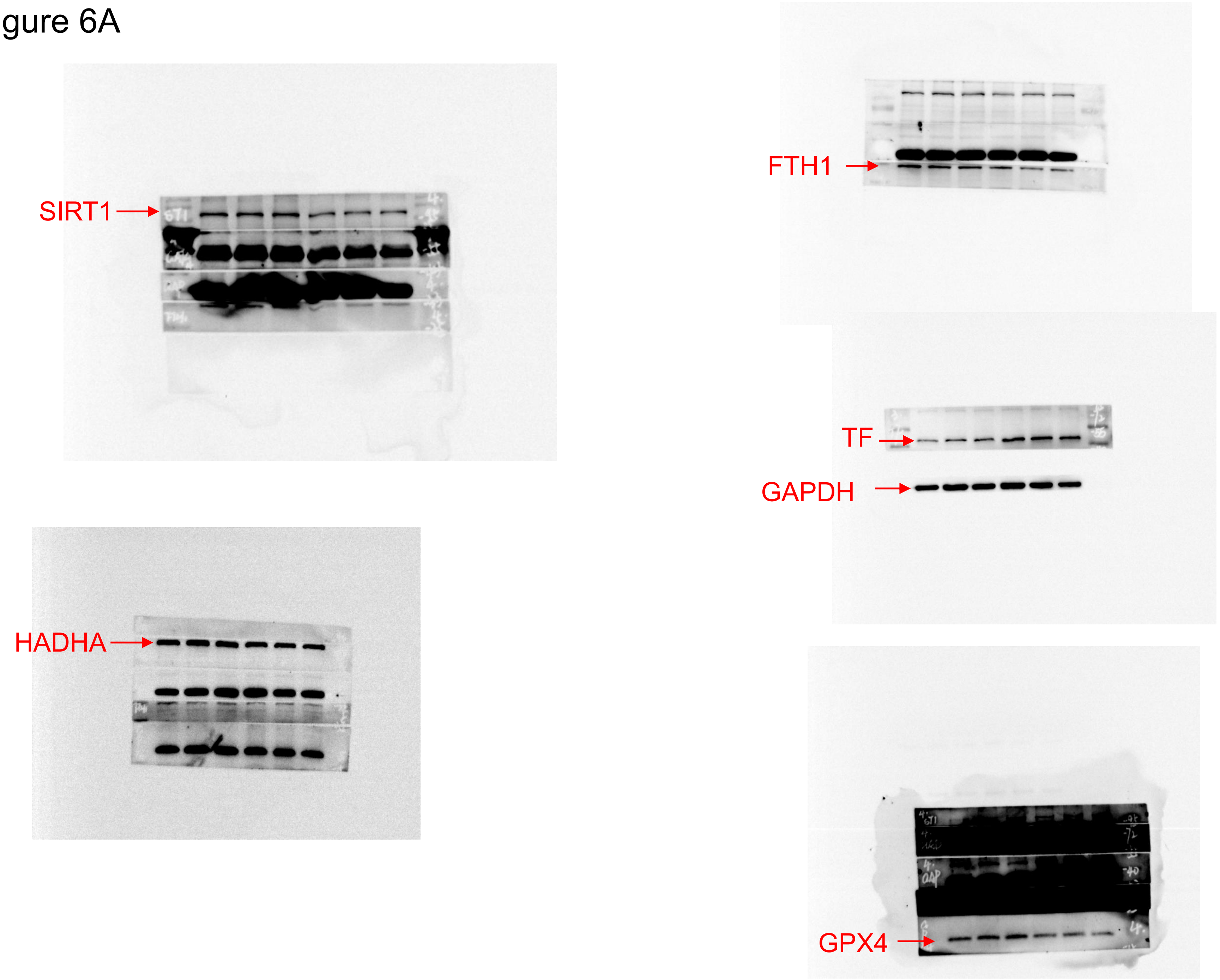

Figure 6E

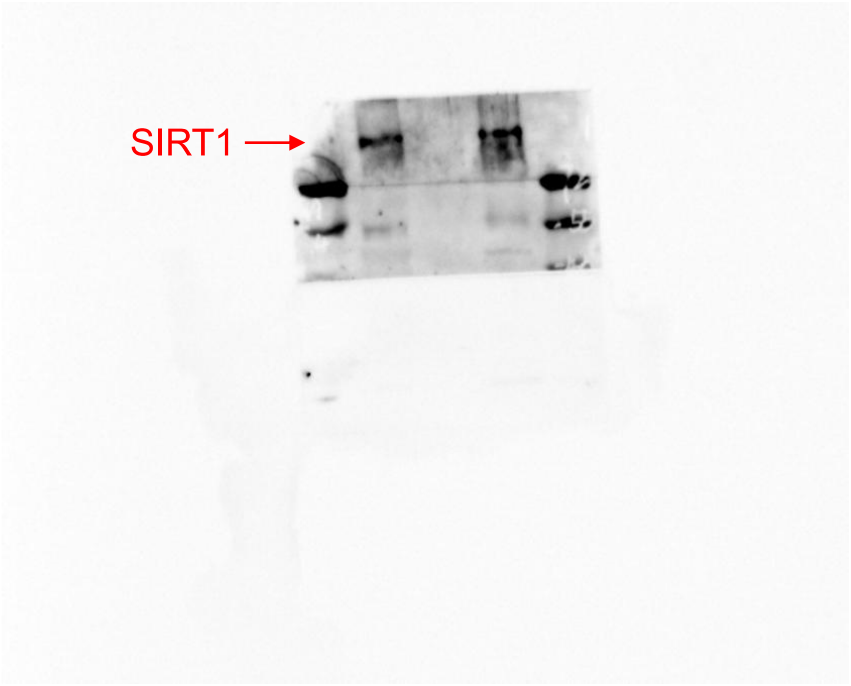

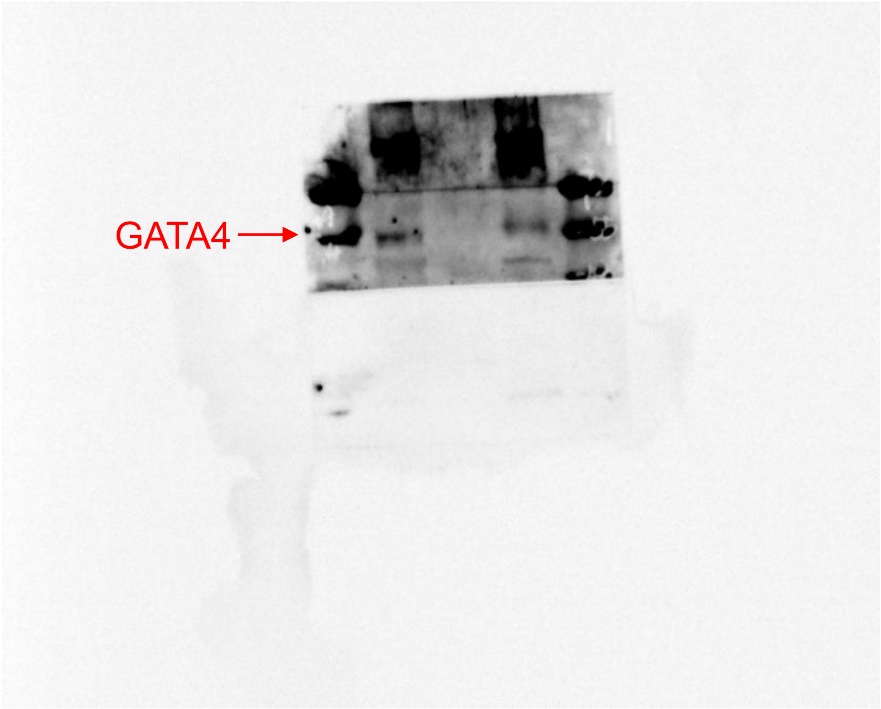

Figure 6I

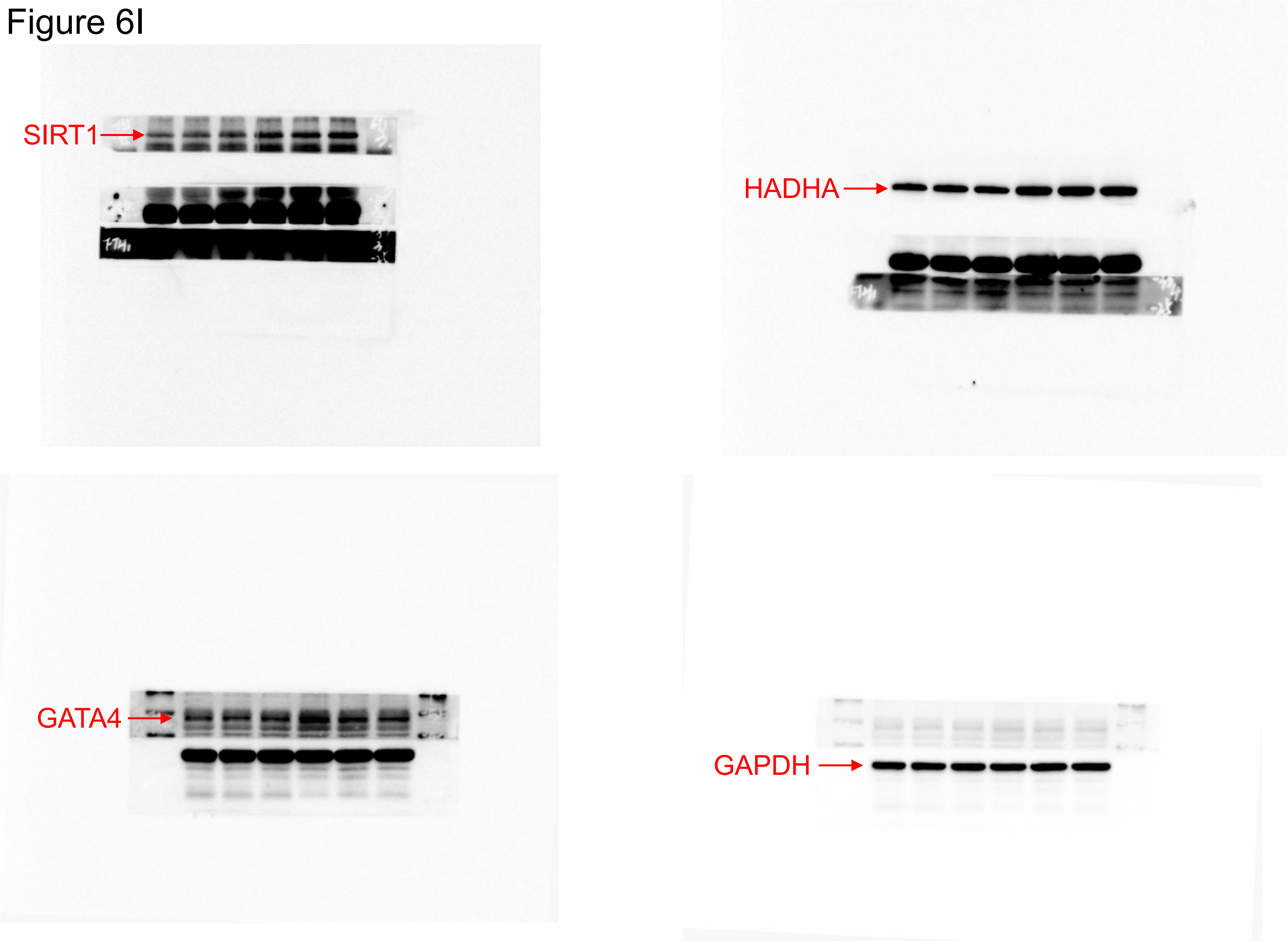

Figure 6J

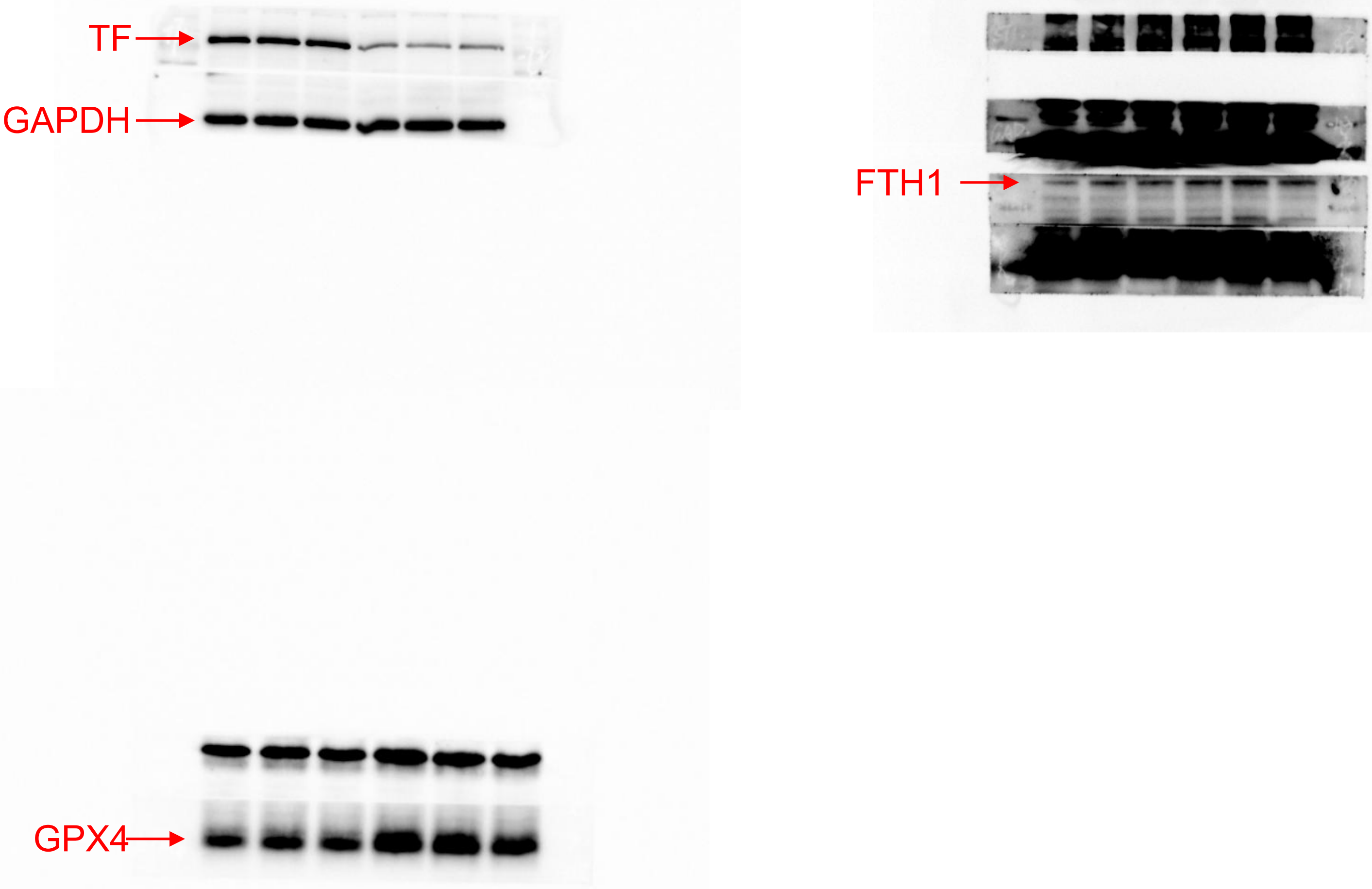

Supplement Figure 11B

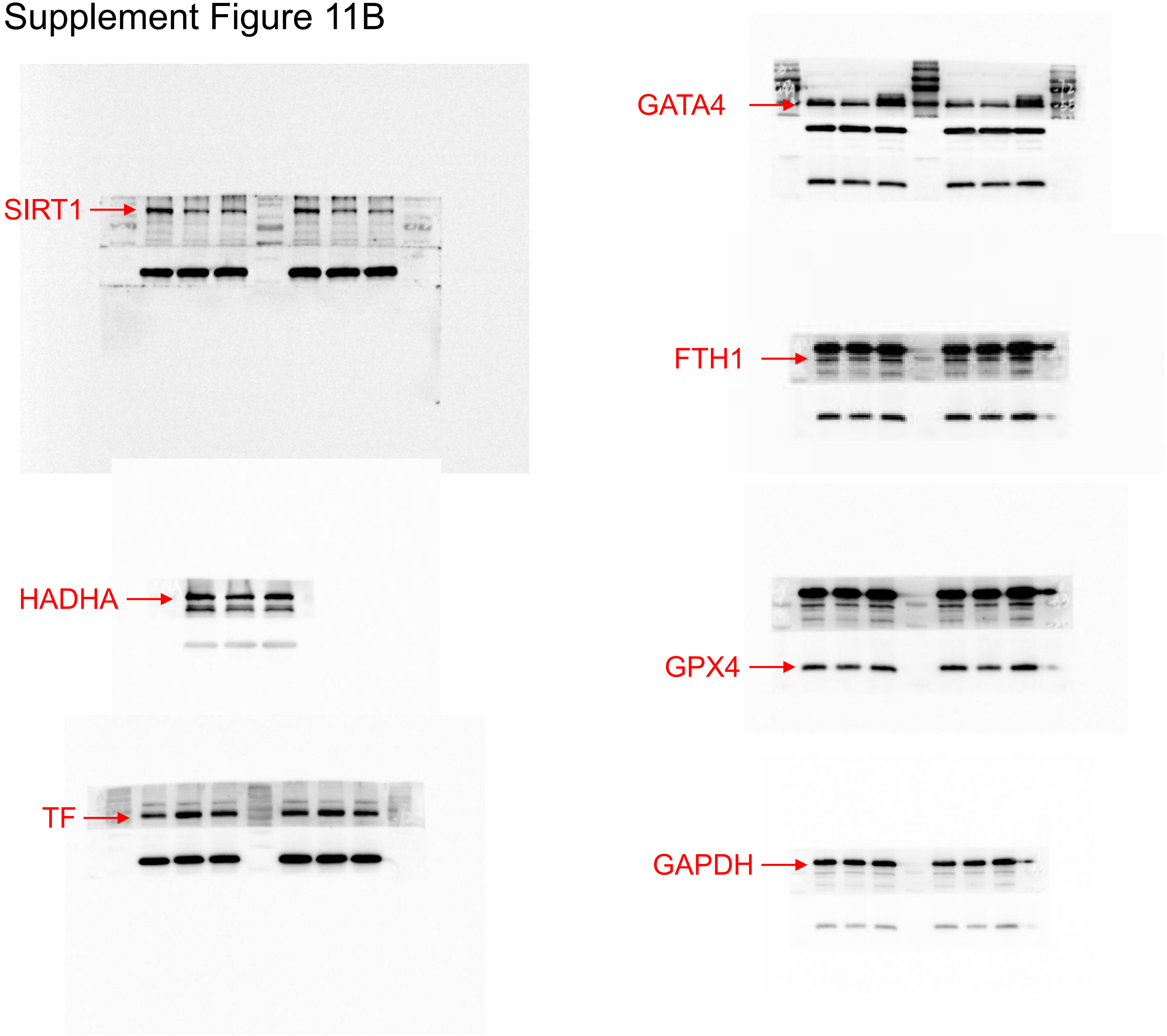

Supplement Figure 12H

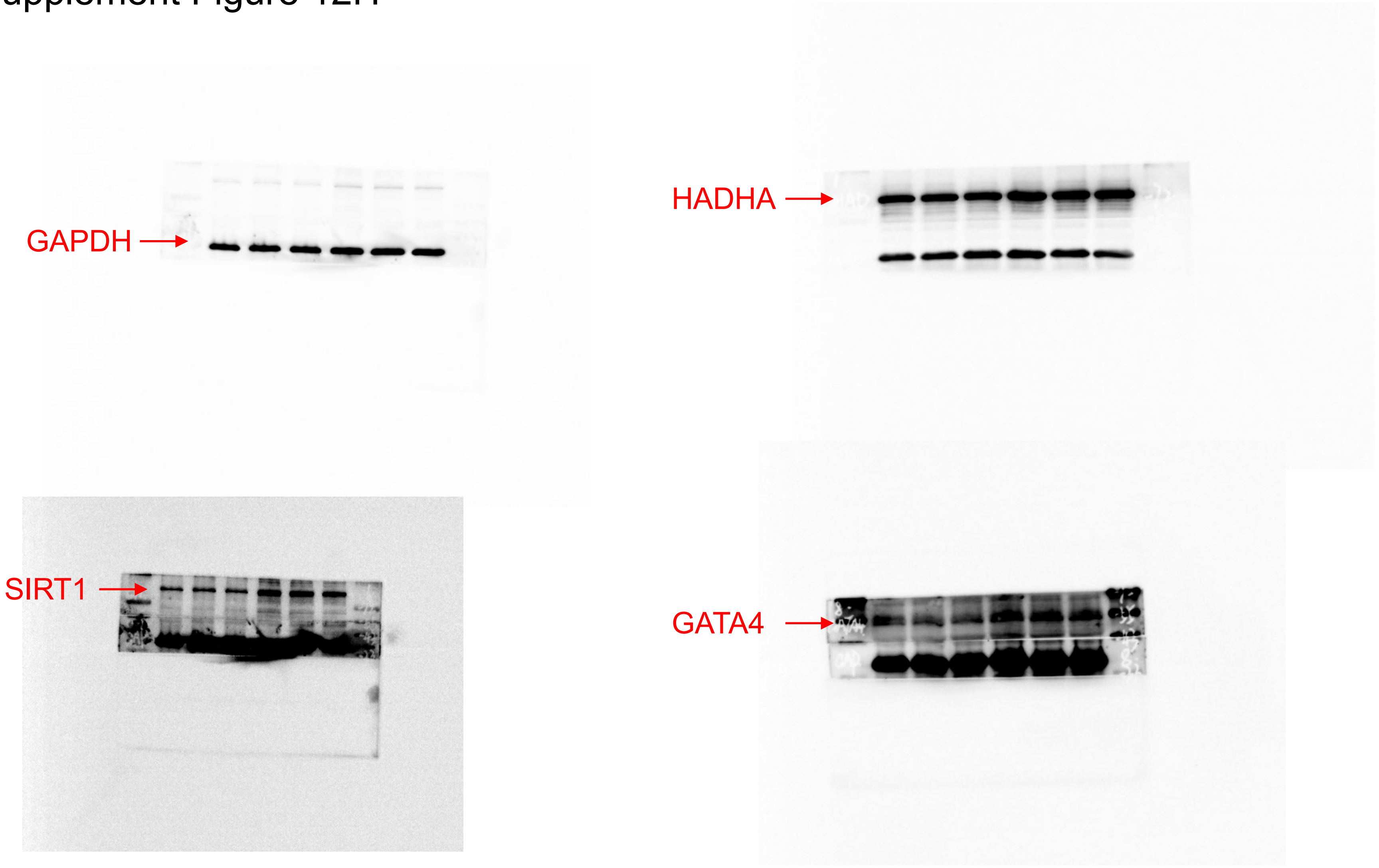

Supplement Figure 12I

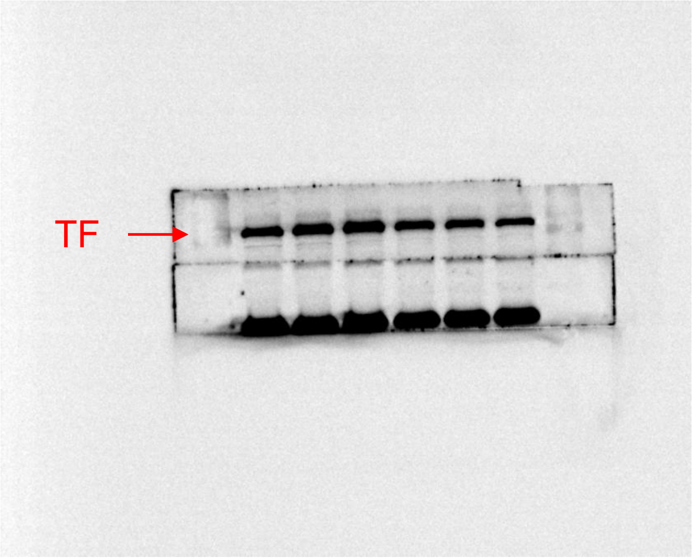

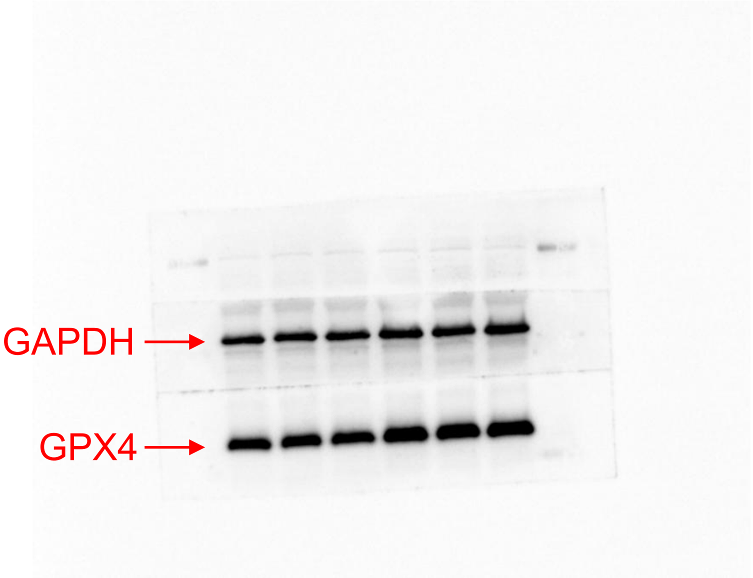

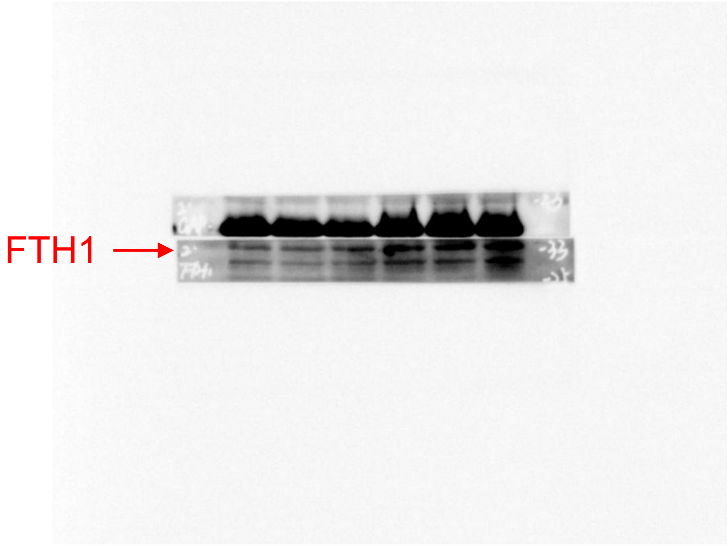

Supplement: Supplementary file 4 — uncropped blots [file 41419_2026_8634_MOESM4_ESM.pdf]
